# Supplementary material for: CRISPR-Mediated Protein Tagging with Nanoluciferase to Investigate Native Chemokine Receptor Function and Conformational Changes
Source: Cell Chem Biol. 2020 May 21;27(5):499–510.e7. doi: 10.1016/j.chembiol.2020.01.010 (PMC7242902; doi:10.1016/j.chembiol.2020.01.010)
Supplement: Document S1. Figures S1–S6 and Table S1 [file mmc1.pdf]

Cell Chemical Biology, Volume *27*

Supplemental Information

CRISPR-Mediated Protein Tagging with Nanoluciferase to Investigate Native Chemokine Receptor Function and Conformational Changes

Carl W. White, Birgit Caspar, Hannah K. Vanyai, Kevin D.G. Pfleger, and Stephen J. Hill

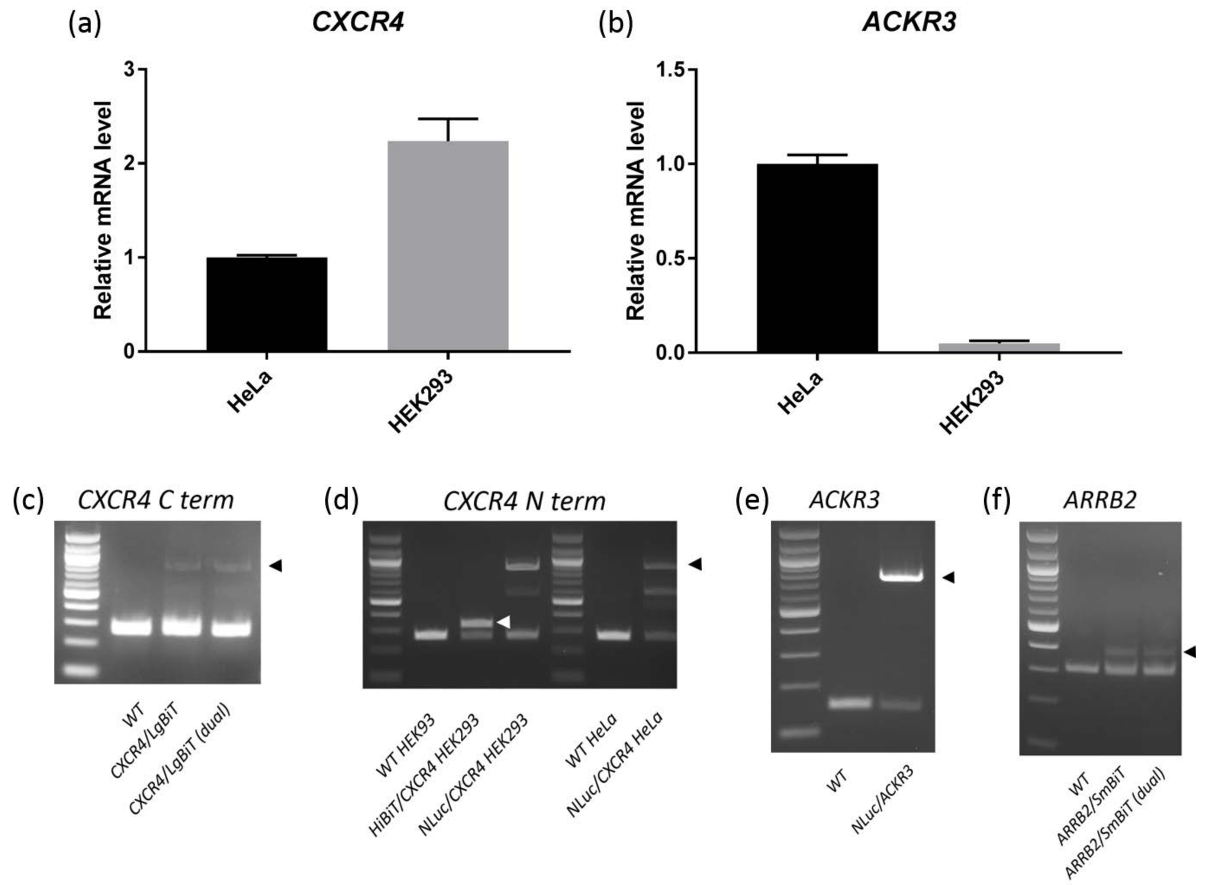

Supplementary Figure S1. (Related to Figure 1). Analysis of wildtype or genome edited cells. **(a)** CXCR4 mRNA expression in HeLa (black bar) or HEK293 (grey bar) cells and **(b)** ACKR3 mRNA expression in HeLa (black bar) or HEK293 (grey bar) cells. **(c-f)** Genome-editing resulted in clonal cell lines heterozygous for the insert. PCR amplification using target specific primers of DNA from: **(c)** wildtype (WT) HEK293 cells, CXCR4/LgBiT clones or cells edited to express both CXCR4/LgBiT and ARRB2/SmBiT (dual), (d) wildtype (WT) HEK293 cells, HiBiT/CXCR4 or Nluc/CXCR4 clones as well as WT HeLa cells and NLuc/CXCR4 HeLa clones, **(e)** wildtype (WT) HeLa cells and NLuc/ACKR3 clones and **(f)** wildtype (WT) HEK293 cells, ARRB2/SmBiT clones or clones edited to express both ARRB2/SmBiT and CXCR4/LgBiT (dual). Wildtype PCR product at 200-300bp. In arrows indicate PCR product of inserted tag. Bars represent mean ± s.e.m. of three experiments.

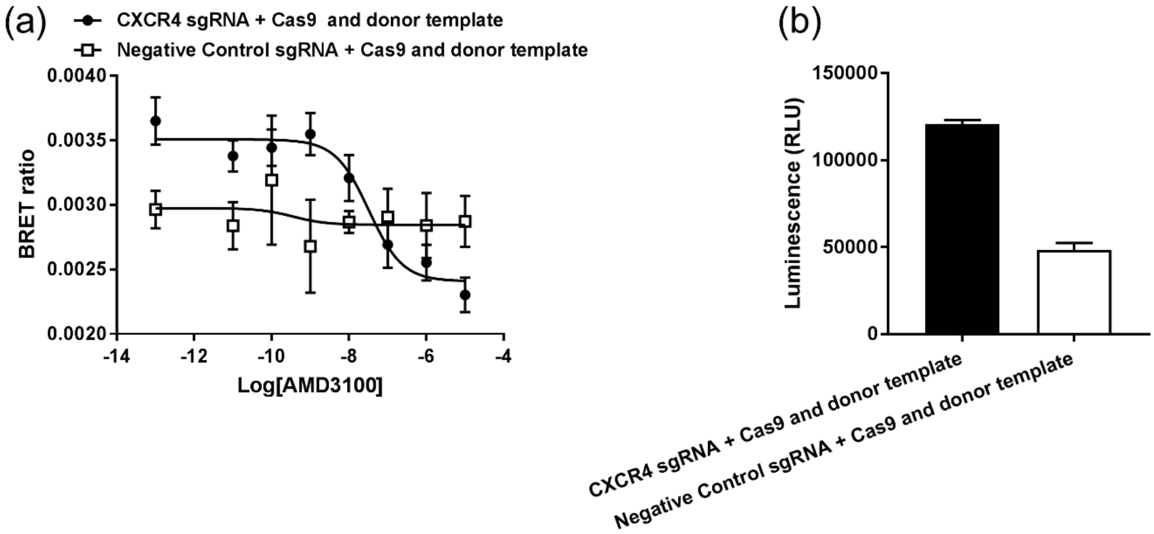

Supplementary Figure S2. (Related to Figure 2). NanoBRET competition ligand binding in a non-clonal pool of HEK293 cells some of which are expressing genome-edited Nluc/CXCR4 **(a)** Displacement of 12.5 nM CXCL12-AF47 binding by AMD3100 (10 pM – 10 µM) in non-clonal pool of cells transfected with plasmids encoding sgRNAs targeted to the CXCR4 N-terminus, Cas9 and NLuc/CXCR4 donor repair template (closed circles) or in non-clonal pool of cells transfected with plasmids encoding non-targeted sgRNAs, Cas9 and NLuc/CXCR4 donor repair template (open circles). **(b)** Luminescence generated from non-clonal pools of cells transfected either with plasmids encoding sgRNAs targeted to the CXCR4 N-terminus (closed bar) or untargeted sgRNA (open bar) in addition to Cas9 and NLuc/CXCR4 donor repair template. Bars or points represent mean ± s.e.m. of five individual experiments performed in triplicate.

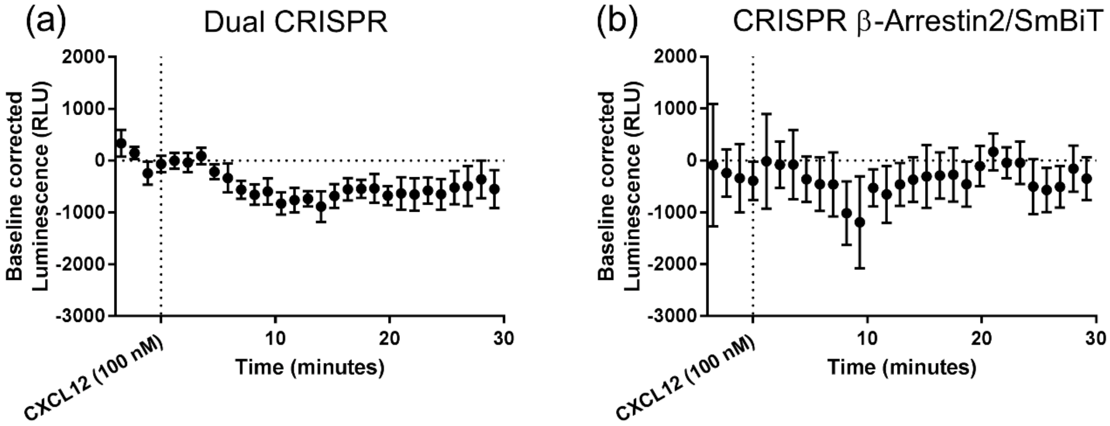

Supplementary Figure S3. (Related to Figure 4). Inhibition of CXCL12-mediated recruitment of genome-edited β-arrestin2/SmBiT recruitment to CXCR4/LgBiT by over-expression of β-arrestin2. HEK293 cells expressing **(a)** both genome-edited CXCR4/LgBiT and β-arrestin2/SmBiT (Dual CRISPR) or genome-edited β-arrestin2/SmBiT transiently transfected with CXCR4/LgBiT (CRISPR β-arrestin2/SmBiT) were transfected with 500 ng per well of a 6 well plate of β-arrestin2/Halotag (unlabelled). At time zero CXCL12 (100 nM) or HBSS was applied to the cells and luminescence changes in luminescence over the baseline was measured. Points represent mean ± s.e.m. of four **(a)** or three **(b)** individual experiments performed with replicates of 4-6. Baseline-corrected luminescence calculated as described in *Methods*.

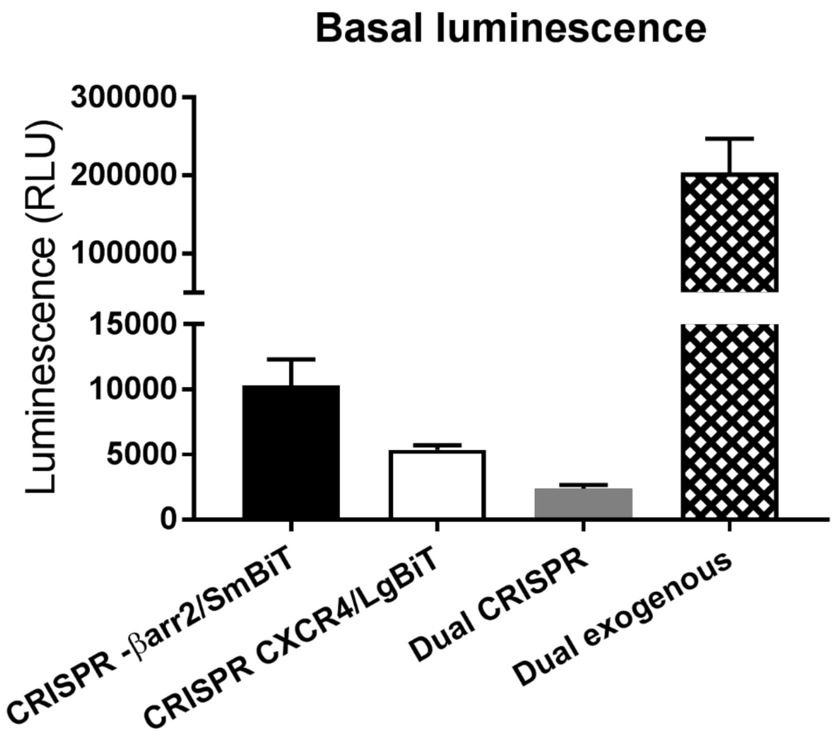

Supplementary Figure S4 (Related to Figure 4). Assessment of the effect of assay configuration on observations. Basal luminescence observed following application of furimazine (10 µM) in HEK293 cells expressing gene-edited β-arrestin2/SmBiT transiently transfected with CXCR4/LgBiT (CRISPR β-arr2/SmBiT, closed bar), genome-edited CXCR4/LgBiT transiently transfected with β-arrestin2/SmBiT (CRISPR CXCR4/LgBiT, open bar), both genome-edited CXCR4/LgBiT and gene-edited β-arrestin2/SmBiT (Dual CRISPR, grey bar), or HEK293 cells expressing transiently transfected CXCR4/LgBiT and β-arrestin2/SmBiT (Dual exogenous, hatched bar). Points or bars represent mean ± s.e.m. of six (CRISPR CXCR4/LgBiT and Dual exogenous), seven (CRISPR β-arrestin2/SmBiT) or eight (Dual CRISPR) individual experiments performed in triplicate.

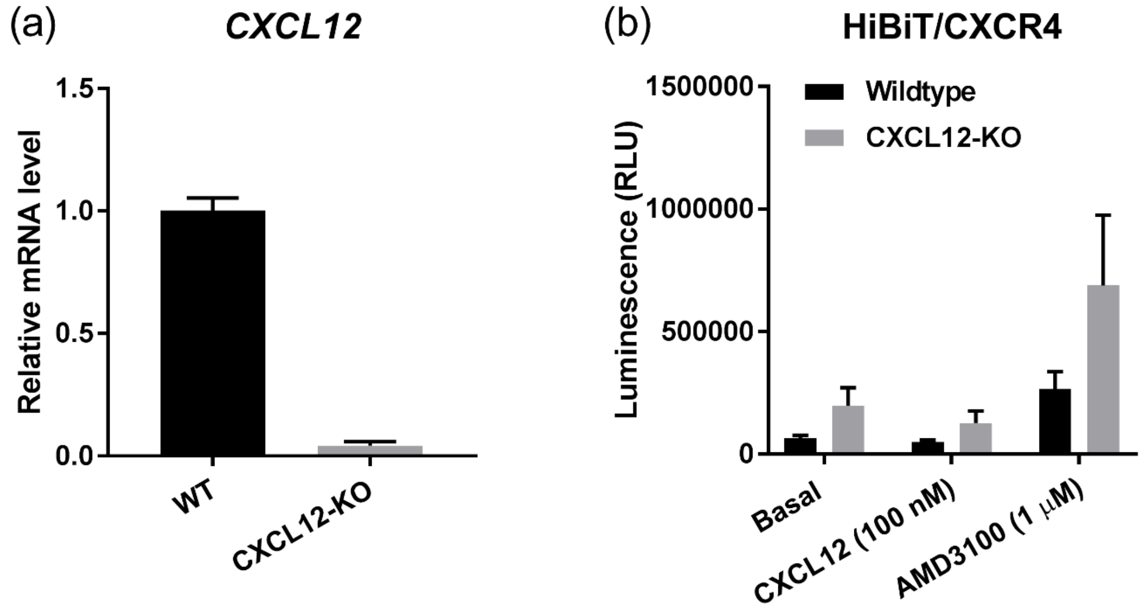

Supplementary Figure S5. (Related to Figure 5). (a) Analysis of *CXCL12* mRNA expression in wildtype (black bars) or genome-edited CXCL12 knockout HEK293 cells (grey bars). Bars represent mean ± S.D. of mRNA analysis from a single clone performed in triplicate. **(b) Determination of the effect of AMD3100 in the absence of endogenous CXCL12.** Change in luminescence in live wildtype HEK293 cells (black bar) or genome-edited CXCL12-KO HEK293 cells (grey bar) transiently transfected with HiBiT/CXCR4 in the absence (basal) or presence of CXCL12 (100 nM) or AMD3100 (1 µM). Luminescence generated by the addition of purified LgBiT (10 nM) and furimazine. Bars represent mean ± s.e.m. of four experiments performed in triplicate.

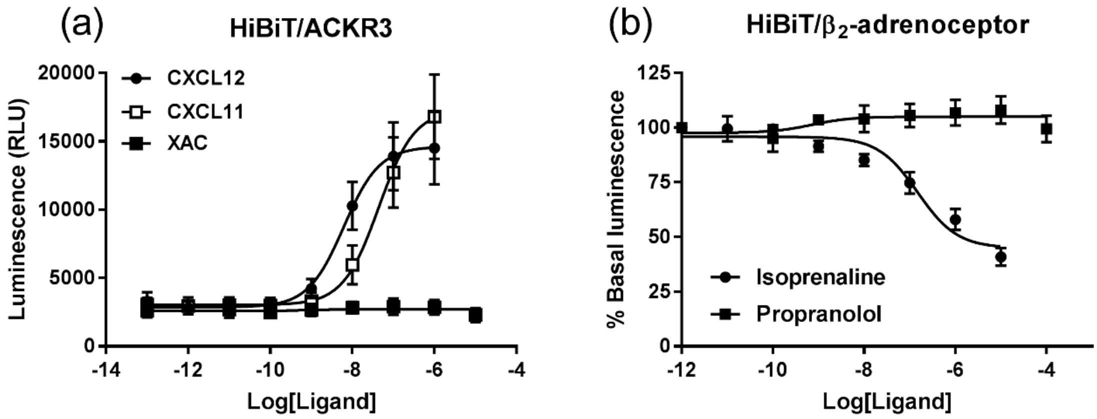

Supplementary Figure S6. (Related to Figure 5). Effect of ligand addition to HiBiT-tagged receptors. **(a)** HEK293 cells exogenously expressing HiBiT/ACKR3 were incubated in the absence or presence of increasing concentrations of CXCL12 (closed circles), CXCL11 (open squares) or XAC (closed squares). **(b)** HEK293 cells expressing genome-edited HiBiT/β_2-adrenoceptors were incubated in the absence or presence of increasing concentrations of isoprenaline (closed circles) or propranolol (closed squares). Ligands were incubated for 1 hour at 37 °C before luminescence was generated by the addition of furimazine (10 µM) and purified LgBiT (10 nM). Points represent mean ± s.e.m. luminescence or % baseline luminescence of five experiments performed in triplicate.

Supplementary Table 1: Primers used for in this study (Related to STAR Methods)

Primer[1]	Sequence	Target/Purpose
ACKR3N sgRNA1 fwd	CACCGATTGCCCGCCTCAGAACGA	N-terminally tagging ACKR3/ sgRNA construction
ACKR3N sgRNA1 rvs	AAACTCGTTCTGAGGCGGGCAATC	
ACKR3N sgRNA2 fwd	CACCGATGCAGATCCATCGTTCTG	
ACKR3N sgRNA2 rvs	AAACCAGAACGATGGATCTGCATC	
CXCR4N sgRNA1 fwd	CACCGATCCCCTCCATGGTAACCGC	N-terminally tagging CXCR4/ sgRNA construction
CXCR4N sgRNA1 rvs	AAACGCGGTTACCATGGAGGGGATC	
CXCR4N sgRNA2 fwd	CACCGTGGAGAACCAGCGGTTACCA	
CXCR4N sgRNA2 rvs	AAACTGGTAACCGCTGGTTCTCCAC	
CXCL12_KO_Sg1_Fwd	CACCGGCATGGGCATCTGTAGCTC	CXCL12 knockout/ sgRNA construction
CXCL12_KO_Sg1_Rvs	AAACGAGCTACAGATGCCCATGCC	
CXCL12_KO_Sg2_Fwd	CACCGCATCTGTAGCTCAGGCTGAC	
CXCL12_KO_Sg2_Rvs	AAACGTCAGCCTGAGCTACAGATGC	
ADRB2_sgRNA1_Fwd	CACCGCCTGCCAGACTGCGCGCCAT	N-terminally tagging ADRB2/ sgRNA construction
ADRB2_sgRNA1_Rvs	AAACATGGCGCGCAGTCTGGCAGG	
ADRB2_sgRNA2_Fwd	CACCGTTGCCCCATGGCGCGCAGTC	
ADRB2_sgRNA2_Rvs	AACGACTGCGCGCCATGGGGCAA	
ARRB2_Screen_Fwd	CCTCACCTCACAACCCTCTT	PCR genotype/ screen C-terminally tagged *ARRB2*
ARRB2_Screen_Rvs	CAAGAGAGGGTCCAGTGTGT	
CXCR4_Screen_C_Fwd	TCTCCAAAGGAAAGCGAGGT	PCR genotype/ screen C-terminally tagged *CXCR4*
CXCR4_Screen_C_Rvs	TCCTGCCTAGACACACATCA	
ACKR3HDRTSeq	TAGGTCATTTGATTGCCCGC	PCR genotype/ screen C-terminally tagged *ACKR3*
ACKR3_N_Screen_Rvs	CGCTTTTGTTGGGCATGTTG	
5`CXCR4NInt_Seq	TTTATAAAAGTCCGGCCGCG	PCR genotype/ screen N-terminally tagged *CXCR4*
3`CXCR4NInt_Seq	ACTCCTTTCGGTGACCCTTT	
Hu_CXCR4_Fwd	AACGTCAGTGAGGCAGATGA	*CXCR4* RTqPCR primer
Hu_CXCR4_Rvs	GCCAACCATGATGTGCTGAA	*CXCR4* RTqPCR primer
Hu_ACKR3_Fwd	CTCAGCACTAAGGGAGCCAG	*ACKR3* RTqPCR primer
Hu_ACKR3_Rvs	AGATCCATCGTTCTGAGGCG	*ACKR3* RTqPCR primer
huARRB2_RTqPCR_Fwd	TCCATGCTCCGTCACACTG	*ARRB2* RTqPCR primer
huARRB2_RTqPCR_Rev	ACAGAAGGCTCGAATCTCAAAG	*ARRB2* RTqPCR primer

Supplementary Table 2: Repair template sequences used in this study (Related to STAR Methods)

Target	Sequence
Tagging *CXCR4* on the N-terminus with NLuc	CAGAATTATGCCAAATCCTACCTTCTTCTGAAAGTATCTCCTAATTATCTGCACCTGACC<br>CTAGTGATGCTGTGAATGTGCAAGTATAGCTACATCCTCCGAAGGAAGGATCTTTACTC<br>CTTTTACCTCCTGAATGGGCTGCGTCTGCTGAAAGCGCGGGGAATGGCGTTGGAAGCTT<br>GGCCCTACTTCCAGCATTGCCGCCTACTGGTTGGGTTACTCCAGCAAGTCACTCCCCTTC<br>CCTGGGCCTCAGTGTCTCTACTGTAGCATTCCCAGGTCTGGAATTCCATCCACTTTAGCA<br>AGGATGGACGCGCCACAGAGAGACGCGTTCCTAGCCCGCGCTTCCCACCTGTCTTCAGG<br>CGCATCCCGCTTCCCTCAAACTTAGGAAATGCCTCTGGGAGGTCCTGTCCGGCTCCGGA<br>CTCACTACCGACCACCCGCAAACAGCAGGGTCCCCTGGGCTTCCCAAGCCGCGCACCTC<br>TCCGCCCCGCCCCTGCGCCCTCCTTCCTCGCGTCTGCCCCTCTCCCCCACCCCGCCTTCTC<br>CCTCCCCGCCCCAGCGGCGCATGCGCCGCGCTCGGAGCGTGTTTTTATAAAAGTCCGGC<br>CGCGGCCAGAAACTTCAGTTTGTTGGCTGCGGCAGCAGGTAGCAAAGTGACGCCGAGG<br>GCCTGAGTGCTCCAGTAGCCACCGCATCTGGAGAACCAGCGGTTACCATGAGGTTGTGT<br>ATTCCGCAAGTGTTGCTCGCTTTGTTCCTCTCTATGTTGACAGGTCCTGGGGAAGGCTCT<br>CGCAAGCTCCTGGTCTTCACACTCGAAGATTTCGTTGGGGACTGGCGACAGACAGCCGG<br>CTACAACCTGGACCAAGTCCTTGAACAGGGAGGTGTGTCCAGTTTGTTTCAGAATCTCG<br>GGGTGTCCGTAACTCCGATCCAAAGGATTGTCCTGAGCGGTGAAAATGGGCTGAAGATC<br>GACATCCATGTCATCATCCCGTATGAAGGTCTGAGCGGCGACCAAATGGGCCAGATCGA<br>AAAAATTTTTAAGGTGGTGTACCCTGTGGATGATCATCACTTTAAGGTGATCCTGCACT<br>ATGGCACACTGGTAATCGACGGGGTTACGCCGAACATGATCGACTATTTCGGACGGCCG<br>TATGAAGGCATCGCCGTGTTCGACGGCAAAAAGATCACTGTAACAGGGACCCTGTGGA<br>ACGGCAACAAAATTATCGACGAGCGCCTGATCAACCCCGACGGCTCCCTGCTGTTCCGA<br>GTAACCATCAACGGAGTGACCGGCTGGCGGCTGTGCGAACGCATTCTGGCGGGATCCCT<br>GGAGGGGATCAGTGTAAGTCCAGTTTCAACCTGCTTTGTCATAAATGTACAAACGTTTG<br>AACTTAGAGCGCAGCCCCTCTCCGAGCGGGCAGAAGCGGCCAGGACATTGGAGGTACC<br>CGTACTCCAAAAAAGGGTCACCGAAAGGAGTTTTCTTGACCATGCCTATATAGTGCGGG<br>TGGGTGGGGGGGGAGCAGGATTGGAATCTTTTTCTCTGTGAGTCGAGGAGAAACGACT<br>GGAAAGAGCGTTCCAGTGGCTGCATGTGTCTCCCCCTTGAGTCCCGCCGCGCGCGGCGG<br>CTTGCACGCTGTTTGCAAACGTAAGAACATTCTGTGCACAAGTGCAGAGAAGGCGTGCG<br>CGCTGCCTCGGGACTCAGACCACCGGTCTCTTCCTTGGGGAAGCGGGGATGTCTTGGAG<br>CGAGTTACATTGTCTGAATTTAGAGGCGGAGGGCGGCGTGCCTGGGCTGAGTTCCCAGG<br>AGGAGATTGCGCCCGCTTTAACTTCGGGGTTAAGCGCCTGGTGACTGTTCTTGACACTG<br>GGTGCGTGTTTGTTAAACTCTGTGCGGCCGACGGAGCTGTGCCAGTCTCCCAGCACAGT<br>AGGCAGAGGGCGGGAGAGGCGGGTGGACCCACCGCGCCGATCCTCTGAGGGGATCGAG<br>TGGTGGCAGCAGCTAGGAGTTGATCCGCCCGCGCGCTTTGGGTTTGAGGGGG
Tagging *CXCR4* on the N-terminus with HiBiT	CAGAATTATGCCAAATCCTACCTTCTTCTGAAAGTATCTCCTAATTATCTGCACCTGACC<br>CTAGTGATGCTGTGAATGTGCAAGTATAGCTACATCCTCCGAAGGAAGGATCTTTACTC<br>CTTTTACCTCCTGAATGGGCTGCGTCTGCTGAAAGCGCGGGGAATGGCGTTGGAAGCTT<br>GGCCCTACTTCCAGCATTGCCGCCTACTGGTTGGGTTACTCCAGCAAGTCACTCCCCTTC<br>CCTGGGCCTCAGTGTCTCTACTGTAGCATTCCCAGGTCTGGAATTCCATCCACTTTAGCA<br>AGGATGGACGCGCCACAGAGAGACGCGTTCCTAGCCCGCGCTTCCCACCTGTCTTCAGG<br>CGCATCCCGCTTCCCTCAAACTTAGGAAATGCCTCTGGGAGGTCCTGTCCGGCTCCGGA<br>CTCACTACCGACCACCCGCAAACAGCAGGGTCCCCTGGGCTTCCCAAGCCGCGCACCTC<br>TCCGCCCCGCCCCTGCGCCCTCCTTCCTCGCGTCTGCCCCTCTCCCCCACCCCGCCTTCTC<br>CCTCCCCGCCCCAGCGGCGCATGCGCCGCGCTCGGAGCGTGTTTTTATAAAAGTCCGGC<br>CGCGGCCAGAAACTTCAGTTTGTTGGCTGCGGCAGCAGGTAGCAAAGTGACGCCGAGG<br>GCCTGAGTGCTCCAGTAGCCACCGCATCTGGAGAACCAGCGGgTACCATGGTGAGCGGC<br>TGGCGGCTGTTCAAGAAGATTAGCGGGAGTTCTGGCGGCTCGAGCGGTGGATCCCTGGA<br>GGGGATCAGTGTAAGTCCAGTTTCAACCTGCTTTGTCATAAATGTACAAACGTTTGAAC<br>TTAGAGCGCAGCCCCTCTCCGAGCGGGCAGAAGCGGCCAGGACATTGGAGGTgCCCGTA<br>CTCCAAAAAAGGGTCACCGAAAGGAGTTTTCTTGACCATGCCTATATAGTGCGGGTGGG<br>TGGGGGGGGAGCAGGATTGGAATCTTTTTCTCTGTGAGTCGAGGAGAAACGACTGGAA<br>AGAGCGTTCCAGTGGCTGCATGTGTCTCCCCCTTGAGTCCCGCCGCGCGCGGCGGCTTG<br>CACGCTGTTTGCAAACGTAAGAACATTCTGTGCACAAGTGCAGAGAAGGCGTGCGCGCT<br>GCCTCGGGACTCAGACCACCGGTCTCTTCCTTGGGGAAGCGGGGATGTCTTGGAGCGAG<br>TTACATTGTCTGAATTTAGAGGCGGAGGGCGGCGTGCCTGGGCTGAGTTCCCAGGAGGA<br>GATTGCGCCCGCTTTAACTTCGGGGTTAAGCGCCTGGTGACTGTTCTTGACACTGGGTGC<br>GTGTTTGTTAAACTCTGTGCGGCCGACGGAGCTGTGCCAGTCTCCCAGCACAGTAGGCA<br>GAGGGCGGGAGAGGCGGGTGGACCCACCGCGCCGATCCTCTGAGGGGATCGAGTGGTG<br>GCAGCAGCTAGGAGTTGATCCGCCCGCGCGCTTTGGGTTTGAGGGGG

Tagging *ACKR3* on the N-terminus NLuc	AAAAACAGGTGCTGACCCAAAGCTTGAGACAGCTGGTTGGTTGCCCTTCGTTGAAAACC<br>ACACGGCCAACCCAGAGTCTTCAGGTTTGGGGCGCAGTTAAGCTGTGGGGCACCCTGCG<br>TAGGCTCACTGTGTTTCCAGAGGCTTTATGACTGCACACTGGGGCCGCCTGAGACCCAG<br>GTTTATATGTGAATTTAGCAGACAAGTAAAAAGTGCTGAGTCACATTATAAATGCAAAT<br>TTGGAGGAGGATTTGAAATGCTTTCATTAAGACACCAAAGCAGGACACTTGTAATACAG<br>CAGTGCCAGGAGAGGACAACCCTGCCATTCTTTGGGGACCTAGATAAAATGGCTCCACT<br>GTCCCCTGCTGAGAGTGAGCAGGCCATCAGCATCGCGGTGGCCGGGGCTCAGCTGCCTG<br>ACGTTTCTTTGGCCCCTGTACTGTGTTGATTTCACACACTGGGCACAGCCATGCTGGCGG<br>TGTCTTGAGAACACAGCCATGAAAAGATGGAGAAAGCAAACACAGTTTCTGTCTTGGA<br>GCACCACATGGATTTAGATTCAGAGTATGATTTATTTTGTTTTGGAGAGTTTTATTATGA<br>ATTTTTTTTCTCCCTTCTTTGCAAAGACATTACAGAGCTAAACCCAAACAGCTGAGCCTA<br>TCAGGGCCTTGGAAAAGCATTTTTGCCTGAAACTTGAGTTTTTCCTAATGAAGTGACTTT<br>CCTCTTCCATCTTTTTTGTTTGCTTGGTTTTCTCATAGGTCATTTGATTGCCCGCCTCAGA<br>ACGGGTACCATGCGGCTCTGCATCCCGCAGGTGCTGTTGGCCTTGTTCCTTTCCATGCTG<br>ACAGGGCCGGGAGAAGGCAGCCGGAAGCTTCTGGTCTTCACACTCGAAGATTTCGTTGG<br>GGACTGGCGACAGACAGCCGGCTACAACCTGGACCAAGTCCTTGAACAGGGAGGTGTG<br>TCCAGTTTGTTTCAGAATCTCGGGGTGTCCGTAACTCCGATCCAAAGGATTGTCCTGAGC<br>GGTGAAAATGGGCTGAAGATCGACATCCATGTCATCATCCCGTATGAAGGTCTGAGCGG<br>CGACCAAATGGGCCAGATCGAAAAAATTTTTAAGGTGGTGTACCCTGTGGATGATCATC<br>ACTTTAAGGTGATCCTGCACTATGGCACACTGGTAATCGACGGGGTTACGCCGAACATG<br>ATCGACTATTTCGGACGGCCGTATGAAGGCATCGCCGTGTTCGACGGCAAAAAGATCAC<br>TGTAACAGGGACCCTGTGGAACGGCAACAAAATTATCGACGAGCGCCTGATCAACCCC<br>GACGGCTCCCTGCTGTTCCGAGTAACCATCAACGGAGTGACCGGCTGGCGGCTGTGCGA<br>ACGCATTCTGGCGGGATCCGATCTGCATCTCTTCGACTACTCAGAGCCAGGGAACTTCT<br>CGGACATCAGCTGGCCATGCAACAGCAGCGACTGCATCGTGGTGGACACGGTGATGTG<br>TCCCAACATGCCCAACAAAAGCGTCCTGCTCTACACGCTCTCCTTCATTTACATTTTCAT<br>CTTCGTCATCGGCATGATTGCCAACTCCGTGGTGGTCTGGGTGAATATCCAGGCCAAGA<br>CCACAGGCTATGACACGCACTGCTACATCTTGAACCTGGCCATTGCCGACCTGTGGGTT<br>GTCCTCACCATCCCAGTCTGGGTGGTCAGTCTCGTGCAGCACAACCAGTGGCCCATGGG<br>CGAGCTCACGTGCAAAGTCACACACCTCATCTTCTCCATCAACCTCTTCGGCAGCATTTT<br>CTTCCTCACGTGCATGAGCGTGGACCGCTACCTCTCCATCACCTACTTCACCAACACCCC<br>CAGCAGCAGGAAGAAGATGGTACGCCGTGTCGTCTGCATCCTGGTGTGGCTGCTGGCCT<br>TCTGCGTGTCTCTGCCTGACACCTACTACCTGAAGACCGTCACGTCTGCGTCCAACAATG<br>AGACCTACTGCCGGTCCTTCTACCCCGAGCACAGCATCAAGGAGTGGCTGATCGGCATG<br>GAGCTGGTCTCCGTTGTCTTGGGCTTTGCCGTTCCCTTCTCCATTATCGCTGTCTTCTACT<br>TCCTGCTGGCCAGAGCCATCTCGGCGTCCAGTGACCAGGAGAAGCACAGCAGCCGGAA<br>GATCATCTTCTCCTACGTGGTGG
Tagging *ADRB2* on the N-terminus with HiBiT (Synthesised as a ssODN)	GGCCCGCAGAGCCCCGCCGTGGGTCCGCCCGCTGAGGCGCCCCCAGCCAGTGCGCTCAC<br>CTGCCAGACTGCGCGCCATGGTGAGCGGCTGGCGGCTGTTCAAGAAGATTAGCGGGAG<br>TTCTGGCGGGCAACCCGGGAACGGCAGCGCCTTCTTGCTGGCACCCAATGGAAGCCATG<br>CGCCGGACCACGACGTCACGCAGG
Tagging *CXCR4* on the C-terminus with LgBiT (Repair template described in White et al, 2017)	TCTTTGTCATCACGCTTCCCTTCTGGGCAGTTGATGCCGTGGCAAACTGGTACTTTGGGA<br>ACTTCCTATGCAAGGCAGTCCATGTCATCTACACAGTCAACCTCTACAGCAGTGTCCTC<br>ATCCTGGCCTTCATCAGTCTGGACCGCTACCTGGCCATCGTCCACGCCACCAACAGTCA<br>GAGGCCAAGGAAGCTGTTGGCTGAAAAGGTGGTCTATGTTGGCGTCTGGATCCCTGCCC<br>TCCTGCTGACTATTCCCGACTTCATCTTTGCCAACGTCAGTGAGGCAGATGACAGATAT<br>ATCTGTGACCGCTTCTACCCCAATGACTTGTGGGTGGTTGTGTTCCAGTTTCAGCACATC<br>ATGGTTGGCCTTATCCTGCCTGGTATTGTCATCCTGTCCTGCTATTGCATTATCATCTCCA<br>AGCTGTCACACTCCAAGGGCCACCAGAAGCGCAAGGCCCTCAAGACCACAGTCATCCT<br>CATCCTGGCTTTCTTCGCCTGTTGGCTGCCTTACTACATTGGGATCAGCATCGACTCCTT<br>CATCCTCCTGGAAATCATCAAGCAAGGGTGTGAGTTTGAGAACACTGTGCACAAGTGGA<br>TTTCCATCACCGAGGCCCTAGCTTTCTTCCACTGTTGTCTGAACCCCATCCTCTATGCTTT<br>CCTTGGAGCCAAATTTAAAACCTCTGCCCAGCACGCACTCACCTCTGTGAGCAGAGGGT<br>CCAGCCTCAAGATCCTCTCCAAAGGAAAGCGAGGTGGACATTCATCTGTTTCGACTGAG<br>TCTGAGTCTTCAAGTTTTCACTCCAGCGCGGGACTCGAGAATTCTGGCTCCAGCGGTGG<br>TGGCGGGAGCGGAGGTGGAGGGTCGTCAGGTGTCTTCACACTCGAAGATTTCGTTGGGG<br>ACTGGGAACAGACAGCCGCCTACAACCTGGACCAAGTCCTTGAACAGGGAGGTGTGTC<br>CAGTTTGCTGCAGAATCTCGCCGTGTCCGTAACTCCGATCCAAAGGATTGTCCGGAGCG<br>GTGAAAATGCCCTGAAGATCGACATCCATGTCATCATCCCGTATGAAGGTCTGAGCGCC<br>GACCAAATGGCCCAGATCGAAGAGGTGTTTAAGGTGGTGTACCCTGTGGATGATCATCA<br>CTTTAAGGTGATCCTGCCCTATGGCACACTGGTAATCGACGGGGTTACGCCGAACATGC<br>TGAACTATTTCGGACGGCCGTATGAAGGCATCGCCGTGTTCGACGGCAAAAAGATCACT<br>GTAACAGGGACCCTGTGGAACGGCAACAAAATTATCGACGAGCGCCTGATCACCCCCG<br>ACGGCTGCATGCTGTTCCGAGTAACCATCAACAGCTAATCTAGAGGGCCCCACAGATGT<br>AAAAGACTTTTTTTATACGATAAATAACTTTTTTTTAAGTTACACATTTTTCAGATATA

	AAAGACTGACCAATATTGTACAGTTTTTATTGCTTGTTGGATTTTTGTCTTGTGTTTCTTT<br>AGTTTTTGTGAAGTTTAATTGACTTATTTATATAAATTTTTTTTGTTTCATATTGATGTGT<br>GTCTAGGCAGGACCTGTGGCCAAGTTCTTAGTTGCTGTATGTCTCGTGGTAGGACTGTA<br>GAAAAGGGAACTGAACATTCCAGAGCGTGTAGTGAATCACGTAAAGCTAGAAATGATC<br>CCCAGCTGTTTATGCATAGATAATCTCTCCATTCCCGTGGAACGTTTTTCCTGTTCTTAA<br>GACGTGATTTTGCTGTAGAAGATGGCACTTATAACCAAAGCCCAAAGTGGTATAGAAAT<br>GCTGGTTTTTCAGTTTTCAGGAGTGGGTTGATTTCAGCACCTACAGTGTACAGTCTTGTA<br>TTAAGTTGTTAATAAAAGTACATGTTAAACTTACTTAGTGTTATGTTCTGATTTCTGTTG<br>ACATTCTTTTGGCTAGTAGAAGACAAAAGTAATACATTTATGGTATGCAAAGCACTATC<br>CTAGGTATTTCATTGTAATATTTTACTTACCCCTTATCACAACTCTGATAGATTCTGCTTC<br>TGTTACTAATTACATTTTATAGAAGAGGAAACGGAGGCACAGAAAGCCTAAGTAACTTG<br>GTTAAAGGCATGTAGTAAGTATCAAATCCTGTATTTTAAACCAGGTAACATGACTTAAC<br>GAATCTGAAGCCTTC
Tagging *ARRB2* on the C-terminus with SmBiT	GAGGCGGGTGAGTGTCATGGGGGAGCCTGGGTGGGGGTCACACTGGCTCTCTCTAGTCC<br>CATGTCGTCGTCCTCTTCACGATGCCTCTCCCCTTCCCCAGGGATGTCTCTGTGGAGCTG<br>CCTTTTGTTCTTATGCACCCCAAGCCCCACGACCACATCCCCCTCCCCAGACCCCAGTCA<br>GGTGAGCACACTACCCACCCCAAGCCCTCAGAGGGAGGGCCTGAAGCAGGGCCAGTGG<br>AGGAAAACTGGCCCTTCCAGCACCCACCCCCACACCCCCTCTTCCCGTCCCCCCAGCCG<br>CTCCGGAGACAGATGTCCCTGTGGACACCAACCTCATTGAATTTGATACCAAGTAAGAA<br>ACTCATTCCCCTACTTGACCCTCTTGGGACAAAGATTCCTATAACATTCAAATCTGCCCT<br>CATACCTCTTCCTTGCTTTTGGTGGGGAGAAGCGGATTGTAGCATCAAATCAAGATGCC<br>TTAGCCTTGTGAGGCTGCCTCTTGCTGCCTTTTCTTTGTCCCTTCCTGTAAATACCTCTGG<br>TCCCACTGCTGTTCGAACGCCTCTGTCCCAGAGGCCTAGCTTCGGGGAGGGCAGGGAGT<br>GGGAGGCTGGGACAAGAGTCAGAAGCCCTCACCTCACAACCCTCTTTCCCACCACCAAG<br>CTATGCCACAGATGATGACATTGTGTTTGAGGACTTTGCCCGGCTTCGGCTGAAGGGGA<br>TGAAGGATGACGACTATGATGATCAACTCTGCCTCGAGGGTGGTGGCGGGAGCGGAGG<br>TGGAGGGTCGTCAGGTGTGACCGGCTACCGGCTGTTCGAGGAGATTCTGTAATCTAGAG<br>AAGCGGGGTGGGAAGAAGGGAGGGGATGGGGTTGGGAGAGGTGAGGGCAGGATTAAG<br>ATCCCCACTGTCAATGGGGGATTGTCCCAGCCCCTCTTCCCTTCCCCTCACCTGGAAGCT<br>TCTTCAACCAATCCCTTCACACTCTCTCCCCCATCCCCCCAAGATACACACTGGACCCTC<br>TCTTGCTGAATGTGGGCATTAATTTTTTGACTGCAGCTCTGCTTCTCCAGCCCCGCCGTG<br>GGTGGCAAGCTGTGTTCATACCTAAATTTTCTGGAAGGGGACAGTGAAAAGAGGAGTG<br>ACAGGAGGGAAAGGGGGAGACAAAACTCCTACTCTCAACCTCACACCAACACCTCCCA<br>TTATCACTCTCTCTGCCCCCATTCCTTCAAGAGGAGACCCTTTGGGGACAAGGCCGTTTC<br>TTTGTTTCTGAGCATAAAGAAGAAAATAAATCTTTTACTAAGCATGAGTGTGTGTTTTCT<br>CTGTAGTGTTTAGAGAGTGTATGGTGTGCTTATCCATGATTCTGTTAGCTTGTGGGGGAG<br>GACCCAGTCTCTCTTCTGCACCCAAAAGCCACCTGACACGGAGGCTATCAGCAAATGTT<br>TATTGGATGACTGTGCTGTGGCTGTGTTCCAGGGTAAACATCAAAACTTGACTTAGTCC<br>AAACTCAAGGGTGAGGCTTGATAAGGCCACAAAGTGAACTTAGTCATT
