# Supplementary material for: CRISPR-Mediated Protein Tagging with Nanoluciferase to Investigate Native Chemokine Receptor Function and Conformational Changes
Source: Cell Chem Biol. 2020 May 21;27(5):499–510.e7. doi: 10.1016/j.chembiol.2020.01.010 (PMC7242902; doi:10.1016/j.chembiol.2020.01.010)
Supplement: Document S2. Article plus Supplemental Information [file mmc2.pdf]

Article

Cell Chemical Biology

CRISPR-Mediated Protein Tagging with Nanoluciferase to Investigate Native Chemokine Receptor Function and Conformational Changes

Graphical Abstract

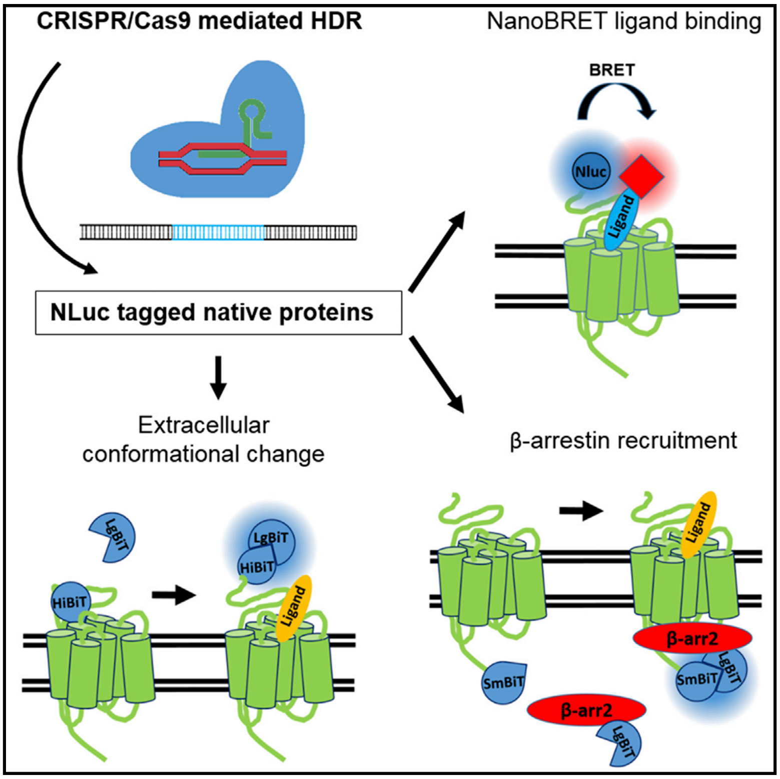

Highlights

- Utilizing CRISPR/Cas9-mediated knockin to investigate GPCR function
- NanoBRET ligand binding at CRISPR/Cas9 genome-edited chemokine receptors
- Real time monitoring of live cell interactions between native CXCR4 and β-arrestin2
- Development of an approach to examine extracellular CXCR4 conformational changes

Authors

Carl W. White, Birgit Caspar, Hannah K. Vanyai, Kevin D.G. Pfleger, Stephen J. Hill

Correspondence

carl.white@perkins.uwa.edu.au (C.W.W.),
stephen.hill@nottingham.ac.uk (S.J.H.)

In Brief

White et al. demonstrate the utility of combining bioluminescence resonance energy transfer (BRET) and CRISPR/Cas9 genome editing to investigate the function of G protein-coupled receptors that are natively expressed. They also demonstrate that nanoluciferase complementation can be used to investigate receptor conformational changes.

White et al., 2020, Cell Chemical Biology *27*, 499–510
May 21, 2020 © 2020 The Author(s). Published by Elsevier Ltd.
https://doi.org/10.1016/j.chembiol.2020.01.010

CellPress

Cell Chemical Biology

CellPress
OPEN ACCESS

Article

CRISPR-Mediated Protein Tagging with Nanoluciferase to Investigate Native Chemokine Receptor Function and Conformational Changes

Carl W. White,[1,2,3,4,*] Birgit Caspar,[1,2] Hannah K. Vanyai,[5] Kevin D.G. Pfleger,[3,4,6] and Stephen J. Hill[1,2,3,7,*]
[1]Cell Signalling and Pharmacology Research Group, Division of Physiology, Pharmacology & Neuroscience, School of Life Sciences, University of Nottingham, Queens Medical Centre, Nottingham NG7 2UH, UK
[2]Centre of Membrane Proteins and Receptors, University of Birmingham and University of Nottingham, The Midlands, UK
[3]Harry Perkins Institute of Medical Research and Centre for Medical Research, The University of Western Australia, QEII Medical Centre, Nedlands, WA 6009, Australia
[4]Australian Research Council Centre for Personalised Therapeutics Technologies, Australia
[5]Epithelial Biology Laboratory, The Francis Crick Institute, 1 Midland Road, London NW1 1AT, UK
[6]Dimerix Limited, Nedlands, WA 6009, Australia
[7]Lead Contact
*Correspondence: carl.white@perkins.uwa.edu.au (C.W.W.), stephen.hill@nottingham.ac.uk (S.J.H.)
https://doi.org/10.1016/j.chembiol.2020.01.010
Parts of this work have been published in abstract form as part of the conference proceedings for Experimental Biology 2019 (White and Hill, 2019).

SUMMARY

G protein-coupled receptors are a major class of membrane receptors that mediate physiological and pathophysiological cellular signaling. Many aspects of receptor activation and signaling can be investigated using genetically encoded luminescent fusion proteins. However, the use of these biosensors in live cell systems requires the exogenous expression of the tagged protein of interest. To maintain the normal cellular context here we use CRISPR/Cas9-mediated homology-directed repair to insert luminescent tags into the endogenous genome. Using NanoLuc and bioluminescence resonance energy transfer we demonstrate fluorescent ligand binding at genome-edited chemokine receptors. We also demonstrate that split-NanoLuc complementation can be used to investigate conformational changes and internalization of CXCR4 and that recruitment of β-arrestin2 to CXCR4 can be monitored when both proteins are natively expressed. These results show that genetically encoded luminescent biosensors can be used to investigate numerous aspects of receptor function at native expression levels.

INTRODUCTION

G protein-coupled receptors (GPCRs) are a major class of membrane receptors that control numerous physiological responses via ligand-mediated signal transduction. The response elicited by a given GPCR is dependent on the cellular context, i.e., the cellular proteome and a cascade of factors including receptor compartmentalization (Ellisdon and Halls, 2016; Tsvetanova et al., 2015), association with interacting proteins (Bockaert et al., 2004), binding of a specific ligand and subsequent conformational rearrangement resulting in activation (Wang et al., 2018), coupling to specific intercellular effectors (e.g., G proteins) (Wang et al., 2018; Rankovic et al., 2016), or scaffolding proteins (e.g., GPCR kinases and arrestins) (Walther and Ferguson, 2015), as well as internalization, trafficking, and recycling of the receptor (Magalhaes et al., 2012). Many of these processes can be studied using genetically encoded luminescent and/or fluorescent fusion proteins that allow for investigation of receptor or protein function by sensitive microscopic or biophysical techniques such as resonance energy transfer. Indeed, luciferase-based assays have been developed to investigate GPCR-ligand binding (Stoddart et al., 2015), G protein activation, and protein-protein interactions (Lohse et al., 2012), as well as receptor internalization and trafficking (Lan et al., 2012; Tiulpakov et al., 2016) by monitoring changes in bioluminescence resonance energy transfer (BRET) or luciferase complementation. However, the use of these biosensors in cellular systems is typically accomplished by exogenous expression of the tagged protein(s) of interest that can perturb the normal cellular context and stoichiometry of the cellular interactome, particularly where the level of exogenous expression is high.

To overcome the need for exogenous expression of a luciferase-tagged protein of interest in BRET assays, we and others have used CRISPR/Cas9 genome engineering to insert the 19-kDa nanoluciferase (NanoLuc, NLuc) into endogenous mammalian loci via homology-directed recombination (White et al., 2017, 2019; Oh-Hashi et al., 2016). This results in NLuc fusion proteins being expressed under endogenous promotion and has been used to investigate ligand binding to adenosine A_{2B} receptors (White et al., 2019), as well as CXCR4 receptor trafficking and β-arrestin2 recruitment to GPCRs (White et al., 2017) by monitoring changes in resonance energy transfer between the NLuc luminescent donor and a fluorescent acceptor. In addition, reports have

This is an open access article under the CC BY license (http://creativecommons.org/licenses/by/4.0/).

also demonstrated the use of CRISPR/Cas9 genome editing to insert small self-complementing fragments of NLuc into the endogenous genome (Oh-Hashi et al., 2017; Schwinn et al., 2018). This approach allowed for quantification of protein expression by changes in luminescence following luciferase complementation (Oh-Hashi et al., 2017; Schwinn et al., 2018) as well as post-translational modifications of endogenous proteins to be investigated by NanoBRET (with addition of an exogenous fluorescent probe) (Schwinn et al., 2018). The split NLuc system (NanoBiT) comprises a small 11-amino acid peptide engineered to interact with an 18-kDa polypeptide of NLuc (LgBiT) with either high (~700 pM) or low (~190 μM) affinity (Dixon et al., 2016). These two high- and low-affinity systems can therefore be configured to investigate either transient or stable protein interactions. Indeed, using exogenously expressed proteins, multiple studies now report monitoring GPCR-protein interactions and receptor internalization, as well as changes in protein expression, with the NanoBiT system (Reyes-Alcaraz et al., 2018; Dixon et al., 2016; Laschet et al., 2019; Storme et al., 2018).

Despite these advances in the NanoBRET and nanoluciferase complementation techniques, an outstanding limitation is that the investigation of protein-protein interactions still requires exogenous expression of protein tagged with a fluorescent acceptor. Tagging endogenous proteins with a fluorescent protein is readily achievable (Kamiyama et al., 2016), while the sensitivity of nanoluciferase complementation should be sufficient to detect interactions between two genome-edited proteins. However, to our knowledge no studies have reported using NanoBRET or NanoBiT complementation to investigate interactions between two proteins expressed under endogenous promotion. Furthermore, these genome-edited nanoluciferase techniques have, so far, only been established on a few receptors and assay configurations.

Using CRISPR/Cas9, here we aimed to further apply and develop genome-edited NLuc/NanoBiT-based assays that can be used to investigate GPCR function with proteins expressed under endogenous promotion. We demonstrate that multiple aspects of chemokine receptor signaling can be investigated using these genome-edited NanoBRET/NanoBiT techniques including quantification of endogenous receptor expression and ligand binding as well as receptor conformational changes and internalization. We also established that ligand-mediated recruitment of β-arrestin2 to CXCR4 can be observed when both proteins are endogenously expressed. Finally, these approaches allowed for the comparison of responses mediated by exogenous and genome-edited proteins and therefore discussion of the associated caveats.

RESULTS

Genome Engineering

Here we used CRISPR/Cas9-mediated homology-directed repair to successfully generate genome-edited HEK293 cells expressing CXCR4 tagged on the N terminus with NLuc or the modified 11-amino acid high-affinity NLuc fragment ($K_d \sim$ 700 pM, HiBiT [Dixon et al., 2016]) yielding NLuc/CXCR4 and HiBiT/CXCR4, respectively. We also generated HEK293 cells expressing genome-edited β-arrestin2 (also known as arrestin-3) tagged on the C terminus with the modified low-affinity 11-amino acid NLuc fragment ($K_d \sim$ 190 μM, SmBiT [Dixon et al., 2016]; β-arrestin2/SmBiT) as well as HeLa cells expressing genome-edited NLuc/CXCR4 or ACKR3 tagged on the N terminus with NLuc (NLuc/ACKR3). In agreement with a lack of detectable *ACKR3* mRNA in HEK293 cells (Thul et al., 2017) (Figure S1), no clones expressing NLuc/ACKR3 could be generated. All cells lines tested were heterozygous for the insert (Figures S1C–S1F) as is typical of non-diploid cell lines such as triploidic to tetraploidic HEK293 cells (Stepanenko and Dmitrenko, 2015), which results in homozygous knockin being a rare occurrence. Analysis of *CXCR4* and *ARRB2* (genes encoding CXCR4 and β-arrestin2) mRNA levels following CRISPR/Cas9-mediated tagging showed significant variation in *CXCR4* expression between HEK293 or HeLa cell lines (Figures 1A and 1B; $p < 0.01$); however, no significant differences in *ARRB2* expression in HEK293 cells were observed (Figure 1C). Bioluminescence imaging of cells expressing genome-edited NLuc/CXCR4 (Figures 1D and 1E) showed localization at the plasma membrane and intracellular compartments in both HEK293 and HeLa cells, whereas when complemented with the purified and cell-impermeant-modified 18-kDa fragment of NLuc (LgBiT), exclusive membrane localization was observed for cells expressing genome-edited HiBiT/CXCR4 in HEK293 cells (Figure 1F). In agreement with reported intracellular localization of ACKR3 (Rajagopal et al., 2010), NLuc/ACKR3 expression was primarily observed clustered in a perinuclear region in genome-edited HeLa cells (Figure 1G).

NanoBRET Ligand Binding at CXCR4 and ACKR3 Chemokine Receptors

Previously we used NanoBRET to investigate ligand binding to exogenously expressed GPCRs (Stoddart et al., 2015), receptor tyrosine kinases (Kilpatrick et al., 2017), and more recently ligand binding to adenosine A_{2B} receptors expressed under endogenous promotion (White et al., 2019). Here, we have further expanded on these approaches and demonstrate fluorescent ligand binding at genome-edited NLuc/CXCR4 (Figure 2; HEK293 and HeLa cells) and NLuc/ACKR3 (Figure 3; HeLa cells) chemokine receptors. Initial studies confirmed our previous reports (Caspar et al., 2018) of clear saturable specific binding of CXCL12-AF647 to membranes from HEK293 cells stably expressing exogenous NLuc/CXCR4 (Figure 2A; $pK_d = 7.55 \pm 0.06$, $n = 3$). In addition, we demonstrated CXCL12-AF647 binding to exogenous NLuc/ACKR3 stably expressed in HEK293 cells (Figure 3A; $pK_d = 8.12 \pm 0.10$, $n = 5$) as well as membranes (Figure 3B; $pK_d = 8.83 \pm 0.06$, $n = 4$). Exemplifying the high assay sensitivity of NanoBRET ligand binding, clear saturable ligand binding was achieved at the low levels of expression found in all clonal genome-edited cell lines (Figures 2 and 3). Similarly, AMD3100 competition with CXCL12-AF647 for binding to genome-edited NLuc/CXCR4 receptors was able to be detected in a non-clonal pool of HEK293 cells, estimated <5% positive, transiently transfected with Cas9 guides and NLuc/CXCR4 repair templates (Figure S2; $pIC_{50} = 7.56 \pm 0.22$, $n = 5$).

The level of receptor expression and/or oligomerization has the potential to modulate aspects of receptor function. Indeed, CXCR4 and ACKR3 are capable of forming oligomeric complexes that modulate signaling (Decaillot et al., 2011) and GPCR oligomer formation can lead to negative cooperativity between protomers (May et al., 2011; Sohy et al., 2009). To investigate possible effects of receptor expression level or

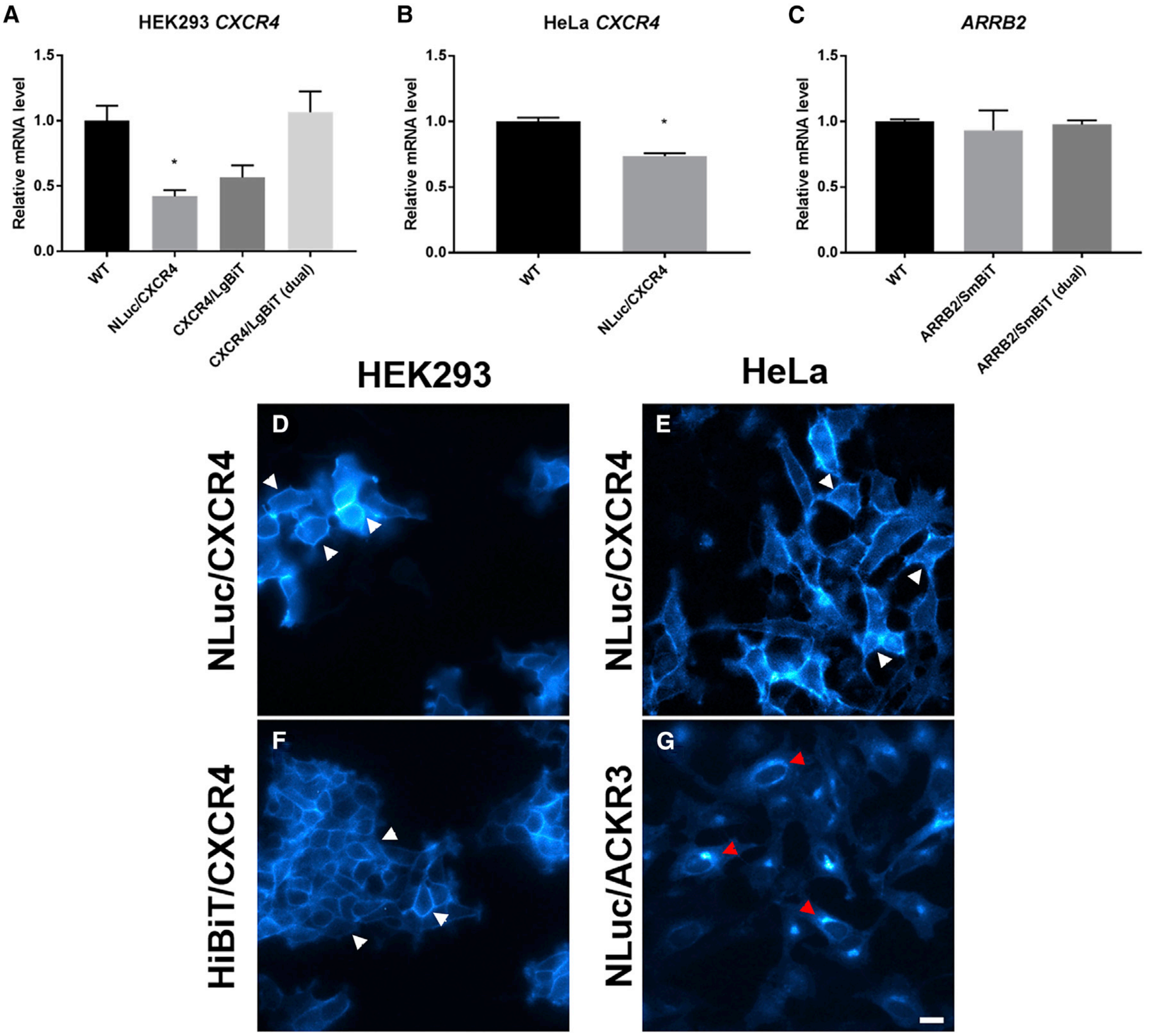

Figure 1. Analysis of Protein Expression Following Genome Editing

(A) *CXCR4* mRNA expression in wild-type HEK293 cells or HEK293 clones expressing genome-edited NLuc/CXCR4, CXCR4/LgBiT, or CXCR4/LgBiT and ARRB2/SmBiT (dual).

(B) *CXCR4* mRNA expression in wild-type HeLa cells or HeLa clones expressing genome-edited NLuc/CXCR4.

(C) *ARRB2* mRNA expression in wild-type HEK293 cells or HEK293 clones expressing genome-edited ARRB2/SmBiT, or ARRB2/SmBiT and CXCR4/LgBiT (dual). Relative mRNA level, normalized to BM2 expression. Bars represent mean ± SEM of three cell passages of a single clone performed in triplicate.

(D–G) Visualization of genome-edited receptor localization in HEK293 and HeLa cells using a bioluminescence LV200 Olympus microscope. (D) HEK293 and (E) HeLa cells expressing genome-edited NLuc/CXCR4, (F) HEK293 cells expressing genome-edited HiBiT/CXCR4 complemented with LgBiT and (G) HeLa cells expressing genome-edited NLuc/ACKR3. White arrow heads (D–F) indicate predominant expression at the plasma membrane of luciferase-tagged CXCR4, red arrow heads (G) indicate NLuc/ACKR3 expression in cytosolic compartments. Images were acquired by capturing total luminescence for 90 s.
Scale bar represents 20 μm. See Figure S1.

oligomerization on ligand binding, we took advantage of the differences in expression of our genetically engineered and exogenous cells lines. Quantification of NLuc-tagged receptor expression (Figure 2E) showed ~75- and ~60-fold greater NLuc/CXCR4 expression in the exogenous cell lines than genome-edited HEK293 or HeLa cells, respectively. Similarly, exogenous expression of NLuc/ACKR3 in stable HEK293 cells, which lack endogenous *ACKR3* (Figure S1B), was ~400-fold greater than that seen in genome-edited HeLa cells (Figure 3D). However, we observed no difference in the binding affinity of CXCL12-AF647 to NLuc/CXCR4 expressed in genome-edited HEK293 cells with low levels of expression (Figure 2B; pK_d = 7.50 ± 0.04, n = 3) or in genome-edited HeLa cells (Figure 2C; pK_d = 7.58 ± 0.04, n = 4), where ACKR3 is also endogenously expressed, compared with HEK293 cell membranes expressing exogenous NLuc/CXCR4 (Figure 2A). Likewise, we only observed small differences in the affinities (Table 1) of CXCL12-AF647 binding to NLuc/ACKR3 when expressed exogenously in live HEK293 cells (Figure 3A; pK_d = 8.12 ± 0.10, n = 5) or membranes (Figure 3B; pK_d = 8.83 ± 0.06, n = 4) or expressed in genome-edited HeLa cells (Figure 3C; pK_d = 8.77 ± 0.11, n = 6). This small difference in affinity may be related to the ability

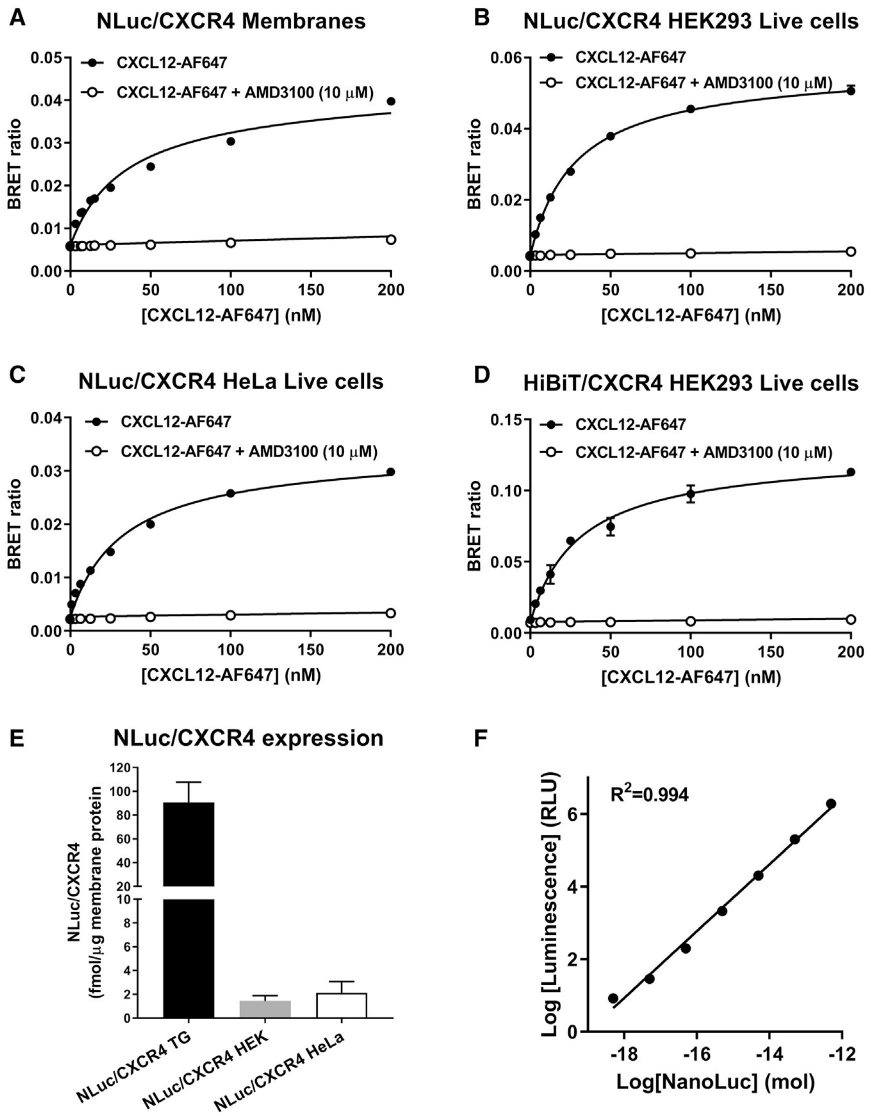

Figure 2. Determination of the Binding Affinity of CXCL12-AF647 at NLuc/CXCR4

(A–D) NanoBRET saturation ligand binding curves obtained in (A) membrane preparations from HEK293 cells exogenously expressing NLuc/CXCR4 (B) live HEK293 cells expressing genome-edited NLuc/CXCR4 (C) live HeLa cells expressing genome-edited NLuc/CXCR4 or (D) live HEK293 cells expressing genome-edited HiBiT/CXCR4 complemented with LgBiT. Cells or membranes were incubated with increasing concentrations of CXCL12-AF647 in the absence (black circles) or presence (white circles) of AMD3100 (10 μM) for 1 h at 37°C. Data shown are mean ± SEM and are representative of three or four independent experiments performed in duplicate for (A and B) and (C and D), respectively.

(E) Quantification of NLuc/CXCR4 expression by linear regression (F), as described in the STAR Methods, using membrane preparations made from HEK293 cells exogenously expressing NLuc/CXCR4 (NLuc/CXCR4 TG, black bar), HEK293 cells expressing genome-edited NLuc/CXCR4 (NLuc/CXCR4 HEK, gray bar), or HeLa cells expressing genome-edited NLuc/CXCR4 (NLuc/CXCR4 HeLa, white bar).

Data shown are (F) mean ± SEM or (E) representative of five individual experiments performed in triplicate (see Figure S2).

of ACKR3 to scavenge and internalize CXCL12 but requires further investigation.

Finally, the use of fluorescent agonists in live cell NanoBRET ligand binding assays can result in internalization of the receptor. To localize binding to receptors at the plasma membrane in a live cell assay, we used HEK293 cells expressing genome-edited HiBiT/CXCR4 with functional NLuc generated following ligand equilibration by complementation of the HiBiT-tagged receptor with exogenously added cell-impermeant LgBiT. This limited luminescence (Figure 1F) and therefore observable NanoBRET signal to receptors remaining at the plasma membrane. However, no difference in the binding affinity of CXCL12-AF647 to HiBiT/CXCR4 compared with NLuc/CXCR4 (Figure 2D; pK_d = 7.49 ± 0.05, n = 4) was observed.

Measurement of CXCR4 Recruitment of β-Arrestin2 by NanoBiT Complementation

Following ligand binding, GPCRs interact with a number of intracellular proteins that modulate as well as elicit their function. Among these, β-arrestin scaffolding proteins have been extensively studied and regulate GPCR internalization as well as intracellular signaling. However, methods used to investigate live cell β-arrestin recruitment to a GPCR, and protein-protein interactions in general, largely require the use of a reporter protein that is exogenously overexpressed. We have previously investigated receptor-β-arrestin2 interactions using BRET where one protein fused to NLuc was expressed under endogenous promotion; however, we could not observe interactions between two endogenously expressed proteins. Here, we sought to determine if endogenous CXCR4-β-arrestin2 interactions could be investigated using genome-edited proteins and the low-affinity NanoBiT (SmBiT-LgBiT, K_d ~ 190 μM) complementation system. In HEK293 cells expressing genome-edited β-arrestin2/SmBiT transiently transfected with CXCR4/LgBiT (Figures 4A and 4B), HEK293 cells expressing genome-edited CXCR4/LgBiT transiently transfected with β-arrestin2/SmBiT (Figures 4C and 4D), HEK293 cells expressing both genome-edited CXCR4/LgBiT and β-arrestin2/SmBiT (Figures 4E and 4F) and HEK293 cells expressing transiently transfected CXCR4/LgBiT and β-arrestin2/SmBiT (Figures 4G and 4H), we observed an increase in luminescence following CXCL12 (300 nM) addition that was inhibited by AMD3100 (1 μM). Recruitment of genome-edited β-arrestin2/SmBiT to genome-edited or transiently expressed CXCR4/LgBiT could be inhibited by overexpression of unlabeled β-arrestin2/Halotag (Figure S3), indicative of a specific protein-protein interaction. Basal luminescence varied between

CellPress
OPEN ACCESS

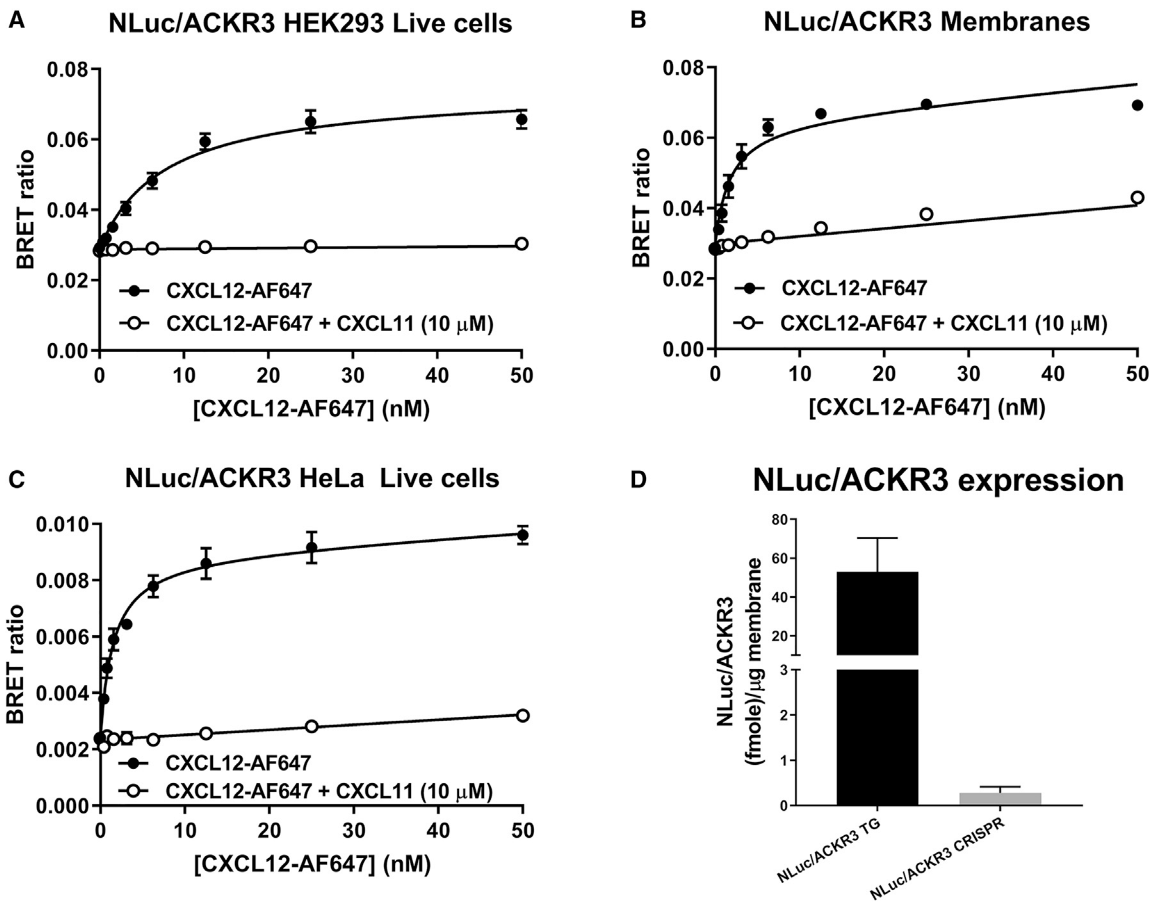

Figure 3. Determination of the Binding Affinity of CXCL12-AF647 at NLuc/ACKR3

(A–C) NanoBRET saturation ligand binding curves obtained in (A) live HEK293 cells exogenously expressing NLuc/ACKR3, (B) membrane preparations from HEK293 cells exogenously expressing NLuc/ACKR3, and (C) live HeLa cells expressing genome-edited NLuc/ACKR3. Cells or membranes were incubated with increasing concentrations of CXCL12-AF647 in the absence (black circles) or presence (white circles) of CXCL11 (10 μM) for 1 h at 37°C. Data shown are mean ± SEM and are representative of five (A), four (B), and six (C) experiments performed in duplicate.

(D) Quantification of NLuc/ACKR3 expression by linear regression, as described in the STAR Methods, using membrane preparations made from HEK293 cells exogenously expressing NLuc/ACKR3 (NLuc/ACKR3 TG, black bar) or HeLa cells expressing genome-edited NLuc/ACKR3 (NLuc/ACKR3 CRISPR, gray bar). Data shown are mean ± SEM of five individual experiments performed in triplicate.

assay configuration indicative of differences in expression level and constitutive β-arrestin2/SmBiT recruitment to CXCR4/LgBiT dependent on the relative levels of receptor and effector expression (Figure S4A). As expected, the greatest basal luminescence in genome-edited HEK293 cells was observed when CXCR4/LgBiT was in excess of β-arrestin2/SmBiT (Figure S4A) where constitutive CXCR4 activity would be highest. Similarly, in HEK293 cells expressing genome-edited CXCR4/LgBiT transiently transfected with β-arrestin2/SmBiT, where there was an excess of β-arrestin2 relative to receptor, analysis of the kinetic profile of recruitment showed faster recruitment ($t_{1/2}$, time in minutes to half maximum response ± SEM: 1.89 ± 0.17 min, n = 6) compared with HEK293 cells expressing genome-edited β-arrestin2/SmBiT and transiently expressed CXCR4/LgBiT ($t_{1/2}$: 4.62 ± 0.39 min, $p < 0.05$, n = 7), HEK293 cells expressing both genome-edited CXCR4/LgBiT and genome-edited β-arrestin2/SmBiT ($t_{1/2}$: 3.11 ± 0.12 min, n = 8) or HEK293 cells transiently expressing CXCR4/LgBiT and β-arrestin2/SmBiT ($t_{1/2}$: 5.27 ± 0.52 min, $p < 0.01$, n = 7). In contrast to the other assay configurations, cells expressing genome-edited CXCR4/LgBiT and transiently expressed β-arrestin2/SmBiT showed a unique transient recruitment profile (Figure 4C). CXCL12 induced a concentration-dependent increase in luminescence in all assay configurations with similar potency when measured approximately 5 min after ligand addition (Figures 4B, 4D, 4F and, 4H: pEC_{50} = 7.48 ± 0.04, 7.69 ± 0.14, 7.16 ± 0.17, and 7.66 ± 0.23, n = 6–8, respectively).

Using HiBiT-Tagged Receptors to Investigate Cell Surface Expression and CXCR4 Conformational Changes

Following agonist-induced receptor activation and β-arrestin recruitment, CXCR4, like many GPCRs, is internalized and trafficked via endosomes to lysosomes for degradation or recycled back to the plasma membrane (Magalhaes et al., 2012). Because of the cell-impermeant nature of purified LgBiT, ligand-induced changes in cell surface receptor expression and/or internalization should be able to be monitored by measuring the extent of

Table 1. Binding Affinities of CXCL12-AF647 at NLuc/CXCR4 or NLuc/ACKR3 Measured by NanoBRET

Cell Line or Membrane Preparation	pK_d	n	Tagged Receptor Expression Level (fmol/μg membrane)[b]
TG NLuc/CXCR4	(7.15 ± 0.04)[a]	–	–
TG NLuc/CXCR4 membranes	7.55 ± 0.06 (7.61 ± 0.10)[a]	3	90.67 ± 17.06
CRISPR NLuc/ CXCR4 HEK	7.50 ± 0.04	3	1.45 ± 0.43
CRISPR NLuc/ CXCR4 HeLa	7.58 ± 0.04	4	2.12 ± 0.95
CRISPR HiBiT/ CXCR4	7.49 ± 0.05	4	ND
TG NLuc/ACKR3 HEK***	8.12 ± 0.10	5	–
TG NLuc/ACKR3 membranes	8.83 ± 0.06	4	52.9 ± 17.4
CRISPR NLuc/ ACKR3 HeLa	8.77 ± 0.11	6	0.27 ± 0.14

TG cell lines indicate cells expressing transgenic exogenously expressed receptors, CRISPR cells lines indicate those expressing a genome-edited receptor under endogenous promotion. TG NLuc/CXCR4 or TG NLuc/ ACKR3 membranes made from the respective cell lines. ***$p < 0.001$ was determined by a one-way ANOVA and indicate a significant difference between assay configurations in the binding affinity of CXCL12-AF647 to NLuc/ACKR3.

[a]pK_d values in parentheses from Caspar et al. (2018).

[b]Values indicate mean ± SEM of five independent experiments performed in triplicate.

luminescence following addition of exogenous LgBiT to cells (to determine the level of cell surface receptors) after incubation for different times with agonist. This assay has been described previously for internalization of Galanin (Reyes-Alcaraz et al., 2018) and Orexin (Rouault et al., 2017) receptors, but to our knowledge has not been applied to investigate CXCR4 internalization. We initially established the assay using HEK293 cells stably overexpressing HiBiT/CXCR4 (Figure 5A) and in live cells observed a concentration-dependent decrease in luminescence following application of CXCL12 (pEC_{50} = 8.69 ± 0.06, n = 5), consistent with a decrease in cell surface expression and internalization. Surprisingly inhibitors of CXCR4, AMD3100 (pEC_{50} = 6.99 ± 0.22, n = 5) and IT1t (pEC_{50} = 7.55 ± 0.11, n = 5), but not the adenosine receptor antagonist XAC (xanthine amine congener), resulted in a concentration-dependent increase in luminescence suggestive of an increase in cell surface expression, potentially due to constitutive trafficking to the membrane. Taking advantage of our genome-edited HiBiT/CXCR4 HEK293 cells (Figure 5B) we confirmed the increase in luminescence mediated by AMD3100 (pEC_{50} = 6.91 ± 0.14, n = 5) and IT1t (pEC_{50} = 7.46 ± 0.07, n = 5) was not a consequence of receptor overexpression. Furthermore, CXCL12 (pEC_{50} = 8.46 ± 0.20, n = 5)-mediated receptor internalization has been observed at HiBiT/CXCR4 expressed under endogenous promotion confirming the assay could be used at receptors expressed under endogenous promotion.

It has previously been shown that CXCR4 function can be modulated by constitutive receptor internalization and trafficking. Indeed, inhibition of endocytosis increases CXCR4 cell surface expression in a manner independent of CXCL12 (Pelekanos et al., 2014), whereas CXCL12 changes the constitutive dynamics of CXCR4 at the plasma membrane causing receptor immobilization and/or accumulation in lipid rafts that enhances signaling (Wysoczynski et al., 2005). Furthermore, it has been proposed that increasing constitutive CXCR4 cell surface expression may be a useful strategy to enhance migration of systemically transplanted cells (Pelekanos et al., 2014). To establish if the model could be used to better understand the effects of constitutive CXCR4 trafficking, we first sought to confirm that constitutive trafficking was indeed being observed. In CXCL12 knockout HEK293 cells (CXCL12-KO, Figure S5A) transiently transfected with HiBiT/CXCR4, application of AMD3100 produced an increase in luminescence (Figure S5B), indicating that the effect was not driven by endogenous CXCL12. However, in HEK293 cells expressing genome-edited HiBiT/CXCR4 (Figure 5C), kinetic analysis showed that AMD3100 and IT1t mediated a rapid, but saturable, increase in luminescence suggestive of a non-active-trafficking process. To further rule out active forward receptor trafficking or receptor internalization being involved in the change in luminescence we used saponin-permeabilized membrane preparations from HEK293 cells exogenously expressing HiBiT/CXCR4 (Figures 5D and 5E). Here, despite an absence of receptor trafficking we observed a similar concentration-dependent saturable increase in luminescence mediated by AMD3100 (pEC_{50} = 6.98 ± 0.39, n = 5) and IT1t (pEC_{50} = 8.38 ± 0.02, n = 5). Moreover, in contrast to live cells, CXCL12 resulted in a small increase in luminescence in membrane preparations but inhibited the increase in luminescence mediated by AMD3100 (1 μM, Figure 5F; $p < 0.01$ for 100 nM and 1 μM CXCL12), therefore, indicating that the ligand-mediated effects were due to specific changes in CXCR4. However, together, these data were not supportive of the assay configuration simply reporting on antagonist-mediated changes in constitutive CXCR4 trafficking in live cells.

It is known that small-molecule inhibitors such as AMD3100 induce conformational rearrangement of the extracellular domains of CXCR4 that can result in modulation of monoclonal antibody binding, despite themselves binding within the transmembrane bundle (Carnec et al., 2005; Rosenkilde et al., 2004). Therefore, we hypothesized that under basal conditions the extracellular conformation of CXCR4 resulted in steric hindrance and that application of AMD3100 or IT1t resulted in a conformation more favourable for HiBiT-LgBiT complementation. In genome-edited HEK293 cells expressing HiBiT/CXCR4, the affinity of complementation with purified LgBiT (Figure 6A; K_d = 229.8 ± 37.2 nM) was lower than that observed in genome-edited HEK293 cells expressing β_2-adrenoceptors tagged on the N-terminal with HiBiT (HiBiT/β_2-adrenceptor) (Figure 6D; K_d = 54.5 ± 14.6 nM, $p < 0.05$, one-way ANOVA with Dunnett's multiple comparisons test). Furthermore, AMD3100 (10 μM) resulted in an increase in the affinity of HiBiT/CXCR4-LgBiT complementation in cells (Figure 6A; K_d = 58.5 ± 9.6 nM, n = 7, $p < 0.01$) and membranes (Figure 6B; K_d = 180 ± 16.1 nM and 115.1 ± 9.5 nM in the absence and presence of AMD3100, respectively, n = 6, $p < 0.01$) but not for complementation of purified HiBiT to purified LgBiT

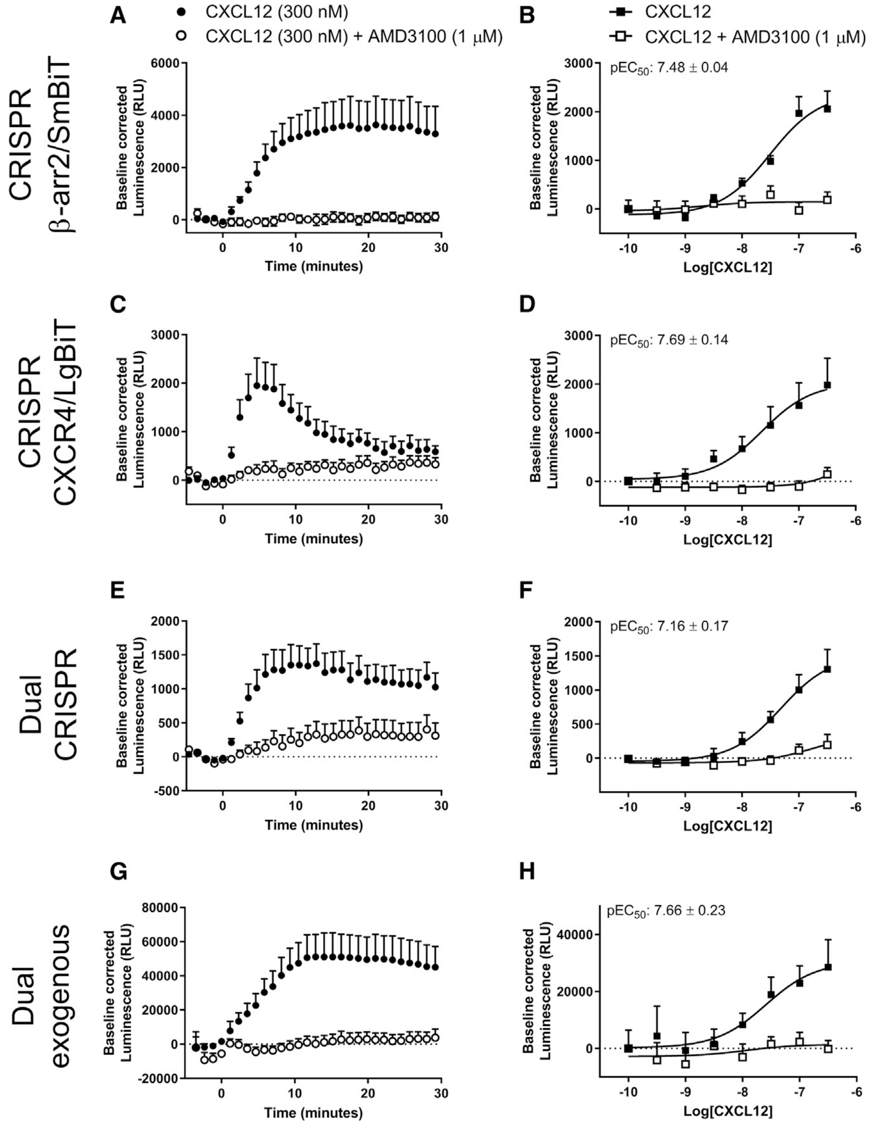

Figure 4. Investigation of β-Arrestin2/SmBiT Recruitment to CXCR4/LgBiT in Genome-Edited HEK293 Cells

HEK293 cells expressing (A and B) genome-edited β-arrestin2/SmBiT transiently transfected with CXCR4/LgBiT (CRISPR β-arr2/SmBiT), (C and D) genome-edited CXCR4/LgBiT transiently transfected with β-arrestin2/SmBiT (CRISPR CXCR4/LgBiT), (E and F) both genome-edited CXCR4/LgBiT and genome-edited β-arrestin2/SmBiT (Dual CRISPR), or (G and H) HEK293 cells expressing transiently transfected CXCR4/LgBiT and β-arrestin2/SmBiT (dual exogenous), were stimulated with 300 nM CXCL12 (A, C, E, and G) or increasing concentrations of CXCL12 (0.3–300 nM) (B, D, F, and H) in the absence (black squares and circles) or presence (white squares and circles) of 1 μM AM3100. Points represent mean ± SEM of six (C and D), seven (A, B, E, and F) or eight (G and H) individual experiments performed in triplicate. pEC_{50} values stated were calculated from response at approximately 5 min after ligand addition. Baseline-corrected luminescence calculated as described in the STAR Methods (see Figures S3 and S4).

(Figure 6C; K_d = 6.99 ± 0.45 nM and 7.73 ± 0.84 nM, n = 5, in the absence and presence of AMD3100, respectively). To further investigate if these effects were specific to HiBiT/CXCR4, we used HEK293 cells expressing exogenous HiBiT/ACKR3 (Figure S6A) and observed a concentration-dependent increase in luminescence following application of CXCL11 or CXCL12 (pEC_{50} = 7.48 ± 0.11 and pEC_{50} = 8.23 ± 0.05, n = 5, respectively). In HEK293 cells expressing genome-edited HiBiT/β_2-adrenoceptors (Figure S6B), application of isoprenaline (pEC_{50} = 6.82 ± 0.31, n = 5) but not propranolol resulted in a concentration-dependent decrease in luminescence indicative of internalization. These results demonstrate that for some receptors nanoluciferase complementation assays can be configured to investigate ligand-induced conformational changes.

DISCUSSION

Using CRISPR/Cas9-mediated homology-directed repair, we have expanded the use of genome-edited NanoBRET and NanoBiT techniques to investigate multiple aspects of GPCR function. However, analysis of mRNA expression following engineering showed a variable change in *CXCR4* expression in some clonal lines. We have reported changes in expression following genome editing that depends on the tag sequence (Khan et al., 2019); however, here this appears unlikely since the two HEK293 cell lines expressing genome-edited CXCR4/LgBiT did not show comparable effects, and analysis of *ARRB2* expression showed no observable differences following editing. An alternative explanation is that changes in expression are due to on/off-target effects of the editing (Zhang et al., 2015), or due to the subsequent cloning procedure resulting in amplification of a founder cell with acquired changes to the cellular proteome. Indeed, such compensatory rewiring has been described by other groups in clonal knockout cells engineered with CRISPR/Cas9 (Luttrell et al., 2018). Furthermore, all of the genome-edited clones that we generated were heterozygous for the insert, therefore differences in expression also accounts for changes in untagged alleles. Although we were only able to perform these analyses on a single clonal cell line from each configuration, these data suggest that changes in expression are specific to the individual genome-edited clonal line. Despite this, the changes in expression in genome-edited cells are relatively minor compared with the level of overexpression that occurs when receptors are expressed exogenously.

Determining the parameters of ligand-receptor binding, i.e., ligand affinity for a receptor, underpins the pharmacological understanding of receptor function. NanoBRET ligand binding is a homogeneous assay capable of investigating ligand binding at

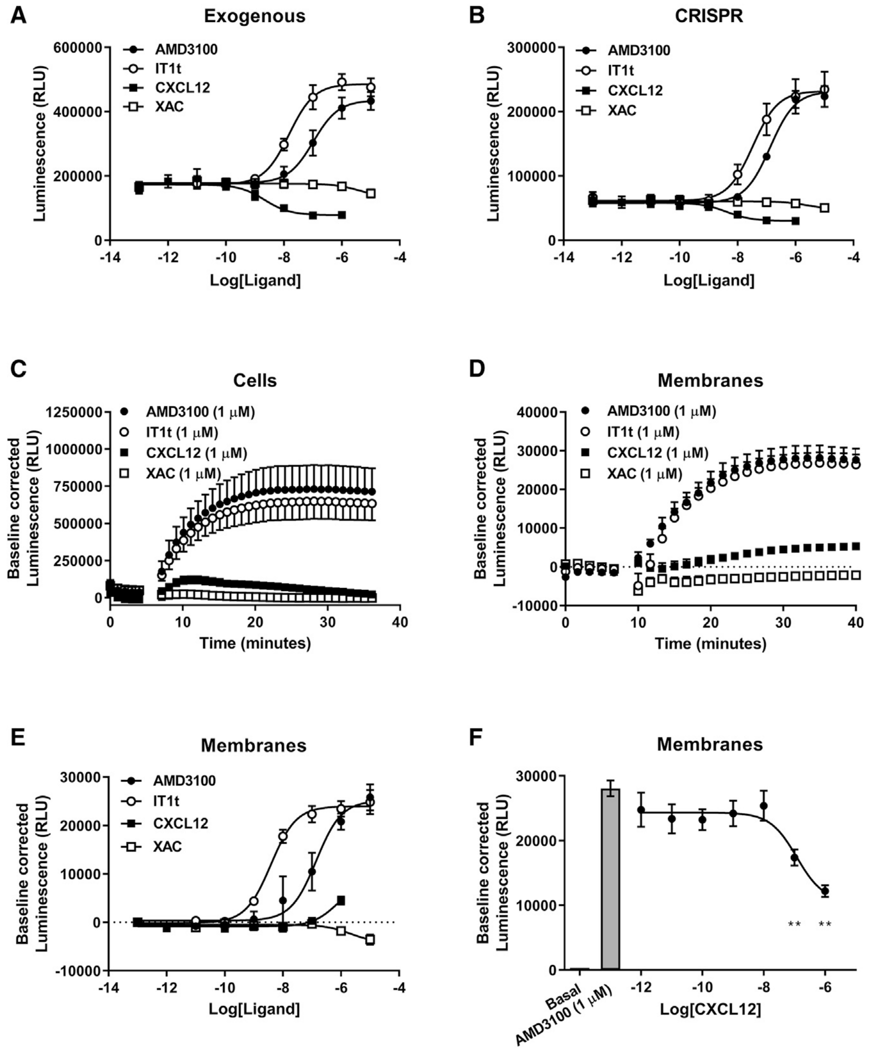

Figure 5. Using HiBiT-tagged CXCR4 to Investigate Cell Surface Expression and/or Conformational Changes

(A–D) HEK293 cells expressing (A) exogenous or (B) genome-edited HiBiT/CXCR4 were incubated in the absence or presence of increasing concentrations of AMD3100 (black circles), IT1t (white circles), CXCL12 (black squares), or XAC (white squares) for 1 h at 37°C and luminescence measured 30 min following addition of purified LgBiT (10 nM) and furimazine (10 μM). Kinetic analysis of the change in luminescence mediated by addition of AMD3100 (1 μM, black circles), IT1t (1 μM, white circles), CXCL12 (1 μM, black squares), or XAC (1 μM, white squares) in (C) HEK293 cells expressing genome-edited HiBiT/CXCR4 or (D) using membrane preparations from HEK293 cells exogenously expressing HiBiT/CXCR4, both pre-incubated with 10 nM purified LgBiT.

(E) AMD3100 (black circles), IT1t (white circles), CXCL12 (black squares), or XAC (white squares) concentration-response curves using membrane preparations from HEK293 cells exogenously expressing HiBiT/CXCR4 complemented with LgBiT.

(F) Concentration-dependent inhibition of the AMD3100-mediated increase in luminescence by CXCL12 using membrane preparations from HEK293 cells exogenously expressing HiBiT/CXCR4 complemented with LgbiT and pre-incubated with 10 nM purified LgBiT. Points represent mean ± SEM of four (D), five (A, B, and, E), six (C), or eight (F) experiments performed in triplicate. Baseline-corrected luminescence calculated as described in the STAR Methods. **$p < 0.01$. Statistical analysis by one-way ANOVA with a Dunnett's multiple comparisons test (see Figures S5 and S6).

both exogenous and genome-edited receptors (Stoddart et al., 2015; White et al., 2019). Here, we further demonstrate the sensitivity of NanoBRET to investigate fluorescent ligand binding at both exogenous and genome-edited chemokine receptors across a range of expression levels and on a non-clonal pool of cells. This latter approach is analogous to a plasmid-based transient transfection and is complementary to that described previously for NanoBiT tagging using purified Cas9 and single-stranded oligo DNA nucleotides in that it provides a rapid method for generating assayable genome-edited cells (Schwinn et al., 2018). Since no clonal isolation is required, any effects on the cellular phenotype is limited and the approach may be a useful strategy for editing primary cells with finite population doubling times.

Because of the ratiometric nature of BRET, the number of binding sites, and therefore protein expression levels, cannot be directly determined from NanoBRET saturation binding assays. However, we demonstrate that NLuc enzymatic activity, reported to be linear over eight orders of magnitude (Schwinn et al., 2018), can be used to quantify luciferase-tagged receptor expression and therefore may be a useful supplement to NanoBRET binding assays. However, untagged receptors in heterozygous genome-edited cell lines or endogenous receptors in exogenously expressed cell lines are not detected. Similarly, quantification of NLuc-tagged receptor expression in live cell systems would detect receptors potentially inaccessible to some ligands such as those found in intracellular compartments.

The function of CXCR4 can be influenced by spatiotemporal factors such as cellular compartmentalization (Wysoczynski et al., 2005) as well as by the absence, presence, or relative stoichiometry of interacting partners found in the cellular proteome (Heuninck et al., 2019). Here, we observed no difference in the binding affinity of CXCL12-AF647 for NLuc/CXCR4, despite the differences in NanoBRET ligand binding assays used, i.e., high or low expression of CXCR4, the absence or presence of ACKR3, or where the effects of agonist-induced internalization of CXCR4 were removed, indicating that the variables we tested have little impact on CXCL12 binding to CXCR4. Indeed, while CXCR4 is thought to form homo/hetero-dimers with CCR2, CCR5, CXCR3, and ACKR3, modulation of ligand binding by cooperative and/or allosteric interactions has primarily been reported for CCR2, CCR5, and CXCR3 (Sohy et al., 2009; Watts

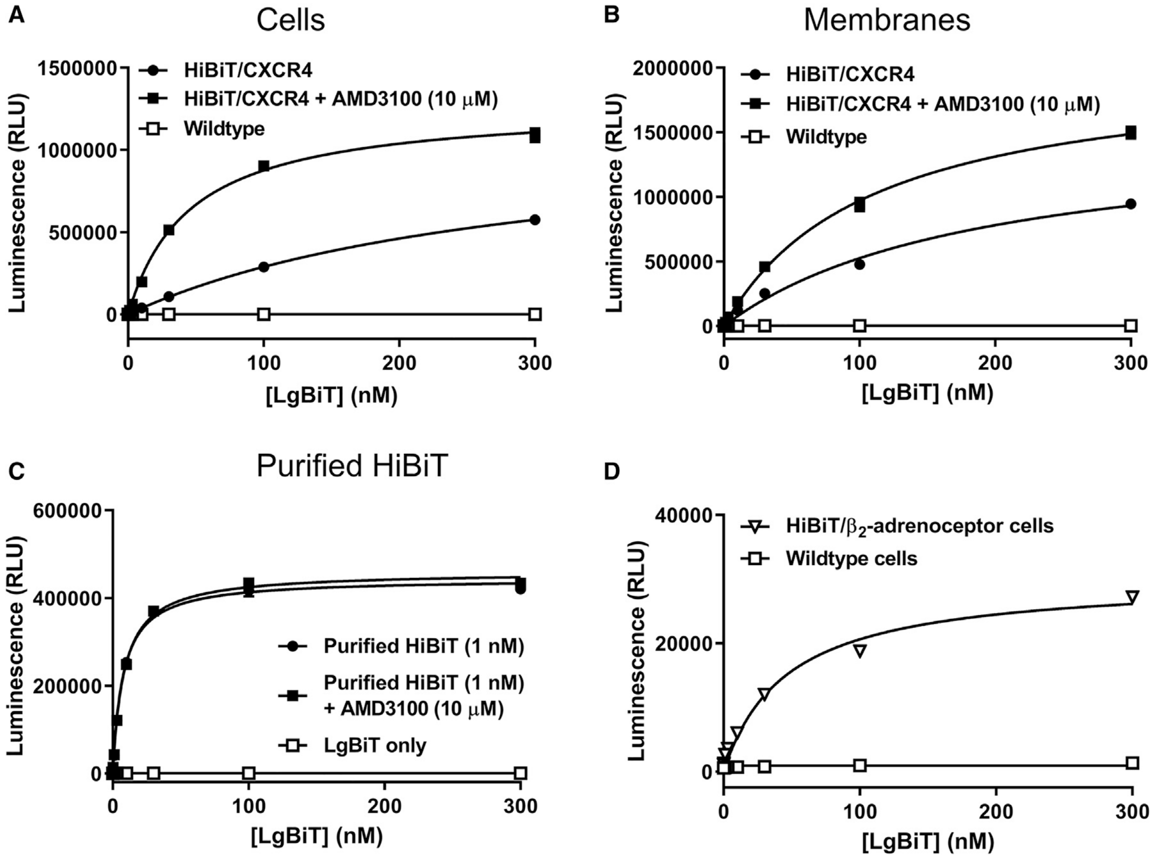

Figure 6. Investigation of the Effect of Protein Fusion on the Affinity of HiBiT-LgBiT Complementation

(A–C) HEK293 cells expressing genome-edited HiBiT/CXCR4 (black symbols) or wild-type HEK293 cells (white squares) (A), membranes from HEK293 cells expressing genome-edited HiBiT/CXCR4 (black symbols) and wild-type HEK293 cells (white squares) (B), or purified HiBiT control protein (black symbols) were incubated with increasing concentrations of LgBiT in the absence (black circles) or presence (black squares) of AMD3100 (10 μM) (C).

(D) HEK293 cells expressing genome-edited HiBiT/β_2-adrenoceptor (downward triangles) or wild-type HEK293 cells (white squares) were incubated with increasing concentrations of purified LgBiT. Points are mean ± SEM and are representative of five (C and D), six (B), or seven (A) experiments performed in triplicate.

et al., 2013), none of which are expressed endogenously at detectable levels in HEK293 or HeLa cells (Thul et al., 2017). The effect of ACKR3 interactions with CXCR4 appear to manifest in differences in signaling (Decaillot et al., 2011; Levoye et al., 2009). It is also possible that further intervention, e.g., activation of ACKR3, is required to observe any allosteric or cooperative differences in CXCL12 binding to CXCR4.

In contrast to ligand binding, the context in which a receptor is found can drastically affect signaling, with variations in the cellular proteome influencing function. However, many assays used to study receptor signaling disrupt the normal cellular balance by overexpression of the receptor or interacting effectors. A prototypical example of this is the use of overexpression fusion proteins to probe recruitment of β-arrestins to GPCRs, which can be used to investigate receptor desensitization, G protein-independent signaling, and establish biased agonism. However, β-arrestins are active participants in GPCR regulation with overexpression or deletion modulating the duration and magnitude of GPCR-mediated G protein signaling (Luttrell et al., 2018; Smith et al., 2019) as well as modulation of ligand binding (Gurevich et al., 1997). Using NanoBiT complementation, we have directly compared the effect of different combinations of genome-edited and exogenous proteins on the kinetic profile of ligand-induced β-arrestin2/SmBiT recruitment to CXCR4/LgBiT. The kinetic profiles from each configuration were obtained from a clonal line derived from a single heterozygously tagged cell, therefore, untagged CXCR4 and/or β-arrestin2 present, will result in non-productive interactions occurring. In addition, here we tagged both CXCR4 and β-arrestin2 on their respective C termini, which may restrict the ability of the two proteins to interact or alter specific geometry of the interaction compared with untagged proteins. Although both these factors may influence the profile of recruitment, the kinetics from the singularly genome-edited assay configurations are consistent with those observed previously in genome-edited NanoBRET assays (White et al., 2017). Notably, where CXCR4/LgBiT was expressed in excess to β-arrestin2/SmBiT the CXCL12-mediated increase in luminescence was slower than where β-arrestin2/SmBiT was in excess, suggesting a rate-limiting step when CXCR4 was overexpressed. Plausibly this is due to high exogenous CXCR4 expression overwhelming the capacity of endogenous GRKs to phosphorylate (Busillo

et al., 2010) the CXCR4 receptor population, therefore slowing down the subsequent kinetics of β-arrestin recruitment. Conversely, where β-arrestin2/SmBiT was in excess of CXCR4/LgBiT, we observed a rapid ligand-induced luminescence peak followed by a rapid decline and plateau, suggesting association then dissociation of CXCR4/LgBiT and β-arrestin2/SmBiT. β-Arrestins play a role in targeting CXCR4 for lysosomal degradation (Malik and Marchese, 2010), and in addition we have previously observed internalization and trafficking of genome-edited CXCR4 to lysosomes within a few minutes of agonist simulation (White et al., 2017). This suggests that the transient recruitment profile may be due to increased degradation of CXCR4 where β-arrestin2/SmBiT is overexpressed. Here, our data also highlight care must be taken if using the kinetic profile of β-arrestin recruitment to determine the stability of GPCR-β-arrestin interactions or designating subtle differences in the kinetic profiles of β-arrestin recruitment to GPCRs since they may be attributable to changes in protein expression. Finally, propagation of system/assay effects need to be controlled when examining biased GPCR agonism. Sources of error may include, variation in the receptor-effector levels due to the choice of cellular background and/or assay used, e.g., the use of overexpressed β-arrestin or G protein biosensors versus measurement of G protein signaling mediated by endogenous G proteins, as well as the kinetics of a signaling pathway (Klein Herenbrink et al., 2016; Galandrin et al., 2016). Coupling luciferase competition techniques with CRISPR/Cas9 genome editing as we have done here, which allows interactions between two natively expressed proteins to be observed, may therefore better recapitulate the "native" kinetics and stoichiometry of these protein-protein interactions when investigating ligand bias.

Following β-arrestin recruitment, GPCRs are internalized and investigating these processes are important for understanding receptor desensitization and recycling. Here, using cell-impermeant LgBiT and genome-edited HiBiT/CXCR4 or HiBiT/β_2-adrenoceptors we demonstrate that receptor internalization can be inferred from agonist-mediated decreases in luminescence, thereby confirming previous studies using the same assay format with overexpressed receptors. Unlike bystander BRET assays, however, which can monitor kinetics of receptor internalization, cell surface expression was determined by HiBiT/Receptor-LgBiT complementation at a single time point after agonist stimulation. Therefore, the decrease in luminescence is the sum of internalization, forward trafficking, and receptor recycling. Indeed, in our hands, kinetic analysis of the effect of CXCL12 on HiBiT/CXCR4 cell surface expression in live cell assay shows CXCL12 induced a small increase in luminescence before gradually declining, which would be in agreement with CXCL12 accumulation/compartmentalization of CXCR4 in the plasma membrane before internalization (Wysoczynski et al., 2005; White et al., 2017).

In addition, we demonstrate that receptors tagged with HiBiT on the N terminus do not necessarily report purely on ligand-induced changes in cell surface expression but in a novel assay configuration potentially also on extracellular conformational changes of exogenous or genome-edited receptors. Luminescence output is reliant on HiBiT-LgBiT complementation but, as seen in Figure 6, the affinity of HiBiT for purified LgBiT can be altered by fusion to a receptor or protein. These differences in affinity are likely due to steric hindrance imparted by the protein of interest. It follows that, and as we demonstrate for HiBiT/CXCR4, ligands that can modulate the conformation of a protein may, therefore, change the affinity of HiBiT-LgBiT complementation. Although these results were surprising and are an additional caveat to HiBiT-based internalization assays, differential NanoBiT affinity following fusion to a protein is not unexpected and can be exploited to investigate the effect of ligand binding on receptor conformation. Indeed, our results support the notion that AMD3100, in part, prevents binding of extracellular binders e.g., antibodies, HIV, as well as CXCL12 to CXCR4 by inducing conformational rearrangement of the extracellular domains despite itself binding in the transmembrane bundle (Rosenkilde et al., 2004). Furthermore, the agonist-mediated increase in luminescence at HiBiT-tagged ACKR3 likely suggests that there is a change in HiBiT-LgBiT complementation affinity at ACKR3 as well as different N-terminal orientation to CXCR4 and supports the observations of different CXCL12 binding modes between the two receptors (Benredjem et al., 2017; Gustavsson et al., 2017).

A final consideration for a change in affinity of tagged-HiBiT for LgBiT is its subsequent use to measure protein expression. Quantification by enzymatic activity assumes luminescence generated by the HiBiT-tagged protein is proportional to that generated by purified HiBiT (when complemented to a saturating concentration of LgBiT). However, as the affinity of tagged-HiBiT for LgBiT approaches or exceeds (as seen with HiBiT/CXCR4), the concentration of purified LgBiT that can be feasibly used, the assay underestimates the number of HiBiT-tagged proteins. Therefore, to ensure accurate protein quantification by this method, the affinity of tagged-HiBiT for LgBiT may need to be empirically determined for individual proteins and assay conditions or tag placement subsequently modified.

SIGNIFICANCE

In summary, we demonstrate the use of CRISPR/Cas9 genome editing to investigate multiple aspects of chemokine receptor function via NanoBRET- or NanoBiT-based assays where the proteins are expressed under endogenous promotion. We also show fluorescent ligand binding to genome-edited chemokine receptors and that nanoluciferase complementation can be used to monitor extracellular conformational changes following ligand binding in a live cell assay. In addition, we demonstrate that nanoluciferase complementation can be used to monitor ligand-induced receptor protein interactions where both partners are expressed under endogenous transcriptional control. These techniques have allowed us to examine the effect of protein expression on GPCR function and we show that the kinetic profile of β-arrestin2 recruitment to CXCR4 is dependent on the relative level of expression between the two proteins. These genome-editing techniques have the potential to generate cellular systems that more closely represent the "native" cellular environment, with minimal disruption to the normal cellular stoichiometry. Therefore these approaches may represent better models to investigate G protein-coupled receptor function and to understand how

changes in the cellular environment influences receptor signaling in human (patho)physiology.

STAR★METHODS

Detailed methods are provided in the online version of this paper and include the following:

- KEY RESOURCES TABLE
- LEAD CONTACT AND MATERIALS AVAILABILITY
- EXPERIMENTAL MODEL AND SUBJECT DETAILS
- METHOD DETAILS
  - Materials
  - Molecular Biology
  - CRISPR/Cas9 Genome Engineering
  - Cell Culture
  - Screening of Genome-Edited Clones
  - RTqPCR
  - Widefield Bioluminescence Microscopy
  - Membrane Preparation
  - Quantification of Tagged Protein by Luciferase Activity
  - NanoBRET Saturation Ligand Binding Assays
  - NanoBRET Competition Ligand Binding Assays in Non-clonal Cells
  - β-arrestin2 Recruitment Assays
  - NanoBiT Internalisation/Cell Surface Expression
  - Determination of NanoBiT Affinity
- QUANTIFICATION AND STATISTICAL ANALYSIS
  - Data Presentation and Statistical Analysis
- DATA AND CODE AVAILABILITY

SUPPLEMENTAL INFORMATION

Supplemental Information can be found online at https://doi.org/10.1016/j.chembiol.2020.01.010.

ACKNOWLEDGMENTS

This work was supported by MRC grant number MR/N020081/1 and a European Union Horizon 2020 MSCA Program grant (ONCORNET, agreement 641833). C.W.W. is supported by an NHMRC CJ Martin Fellowship (1088334) and by a UWA fellowship support grant.

AUTHOR CONTRIBUTIONS

C.W.W. and S.J.H. conceived the study. C.W.W. and B.C. generated reagents. C.W.W. and H.K.V. conducted the experiments. C.W.W. and H.K.V. performed the data analysis. C.W.W., H.K.V., B.C., K.D.G.P., and S.J.H. wrote or contributed to the writing of the manuscript.

DECLARATION OF INTERESTS

K.D.G.P. receives funding from Promega, BMG Labtech, and Dimerix as Australian Research Council Linkage Grant participating organizations. These participating organizations played no role in any aspect of the conception or design of the research, collection, analysis, and interpretation of results, or writing and editing of the manuscript. K.D.G.P. is Chief Scientific Advisor of Dimerix, of which he maintains a shareholding. The authors declare no other competing interests.

Received: October 7, 2019
Revised: January 2, 2020
Accepted: January 24, 2020
Published: February 12, 2020

REFERENCES

Benredjem, B., Girard, M., Rhainds, D., St-Onge, G., and Heveker, N. (2017). Mutational analysis of atypical chemokine receptor 3 (ACKR3/CXCR7) interaction with its chemokine ligands CXCL11 and CXCL12. J. Biol. Chem. *292*, 31–42.

Bockaert, J., Fagni, L., Dumuis, A., and Marin, P. (2004). GPCR interacting proteins (GIP). Pharmacol. Ther. *103*, 203–221.

Bouzo-Lorenzo, M., Stoddart, L.A., Xia, L., IJzerman, A.P., Heitman, L.H., Briddon, S.J., and Hill, S.J. (2019). A live cell NanoBRET binding assay allows the study of ligand-binding kinetics to the adenosine A3 receptor. Purinergic Signal. *15*, 139–153.

Busillo, J.M., Armando, S., Sengupta, R., Meucci, O., Bouvier, M., and Benovic, J.L. (2010). Site-specific phosphorylation of CXCR4 is dynamically regulated by multiple kinases and results in differential modulation of CXCR4 signaling. J. Biol. Chem. *285*, 7805–7817.

Carnec, X., Quan, L., Olson, W.C., Hazan, U., and Dragic, T. (2005). Anti-CXCR4 monoclonal antibodies recognizing overlapping epitopes differ significantly in their ability to inhibit entry of human immunodeficiency virus type 1. J. Virol. *79*, 1930–1933.

Caspar, B., Stoddart, L.A., Goulding, J., Briddon, S.J., and Hill, S.J. (2018). Effect of intracellular loop 1 pepducins on ligand-receptor binding kinetics at the CXCR4 receptor. Br. Pharmacol. Soc. http://www.pa2online.org/abstracts/Vol3Issue2abst001P.pdf.

Decaillot, F.M., Kazmi, M.A., Lin, Y., Ray-Saha, S., Sakmar, T.P., and Sachdev, P. (2011). CXCR7/CXCR4 heterodimer constitutively recruits beta-arrestin to enhance cell migration. J. Biol. Chem. *286*, 32188–32197.

Dixon, A.S., Schwinn, M.K., Hall, M.P., Zimmerman, K., Otto, P., Lubben, T.H., Butler, B.L., Binkowski, B.F., Machleidt, T., Kirkland, T.A., et al. (2016). NanoLuc complementation reporter optimized for accurate measurement of protein interactions in cells. ACS Chem. Biol. *11*, 400–408.

Ellisdon, A.M., and Halls, M.L. (2016). Compartmentalization of GPCR signalling controls unique cellular responses. Biochem. Soc. Trans. *44*, 562–567.

Galandrin, S., Onfroy, L., Poirot, M.C., Senard, J.M., and Gales, C. (2016). Delineating biased ligand efficacy at 7TM receptors from an experimental perspective. Int. J. Biochem. Cell Biol. *77*, 251–263.

Gurevich, V.V., Pals-Rylaarsdam, R., Benovic, J.L., Hosey, M.M., and Onorato, J.J. (1997). Agonist-receptor-arrestin, an alternative ternary complex with high agonist affinity. J. Biol. Chem. *272*, 28849–28852.

Gustavsson, M., Wang, L., Van Gils, N., Stephens, B.S., Zhang, P., Schall, T.J., Yang, S., Abagyan, R., Chance, M.R., Kufareva, I., and Handel, T.M. (2017). Structural basis of ligand interaction with atypical chemokine receptor 3. Nat. Commun. *8*, 14135.

Heuninck, J., Perpina Viciano, C., Isbilir, A., Caspar, B., Capoferri, D., Briddon, S.J., Durroux, T., Hill, S.J., Lohse, M.J., Milligan, G., et al. (2019). Context-dependent signalling of CXC chemokine receptor 4 (CXCR4) and atypical chemokine receptor 3 (ACKR3). Mol. Pharmacol. *96*, 778–793.

Kamiyama, D., Sekine, S., Barsi-Rhyne, B., Hu, J., Chen, B., Gilbert, L.A., Ishikawa, H., Leonetti, M.D., Marshall, W.F., et al. (2016). Versatile protein tagging in cells with split fluorescent protein. Nat. Commun. *7*, 11046.

Khan, A.O., White, C.W., Pike, J.A., Yule, J., Slater, A., Hill, S.J., Poulter, N.S., Thomas, S.G., and Morgan, N.V. (2019). Optimised insert design for improved single-molecule imaging and quantification through CRISPR-Cas9 mediated knock-in. Scientific Rep. *9*, 14219.

Kilpatrick, L.E., Alcobia, D.C., White, C.W., Peach, C.J., Glenn, J.R., Zimmerman, K., Kondrashov, A., Pfleger, K.D.G., Ohana, R.F., Robers, M.B., et al. (2019). Complex formation between VEGFR2 and the beta2-adrenoceptor. Cell Chem Biol *26*, 830–841.e9.

Kilpatrick, L.E., Friedman-Ohana, R., Alcobia, D.C., Riching, K., Peach, C.J., Wheal, A.J., Briddon, S.J., Robers, M.B., Zimmerman, K., Machleidt, T., et al. (2017). Real-time analysis of the binding of fluorescent VEGF165a to VEGFR2 in living cells: effect of receptor tyrosine kinase inhibitors and fate of internalized agonist-receptor complexes. Biochem. Pharmacol. *136*, 62–75.

Klein Herenbrink, C., Sykes, D.A., Donthamsetti, P., Canals, M., Coudrat, T., Shonberg, J., Scammells, P.J., Capuano, B., Sexton, P.M., Charlton, S.J., et al. (2016). The role of kinetic context in apparent biased agonism at GPCRs. Nat. Commun. *7*, 10842.

Lan, T.H., Liu, Q., Li, C., Wu, G., and Lambert, N.A. (2012). Sensitive and high resolution localization and tracking of membrane proteins in live cells with BRET. Traffic *13*, 1450–1456.

Laschet, C., Dupuis, N., and Hanson, J. (2019). A dynamic and screening-compatible nanoluciferase-based complementation assay enables profiling of individual GPCR-G protein interactions. J. Biol. Chem. *294*, 4079–4090.

Levoye, A., Balabanian, K., Baleux, F., Bachelerie, F., and Lagane, B. (2009). CXCR7 heterodimerizes with CXCR4 and regulates CXCL12-mediated G protein signaling. Blood *113*, 6085–6093.

Lohse, M.J., Nuber, S., and Hoffmann, C. (2012). Fluorescence/bioluminescence resonance energy transfer techniques to study G-protein-coupled receptor activation and signaling. Pharmacol. Rev. *64*, 299–336.

Luttrell, L.M., Wang, J., Plouffe, B., Smith, J.S., Yamani, L., Kaur, S., Jean-Charles, P.Y., Gauthier, C., Lee, M.H., Pani, B., et al. (2018). Manifold roles of beta-arrestins in GPCR signaling elucidated with siRNA and CRISPR/Cas9. Sci. Signal. *11*, eaat7650.

Magalhaes, A.C., Dunn, H., and Ferguson, S.S. (2012). Regulation of GPCR activity, trafficking and localization by GPCR-interacting proteins. Br. J. Pharmacol. *165*, 1717–1736.

Malik, R., and Marchese, A. (2010). Arrestin-2 interacts with the endosomal sorting complex required for transport machinery to modulate endosomal sorting of CXCR4. Mol. Biol. Cell *21*, 2529–2541.

May, L.T., Bridge, L.J., Stoddart, L.A., Briddon, S.J., and Hill, S.J. (2011). Allosteric interactions across native adenosine-A3 receptor homodimers: quantification using single-cell ligand-binding kinetics. FASEB J. *25*, 3465–3476.

Oh-Hashi, K., Furuta, E., Fujimura, K., and Hirata, Y. (2017). Application of a novel HiBiT peptide tag for monitoring ATF4 protein expression in Neuro2a cells. Biochem. Biophys. Rep. *12*, 40–45.

Oh-Hashi, K., Furuta, E., Norisada, J., Amaya, F., Hirata, Y., and Kiuchi, K. (2016). Application of NanoLuc to monitor the intrinsic promoter activity of GRP78 using the CRISPR/Cas9 system. Genes Cells *21*, 1137–1143.

Pelekanos, R.A., Ting, M.J., Sardesai, V.S., Ryan, J.M., Lim, Y.C., Chan, J.K., and Fisk, N.M. (2014). Intracellular trafficking and endocytosis of CXCR4 in fetal mesenchymal stem/stromal cells. BMC Cell Biol *15*, 15.

Rajagopal, S., Kim, J., Ahn, S., Craig, S., Lam, C.M., Gerard, N.P., Gerard, C., and Lefkowitz, R.J. (2010). Beta-arrestin- but not G protein-mediated signaling by the "decoy" receptor CXCR7. Proc. Natl. Acad. Sci. U S A *107*, 628–632.

Ran, F.A., Hsu, P.D., Wright, J., Agarwala, V., Scott, D.A., and Zhang, F. (2013). Genome engineering using the CRISPR-Cas9 system. Nat. Protoc. *8*, 2281–2308.

Rankovic, Z., Brust, T.F., and Bohn, L.M. (2016). Biased agonism: an emerging paradigm in GPCR drug discovery. Bioorg. Med. Chem. Lett. *26*, 241–250.

Reyes-Alcaraz, A., Lee, Y.N., Yun, S., Hwang, J.I., and Seong, J.Y. (2018). Conformational signatures in beta-arrestin2 reveal natural biased agonism at a G-protein-coupled receptor. Commun. Biol. *1*, 128.

Rose, R.H., Briddon, S.J., and Hill, S.J. (2012). A novel fluorescent histamine H(1) receptor antagonist demonstrates the advantage of using fluorescence correlation spectroscopy to study the binding of lipophilic ligands. Br. J. Pharmacol. *165*, 1789–1800.

Rosenkilde, M.M., Gerlach, L.O., Jakobsen, J.S., Skerlj, R.T., Bridger, G.J., and Schwartz, T.W. (2004). Molecular mechanism of AMD3100 antagonism in the CXCR4 receptor: transfer of binding site to the CXCR3 receptor. J. Biol. Chem. *279*, 3033–3041.

Rouault, A.A.J., Lee, A.A., and Sebag, J.A. (2017). Regions of MRAP2 required for the inhibition of orexin and prokineticin receptor signaling. Biochim. Biophys. Acta *1864*, 2322–2329.

Schwinn, M.K., Machleidt, T., Zimmerman, K., Eggers, C.T., Dixon, A.S., Hurst, R., Hall, M.P., Encell, L.P., Binkowski, B.F., and Wood, K.V. (2018). CRISPR-mediated tagging of endogenous proteins with a luminescent peptide. ACS Chem. Biol. *13*, 467–474.

Smith, J.S., Pack, T.F., Inoue, A., Lee, C., Xiong, X., Zheng, K., Kahsai, A.W., Choi, I., Ma, Z., Levitan, I.M., et al. (2019). Noncanonical scaffolding of $G_{\alpha i}$ and β-arrestin by G protein-coupled receptors. bioRxiv. . 629576. https://doi.org/10.1101/629576.

Sohy, D., Yano, H., De Nadai, P., Urizar, E., Guillabert, A., Javitch, J.A., Parmentier, M., and Springael, J.Y. (2009). Hetero-oligomerization of CCR2, CCR5, and CXCR4 and the protean effects of "selective" antagonists. J. Biol. Chem. *284*, 31270–31279.

Stepanenko, A.A., and Dmitrenko, V.V. (2015). HEK293 in cell biology and cancer research: phenotype, karyotype, tumorigenicity, and stress-induced genome-phenotype evolution. Gene *569*, 182–190.

Stoddart, L.A., Johnstone, E.K.M., Wheal, A.J., Goulding, J., Robers, M.B., Machleidt, T., Wood, K.V., Hill, S.J., and Pfleger, K.D.G. (2015). Application of BRET to monitor ligand binding to GPCRs. Nat. Methods *12*, 661–663.

Storme, J., Cannaert, A., Van Craenenbroeck, K., and Stove, C.P. (2018). Molecular dissection of the human A3 adenosine receptor coupling with beta-arrestin2. Biochem. Pharmacol. *148*, 298–307.

Thul, P.J., Akesson, L., Wiking, M., Mahdessian, D., Geladaki, A., Ait Blal, H., Alm, T., Asplund, A., Bjork, L., Breckels, L.M., et al. (2017). A subcellular map of the human proteome. Science *356*, eaal3321.

Tiulpakov, A., White, C.W., Abhayawardana, R.S., See, H.B., Chan, A.S., Seeber, R.M., Heng, J.I., Dedov, I., Pavlos, N.J., and Pfleger, K.D. (2016). Mutations of vasopressin receptor 2 including novel L312S have differential effects on trafficking. Mol. Endocrinol. *30*, 889–904.

Tsvetanova, N.G., Irannejad, R., and Von Zastrow, M. (2015). G protein-coupled receptor (GPCR) signaling via heterotrimeric G proteins from endosomes. J. Biol. Chem. *290*, 6689–6696.

Walther, C., and Ferguson, S.S. (2015). Minireview: role of intracellular scaffolding proteins in the regulation of endocrine G protein-coupled receptor signaling. Mol. Endocrinol. *29*, 814–830.

Wang, W., Qiao, Y., and Li, Z. (2018). New insights into modes of GPCR activation. Trends Pharmacol. Sci. *39*, 367–386.

Watts, A.O., Van Lipzig, M.M., Jaeger, W.C., Seeber, R.M., Van Zwam, M., Vinet, J., Van Der Lee, M.M., Siderius, M., Zaman, G.J., et al. (2013). Identification and profiling of CXCR3-CXCR4 chemokine receptor heteromer complexes. Br. J. Pharmacol. *168*, 1662–1674.

White, C.W., and Hill, S.J. (2019). Using CRISPR/Cas9 and NanoLuc to investigate "endogenous" CXCR4 ligand binding, internalization and β-arrestin2 recruitment. FASEB J. *33*, 811.4.

White, C.W., Johnstone, E.K.M., See, H.B., and Pfleger, K.D.G. (2019). NanoBRET ligand binding at a GPCR under endogenous promotion facilitated by CRISPR/Cas9 genome editing. Cell Signal *54*, 27–34.

White, C.W., Vanyai, H.K., See, H.B., Johnstone, E.K.M., and Pfleger, K.D.G. (2017). Using nanoBRET and CRISPR/Cas9 to monitor proximity to a genome-edited protein in real-time. Sci. Rep. *7*, 3187.

Wysoczynski, M., Reca, R., Ratajczak, J., Kucia, M., Shirvaikar, N., Honczarenko, M., Mills, M., Wanzeck, J., Janowska-Wieczorek, A., and Ratajczak, M.Z. (2005). Incorporation of CXCR4 into membrane lipid rafts primes homing-related responses of hematopoietic stem/progenitor cells to an SDF-1 gradient. Blood *105*, 40–48.

Yang, Y., Guo, Y., Tan, S., Ke, B., Tao, J., Liu, H., Jiang, J., Chen, J., Chen, G., and Wu, B. (2015). β-Arrestin1 enhances hepatocellular carcinogenesis through inflammation-mediated Akt signalling. Nat. Commun. *6*, 7369.

Zhang, X.H., Tee, L.Y., Wang, X.G., Huang, Q.S., and Yang, S.H. (2015). Off-target effects in CRISPR/Cas9-mediated genome engineering. Mol. Ther. Nucleic Acids *4*, e264.

STAR★METHODS

KEY RESOURCES TABLE

REAGENT or RESOURCE	SOURCE	IDENTIFIER
Chemicals, Peptides, and Recombinant Proteins		
AMD3100	Selleckchem	Cat # S8030
Covine serum albumin (Protease-free)	Sigma Aldrich	Cat# 03117332001
CXCL11	Preprotech	Cat# 300-46
CXCL12	Preprotech	Cat# 300-28A
CXCL12-AF647	Almac	Cat# CAF-11
N,N'-Dicyclohexylcarbamimidothioic acid (5,6-dihydro-6,6-dimethylimidazo[2,1-b]thiazol-3-yl)methyl ester dihydrochloride (IT1t)	Tocris	Cat# 4596
Dulbecco's Modified Eagle's Medium	Sigma Aldrich	Cat# D6429
Fetal Bovine Serum	Sigma Aldrich	Cat# F2442
Fugene HD	Promega (Wisconsin, USA)	Cat# E2311
Geneticin™ (G418)	ThermoFisher	Cat# 10131035
HiBiT-Halotag, control peptide	Promega (Wisconsin, USA)	Cat# N3010
Isoprenaline hydrochloride	Sigma Aldrich	Cat# I6504
Phosphate Buffered Saline (PBS)	Sigma Aldrich	Cat# D8537
Poly-D-Lysine hydrobromide	Sigma Aldrich	Cat# P6407
(±)-propranolol hydrochloride	Sigma Aldrich	Cat# P0884
Purified LgBiT	Promega (Wisconsin, USA)	Cat# N401A
Purified full length NLuc	Promega (Wisconsin, USA)	Gift from Matt Robers (Promega)
Puromycin dihydrochloride from Streptomyces alboniger	Sigma Aldrich	Cat# P8833
Saponin	Sigma Aldrich	Cat# 84510
Xanthine amine congener (XAC)	Sigma Aldrich	Cat# X103
Critical Commercial Assays		
Maxima First Strand cDNA Synthesis Kit	Thermo Fisher Scientific	Cat# K1641
Nano-Glo luciferase assay system (Furimazine)	Promega (Wisconsin, USA)	Cat# N1130
Pierce™ bicinchoninic acid protein assay kit	Thermo Fisher Scientific	Cat# 23225
PowerUp SYBR Green Master Mix	Thermo Fisher Scientific	Cat# A25742
ReliaPrep™ RNA extraction kit	Promega Corporation (Wisconsin,USA)	Cat# Z6010
Hs_B2M_1_SQ QuantiTect Primer Assay	Qiagen	Cat# QT00088935
Experimental Models: Cell Lines		
Human HEK293FT cells (female)	Life Technologies	Cat# R70007
Human HeLa cells (female)	Laboratory of Stephen Briddon (University of Nottingham)	Rose et al., 2012
Oligonucleotides		
Oligonucleotides for sgRNA construction, see Table S1	Sigma Aldrich	Custom Synthesis
Oligonucleotides for PCR amplification, see Table S1	Sigma Aldrich	Custom Synthesis
Oligonucleotides for site directed mutagenesis, see Method Details - *Molecular Biology*	Sigma Aldrich	Custom Synthesis
Single stranded oligonucleotide for *ADRB2* homology directed repair template, see Table S2	Integrated DNA Technologies	Custom Synthesis
Recombinant DNA		
β-arrestin2-SmBiT	This manuscript	Custom synthesis
β-arrestin2-Halotag	Tiulpakov et al., 2016	Custom synthesis

(*Continued on next page*)

Continued

REAGENT or RESOURCE	SOURCE	IDENTIFIER
cDNA encoding NSGSSGGGGSGGGGSSG-LgBiT for sub-cloning	GeneArt (Thermo Fisher Scientific)	Custom synthesis
CXCR4-LgBiT	This manuscript	Custom synthesis
HiBiT-ACKR3	This manuscript	Custom synthesis
HiBiT-CXCR4	This manuscript	Custom synthesis
Homology directed repair templates, see Table S2	GeneArt (Thermo Fisher Scientific)	Custom synthesis
NanoLuc-ACRK3	This manuscript	Custom synthesis
NanoLuc-CXCR4	This manuscript	Custom synthesis
pSpCas9(BB)-2A-Puro (PX459) V2.0	Addgene Plasmid	Cat # 62988; RRID: Addgene_62988
Software and Algorithms		
GraphPad Prism 7.02	GraphPad Software, La Jolla California USA	https://www.graphpad.com/scientific-software/prism/
Other		
35 mm dish containing a high tolerance 1.5 μm coverslip	MatTek	Cat# P35G-0.170-14-C
BamHI-HF Restriction enzyme	New England Biolabs (UK)	Cat# R3136
Kpn-HF Restriction enzyme	New England Biolabs (UK)	Cat# R3142
PHERAStar FS plate reader	BMGLabTech	PHERAStar FS plate reader
Olympus LV200 wide field inverted microscope	Olympus	Olympus LV200 wide field inverted microscope
Q5® High-Fidelity DNA Polymerase	New England Biolabs (UK)	Cat# M0491
XbaI Restriction enzyme	New England Biolabs (UK)	Cat# R0145
XhoI Restriction enzyme	New England Biolabs (UK)	Cat# R0146
White 96-well plates	Greiner Bio-One	Cat# 655089

LEAD CONTACT AND MATERIALS AVAILABILITY

Further information and requests for resources and reagents should be directed to and will be fulfilled by the Lead Contact, Stephen J Hill (stephen.hill@nottingham.ac.uk). All unique/stable reagents generated in this study are available from the Lead Contact without restriction.

EXPERIMENTAL MODEL AND SUBJECT DETAILS

Human HEK293FT cells (female) were obtained from (Life Technologies). Human HeLa cells (female) were obtained from Dr. Stephen Briddon (University of Nottingham) (Rose et al., 2012). Cells lines were not subsequently authenticated. HeLa and HEK293T cells were transfected and cultured as described in Method Details.

METHOD DETAILS

Materials

AMD3100 was purchased from Selleckchem (USA), CXCL11 and CXCL12 were purchased from Preprotech (USA). CXCL12-AF647 was purchased from Almac (United Kingdom). N,N'-Dicyclohexylcarbamimidothioic acid (5,6-dihydro-6,6-dimethylimidazo[2,1-b] thiazol-3-yl)methyl ester dihydrochloride (IT1t) was purchased from Tocris (United Kingdom)., isoprenaline hydrochloride, (±)-propranolol hydrochloride, saponin, and xanthine amine congener (XAC) were from Sigma-Aldrich, (United Kingdom). Furimazine, purified HiBiT (HiBiT-Halotag, control peptide) and purified LgBiT NLuc fragments were purchased from Promega (USA), affinities and modifications of the fragments from the native NLuc protein have been described previously by the manufacturer (Dixon et al., 2016). Purified full length NLuc was a kind gift from Matt Robers (Promega, USA). AM3100, isporenaline, and propranolol were dissolved in water, CXCL11, CXCL12 and CXCL12-AF647 were dissolved as per the manufacturer's instructions. Isoprenaline, IT1t (10 mM), propranolol (10 mM) and XAC (10 mM) were dissolved in DMSO. All further dilutions were performed in assay buffer containing 0.1% bovine serum albumin (BSA, Sigma-Aldrich, United Kingdom).

Molecular Biology

The CXCR4 and ACKR3 cDNA sequences were provided through the ONCORNET consortium from Vrije Universiteit Amsterdam in pcDEF3 plasmids. To generate pCDNA3.1 (+) neo expression constructs encoding NLuc/CXCR4 an internal BamHI restriction site was first removed by site directed mutagenesis. The primers used were forward 5’-GGCGTCTGGATTCCTGCCCTCCTGC-3’ and reverse 5’- GCAGGAGGGCAGGAATCCAGACGCC -3’. The mutated CXCR4 sequence was then PCR amplified to generate a CXCR4 sequence that was in frame with the BamHI restriction site of sig-NLuc (Stoddart et al., 2015), and changed the start codon (Met) of the CXCR4 sequence to (Leu). The primers used were forward 5’-CCCGGATCCCTGGAGGGGATCAGTATATAC-3’ and reverse 5’-GGGCTCGAGTTAGCTGGAGTGAAAACTTG-3’. To generate pCDNA3.1 (+) neo expression constructs encoding NLuc/ ACKR3, ACKR3 was PCR amplified to generate an ACKR3 sequence that was in frame with the BamHI restriction site of sig-NLuc, and changed the start codon (Met) of the ACKR3 sequence to (Leu). The primers used were forward 5’-CCCGGATCCCTG GATCTGCATCTCTTCG-3’ and reverse 5’-GGGCTCGAGTCATTTGGTGCTCTGCTCC-3’. Additional deoxyadenosines were added to both ACKR3 and CXCR4 PCR products by incubation Taq polymerase and then ligated into a pcDNA2.1 vector by standard TA cloning. The ACKR3 and mutated CXCR4 sequences were then ligated inframe from the pcDNA2.1 vector into pcDNA3.1 (+) neo vectors containing sig-NLuc using the restriction enzymes BamHI and XhoI. pCDNA3.1 (+) neo constructs encoding HiBiT/ CXCR4 or HiBiT/ACKR3 were generated by ligation of HiBiT-GSSG into NLuc/CXCR4 or NLuc/ACKR3 plasmid constructs digested with KpnI and BamHI using the complementary oligonucleotides 5’-CATGGTGAGCGGCTGGCGGCTGTTCAAGAAGATTAGC GGGAGTTCTGGCGGCTCGAGCGGTG-3’ and 5’-GATCCACCGCTCGAGCCGCCAGAACTCCCGCTAATCTTCTTGAACAGCCGC CAGCCGCTCACCATGGTAC -3’. To generate the CXCR4/LgBiT pcDNA3.1 expression construct, NSGSSGGGGSGGGGSSG-LgBiT synthesised by GeneArt was sub-cloned into pcDNA3.1 CXCR4 (White et al., 2017) using the restriction enzymes XhoI and XbaI. The cDNA expression construct encoding β-arrestin2/Halotag has been described previously (Tiulpakov et al., 2016). To generate the cDNA expression construct encoding β-arrestin2/NSGSSGGGGSGGGGSSG-SmBiT, cDNA sequences encoding linker-SmBiT where provided by Promega in a pNBe2 vector. An internal XhoI restriction site was first removed by site directed mutagenesis. The primers used were forward 5’- CGCCACCACCGCTGGAGCCAGAATTCC -3’ and reverse 5’- GGAATTCTGGCTCC AGCGGTGGTGGCG -3’. An in frame XhoI restriction site was then inserted by site directed mutagenesis. The primers used were forward 5’- CTCGAGCCAGAATTCTCGAGAGCTCCCACGGCGA -3’ and reverse 5’- TCGCCGTGGGAGCTCTCGAGAATTCTG GCTCGAG -3’. The resulting NSGSSGGGGSGGGGSSG-SmBiT fragment was then sub-cloned in frame into a pcDNA3.1 expression construct encoding β-arrestin2 described previously (Tiulpakov et al., 2016) using the restrictions enzymes XhoI and XbaI.

CRISPR/Cas9 Genome Engineering

Guide RNA construction was performed as described previously in the detailed protocol (Ran et al., 2013). Briefly, guide sequences were designed using the CRISPR Design Tool (http://crispr.mit.edu/) and ligated as complementary oligonucleotides (Table S1) into the pSpCas9(BB)-2A-Puro (PX459 V2) expression construct (from Feng Zhang, Addgene plasmid # 62988) linearized by the restriction enzyme BbsI (NEB). Guide sequences used to target Cas9 to the genomic loci were; for *CXCR4* N-terminus; guideRNA1, ATCCCCTCCATGGTAACCGC, and guideRNA2, TGGAGAACCAGCGGTTACCA, for *ACKR3* N-terminus; guideRNA1, GATTGCCCGCCTCAGAACGA and guideRNA2, GATGCAGATCCATCGTTCTG, to knockout *CXCL12* by InDel formation Cas9 was targeted to the N-terminal region using guideRNA1, GGCATGGGCATCTGTAGCTC and guideRNA2, CATCTGTAGCTCAGGCT GAC. The guide RNA sequences used to target the *CXCR4* and *ARRB2* C-terminus have been described previously (White et al., 2017), as have the guide RNA sequences used to target the N-terminus of *ADRB2* (Kilpatrick et al., 2019).

To introduce DNA encoding NLuc or NanoBiT fragments donor repair templates (Table S2) were designed using the UCSC genome browser (http://genome.ucsc.edu/), Human genome assembly (GRCh38/hg38). Briefly, for N-terminal tagging of CXCR4 with NLuc (NLuc/CXCR4) a donor template consisting of left homology arm - sig-NLuc – right homology arm surrounding but not including the genomic start codon were synthesized as double stranded DNA in pMX cloning vectors by GeneArt (Invitrogen). To introduce HiBiT to the N-terminus of CXCR4 (HiBiT/CXCR4), an internal KpnI restriction site in the repair template was silently mutated, using the primers 5’-CCAGGACATTGGAGGTGCCCGTACTCCAAAAAAG-3’ and 5’-CTTTTTTGGAGTACGGGCACCTCCAATGTCCTGG-3’, a KpnI restriction site was then introduced at the end of the left homology arm using the primers 5’-GAGAACCAGCGGGTACCAT GAGGTTG-3’ and 5’-CAACCTCATGGTACCCGCTGGTTCTC-3’ to allow ligation of HiBiT-GSSG into the template using the restriction enzymes KpnI and BamHI and the complementary oligonucleotides 5’-CATGGTGAGCGGCTGGCGGCTGTTCAAGAAGATT AGCGGGAGTTCTGGCGGCTCGAGCGGTG-3’ and 5’-GATCCACCGCTCGAGCCGCCAGAACTCCCGCTAATCTTCTTGAACAGCC GCCAGCCGCTCACCATGGTAC -3’. For N-terminal tagging of ACRK3 (NLuc/ACKR3) a donor template consisting of areas of homology surrounding but not including the ACKR3 start codon were synthesized as double stranded DNA by GeneArt (Invitrogen) a short linker was included between the homology arms to allow ligation of sig-NLuc into the template using the restriction enzymes KpnI and BamHI. The donor templates for N-terminus tagging therefore resulted in cells expressing genome-edited sig-Nluc or HiBiT-GSSG receptor with the start codon (Met) of the receptor deleted. For tagging the N-terminus of *ADRB2* with HiBiT, a repair template was synthesised as a single stranded oligo DNA nucleotide (ssODN; Integrated DNA Technologies; IDT) and consisted of homology arms surrounding HiBiT-GSSG with the start codon (Met) of *ADRB2* deleted. To insert SmBiT into the *ARRB2* genomic loci (β-arrestin2/ SmBiT) a donor template consisting of homology arms surrounding but not including the *ARRB2* stop codon was synthesized as double stranded DNA by GeneArt (Invitrogen). A short linker was included between the homology arms to allow ligation of GGGGSGGGGGSSG-SmBiT into the template using the restriction enzymes XhoI and XbaI and the complementary oligonucleotides 5’- TCGAGGGTGGTGGCGGGAGCGGAGGTGGAGGGTCGTCAGGTGTGACCGGCTACCGGCTGTTCGAGGAGATTCTGTAAT-3’

and 5'- CTAGATTACAGAATCTCCTCGAACAGCCGGTAGCCGGTCACACCTGACGACCCTCCACCTCCGCTCCCGCCACCACCC-3. To tag CXCR4 on the C-terminus with LgBiT (CXCR4/LgBiT), NSGSSGGGGSGGGGSSG-LgBiT was sub-cloned from the pcDNA3.1 CXCR4/LgBiT construct into the CXCR4 C-terminal repair template (White et al., 2017) using the restriction enzymes XhoI and XbaI.

Cell Culture

HEK293T or HeLa cells were maintained in Dulbecco's Modified Eagle's Medium (Sigma Aldrich) supplemented with 10 % fetal calf serum at 37°C/5% CO_2. Transfections were performed using FuGENE (Promega, USA) according to the manufacturer's instructions. Cell were passaged or harvested when cells reached 70-80% confluency using Phosphate Buffered Saline (PBS, Sigma Aldrich) and trypsin (0.25% w/v in versene; Sigma Aldrich). To generate cells stably expressing tagged receptors, cells were transfected with a pcDNA3.1 (+) neo expression vector encoding NLuc/CXCR4, HiBiT/CXCR4, NLuc/ACKR3 or HiBiT/ACKR3 and subsequently selected for incorporation of the transgene using G418 (ThermoFisher). CRISPR/Cas9 genome-engineering of HEK293 cells was performed as described previously (White et al., 2017; Ran et al., 2013). Briefly, HEK293 or HeLa cells were seeded in 6 well plates at 300,000 cells per well and incubated for 24h at 37°C/5% CO_2. Cells were then transfected with px459 sgRNA/Cas9 expression constructs and either plasmid or ssODN encoding for the donor repair template. Cells were cultured for 24h then treated with puromycin (0.3 μg/ml, Sigma-Aldrich) for 3 days to select for transfected cells. Following selection, cells were cultured without puromyocin for 1 day then seeded into clear flat bottom 96-well plates at 1 cell per well and allowed to expand for 2-3 weeks. To knockout CXCL12, HEK293 cells were transfected with px459 sgRNA/Cas9 expression constructs targeting the first exon of CXCL12 and selected and cloned as per the method used for tagging. To create cells expressing both β-arrestin2/SmBiT and CXCR4/LgBiT, cells expressing β-arrestin2/SmBiT were first generated then CXCR4 was tagged with LgBiT in a subsequent round of transfection and clonal isolation.

Screening of Genome-Edited Clones

Following clonal expansion single colonies expressing NLuc/CXCR4, NLuc/ACKR3 or CXCR4/LgBiT and β-arrestin2/SmBiT were screened for luminescence following the addition of furimazine (10 μM) using a PHERAStar FS plate reader. Clones expressing HiBiT/CXCR4 or HiBiT/β_2-adrenoceptor were screened by addition of furimazine (10 μM) and purified LgBiT (10 nM). Cells expressing CXCR4/LgBiT were lysed and screened for luminescence following the addition of furimazine (10 μM) and purified HiBiT (10 nM, Promega). To screen for clones expressing β-arrestin2/SmBiT following expansion, cells were harvested and seeded into poly-D-lysine coated white flat bottom 96 well plates and transiently transfected with a pcDNA3.1 expression vector encoding CXCR4/LgBiT (0.025 μg/well) using FuGENE and incubated for 24h. On the day of screening, cells were washed and incubated with pre-warmed 1x HEPES Buffered Salt Solution (1xHBSS; 25mM HEPES, 10mM glucose, 146mM NaCl, 5mM KCl, 1mM $MgSO_4$, 2mM sodium pyruvate, 1.3mM $CaCl_2$, 1.8g/L glucose; pH 7.2), for 1h. Cells were then incubated with furimazine (10 μM) for 5 minutes at 37°C before total light emissions were measured on a PHERAStar FS plate reader before and after the addition of CXCL12 (100 nM). Positive clones displayed an increase in luminescence following ligand addition. Positive clones were collected for genotyping and/or mRNA quantification by RTqPCR. Genotyping was performed by PCR amplification of genomic DNA using Q5® High-Fidelity DNA Polymerase (New England BioLabs) as per the manufacturer's instructions and primer sets described in Table S1. Heterozygous insertion of tags into the genomic loci was observed for all cell lines tested.

RTqPCR

Total RNA from wildtype or genome-edited cells was extracted using a ReliaPrep™ RNA extraction kit (Promega) as per the manufacturer's instructions followed by cDNA synthesis using the Maxima First Strand cDNA Synthesis Kit (Thermo Fisher Scientific) following manufacturer's instructions. qPCR was performed on QuantStudio 7 (Applied Biosystems) using PowerUp SYBR Green Master Mix (Thermo Fisher Scientific) and primers listed in Table S1. RTqPCR primers designed in-house except for Human *ARRB2* primers which were described previously (Yang et al., 2015). Target gene expression was normalised to B2M expression, amplified using Hs_B2M_1_SQ QuantiTect Primer Assay (QT00088935; Qiagen).

Widefield Bioluminescence Microscopy

Bioluminescence imaging was performed using an Olympus LV200 wide field inverted microscope, equipped with a 60x/1.42NA oil immersion objective lens and 0.5x tube lens. 24h before imaging cells were seeded into a 35 mm dish containing a high tolerance 1.5 μm coverslip (MatTek). On the day of imaging, medium was removed and cells were incubated with 2 mL HBSS for 30 minutes at 37° C before furimazine (400 nM) was added and allowed to equilibrate for 5 minutes at 37°C. Luminescence images were taken by capturing total luminescence for (90 sec exposure time). HiBiT/CXCR4 cells were incubated with furimazine (400 nM) and purified LgBiT (10 nM) for 5 minutes at 37°C prior to imaging.

Membrane Preparation

Membrane preparations were made as described previously (Bouzo-Lorenzo et al., 2019). Briefly, cells expressing NLuc or HiBiT tagged receptors were grown to 80-90% confluence in 500 cm^2 dishes or T175 flasks. Cells were washed with PBS and collected using a cell scraper or by pre-warmed non-enzymatic dissociation solution (PBS containing 0.2 g/L EDTA), cells were then pelleted, resuspended in ice-cold PBS and homogenised. Unbroken cells and nuclear fraction were removed by centrifugation at 1200 x g for 10 minutes at 4°C before the supernatant was centrifuged at 40,000 x g for 30 minute at 4°C to obtain the membrane fraction. The

membrane pellet was then resuspended and homogenised in ice cold PBS, before protein concentration was determined using a bicinchoninic acid protein assay kit (Thermofisher).

Quantification of Tagged Protein by Luciferase Activity

Quantification of NLuc-tagged receptors expressed in genome-edited cells was determined by interpolation against a purified NLuc standard curve, (R^2 = 0.994 ± 0.0017, Slope = 0.94 ± 0.016, slopes not different from unity p>0.05, mean ± s.e.m). On the day of assay, membrane preparations made from genome-edited HEK293 or HeLa cells were diluted to 1 μg/well in HBSS supplemented with 0.1% BSA and loaded into a white flat bottom 96 well plate in triplicate. A log NLuc standard curve (10 fmol - 100 nM) was constructed in parallel by diluting purified NLuc in HBSS supplemented with 0.1% BSA and adding to wells containing 1 μg/well wildtype HEK293 membranes. Plates were incubated for 10 minutes at 37°C before 10 μM furimazine was added. Total light emissions were measured on a PHERAStar FS plate reader after a further 5 minutes incubation.

NanoBRET Saturation Ligand Binding Assays

Genome-edited or cells stably expressing NLuc/CXCR4 or NLuc/ACKR3 were seeded into poly-D-lysine coated white flat bottom 96 well plates at 30,000 cells/well and incubated for 24h at 37°C/5% CO_2. On the day of the assay, cells were washed and incubated with pre-warmed HBSS supplemented with 0.1% BSA. For assays using membrane preparations, 10 μg membrane protein diluted in HBSS 0.1% BSA was loaded into each well on the day of assay. Cells or membranes were then incubated with increasing concentrations of CXCL12-AF647 in the absence or presence of AMD3100 (10 μM) or CXCL11 (10 μM) for NLuc or HiBiT/CXCR4 and NLuc/ACKR3 respectively for 60 minutes at 37°C. Following ligand incubation, 10 μM of the NLuc substrate furimazine was added and plates equilibrated for 5 minutes at room temperature. For cells expressing HiBiT/CXCR4 following ligand incubation both furimazine (10 μM) and purified LgBiT (10 nM) were added. Sequential filtered light emissions were taken using a PHERAStar FS plate reader using 460nm (80nm bandpass) and >610nm (longpass) filters. BRET ratios were calculated by dividing the 610nm emission (acceptor) by the 460nm emission (donor).

NanoBRET Competition Ligand Binding Assays in Non-clonal Cells

HEK293 cells were seeded in 6 well plates at 300,000 cells per well and incubated for 24h at 37°C/5% CO_2, cells were then transfected with px459 sgRNA/Cas9 expression constructs and plasmid encoding the NLuc/CXCR4 donor repair template or for the negative control, plasmid encoding the NLuc/CXCR4 donor repair template only and untargeted px459 sgRNA/Cas9 expression constructs. Cells were cultured for 24h then treated with puromycin (0.3 μg/mL) for 2 days to select for transfected cells. Cells were then allowed to recover and expand for three days. Cells were then seeded into poly-D-lysine coated white flat bottom 96 well plates at 30,000 cells/well and incubated for 24h at 37°C/5% CO_2. On the day of the assay, cells were washed and incubated with pre-warmed HBSS supplemented with 0.1% BSA. Cells were incubated with CXCL12-AF647 (12.5 nM) in the absence or presence of AMD3100 (10 pM – 10 μM) for 60 minutes at 37°C. Following ligand incubation, 10 μM of the NLuc substrate furimazine was added and plates equilibrated for 5 minutes at room temperature. Sequential filtered light emissions were taken using a PHERAStar FS plate reader using 460nm (80nm bandpass) and >610nm (longpass) filters. BRET ratios were calculated by dividing the 610nm emission (acceptor) by the 460nm emission (donor).

β-arrestin2 Recruitment Assays

Wildtype HEK293 cells or HEK293 cells expressing genome-edited β-arrestin2/SmBiT or genome-edited CXCR4/LgBiT were seeded in 6 well plates at 300,000 cells per well and incubated for 24h at 37°C/5% CO_2. Wildtype HEK293 cells were then transfected with plasmid DNA encoding CXCR4/LgBiT and β-arrestin2/SmBiT (25 ng of each per well of a 6 well plate). For HEK293 cells expressing genome-edited β-arrestin2/SmBiT or CXCR4/LgBiT, cells were transfected with CXCR4/LgBiT only or β-arrestin2/SmBiT only respectively (25 ng of plasmid DNA per well of a 6 well plate). 25ng plasmid DNA was chosen to approximate the levels of CXCR4 and β-arrestin2 expression we observed previously in genome-edited HEK293 cells (White et al., 2017). Cells were then incubated for 24h at 37°C/5% CO_2 before being seeded into poly-D-lysine coated white flat bottom 96 well plates, at 30,000 cells/well and incubated for a further 24h. HEK293 cells expressing both genome-edited CXCR4/LgBiT and β-arrestin2/SmBiT cells were seeded at 100,000 cells/well. On the day of assay, cells were washed and incubated with pre-warmed HBSS containing 0.1% BSA for 30 minutes at 37°C. Cells were pre-incubated with or without AMD3100 (1 μM) for 30 minutes at 37°C before furimazine (10 μM) was added to cells and allowed to equilibrate for 5 minutes. Total luminescence was measured on a PHERAStar FS plate reader, with basal measurements taken before HBSS or half log increasing concentrations of CXCL12 (0.3 nM - 300 nM) were added at time = 0 and total luminescence was measured. In a subset of experiments HEK293 cells expressing genome-edited β-arrestin2/SmBiT or both genome-edited CXCR4/LgBiT and β-arrestin2/SmBiT were additionally transiently transfected with 500 ng per well of a 6 well plate with β-arrestin2/Halotag (unlabelled) and cells were seeded as above. On the day of assay, cells were prepared as above and HBSS or CXCL12 (300 nM) was added at time = zero. Baseline-corrected luminescence was calculated by subtracting the vehicle-treated and/or mean basal luminescence from the ligand-treated luminescence. Basal luminescence for each configuration was calculated from the luminescence measurement immediately before the addition of ligand.

NanoBiT Internalisation/Cell Surface Expression

For end point internalisation/cell surface expression assays, genome-edited or HEK293 cells stably expressing HiBiT/CXCR4, HiBiT/ACKR3 or HiBiT/β_2-adrenoceptors were seeded into poly-D-lysine coated white flat bottom 96 well plates at 30,000 cells/well and incubated for 24h at 37°C/5% CO_2. On the day of the assay, cells were washed and incubated with pre-warmed HBSS supplemented with 0.1% BSA. To generate log concentration response curves, cells expressing HiBiT/CXCR4 were incubated in the absence or presence of CXCL12, AMD3100, IT1t or XAC. HEK293 cells expressing exogenous HiBiT/ACKR3 were incubated in the absence or presence of CXCL12 or CXCL11 and HEK293 cells expressing HiBiT/β_2-adrenceptors were incubated in the absence or presence of isoprenaline or propranolol for 60 minutes at 37°C. To generate CXCL12 concentration response curves in the presence of AMD3100 in membrane preparations, 10 μg membrane protein diluted in HBSS supplemented with 0.1% BSA was loaded into each well containing 0.25 mg/mL saponin. Membranes were then incubated with AMD3100 (1 μM) in the absence or presence of CXCL12 for 60 minutes at 37°C. Following ligand incubation, furimazine (10 μM) and purified LgBiT (10 nM) were added, plates were incubated for 5 minutes and total light emissions were measured using a PHERAStar FS plate reader with the concentration response curves representing the luminescence after 30 minutes.

For kinetic analysis of ligand induced changes in luminescence/cell surface expression, genome-edited or HEK293 cells stably expressing HiBiT/CXCR4 were seeded into poly-D-lysine coated white flat bottom 96 well plates at 30,000 cells/well and incubated for 24h at 37°C/5% CO_2. On the day of assay, cells were washed and incubated with pre-warmed HBSS supplemented with 0.1% BSA. For assays using membrane preparations, 10 μg membrane protein diluted in HBSS supplemented with 0.1% BSA was loaded into each well containing 0.25 mg/mL saponin. 10 nM purified LgBiT was then added to each well and cells incubated for 60 minutes at 37°C. Following ligand incubation, furimazine (10 μM) was added, plates incubated for 5 minutes and total light emissions were measured using a PHERAStar FS plate reader at 37°C for 5 reads before CXCL12, AMD3100, IT1t or XAC were added to cells and measurement of total light emissions was continued. The concentration response curves with membrane preparations were generated in this manner with points representing the luminescence at 30 minutes. Baseline-corrected luminescence was calculated by subtracting vehicle-treated luminescence from the ligand-treated luminescence and/or by subtracting the mean of the pre-ligand addition basal reads from the ligand-treated luminescence.

Determination of NanoBiT Affinity

To investigate the affinity of HiBiT-LgBiT complementation, HEK293 cells expressing genome-edited HiBiT/CXCR4 or HiBiT/β_2-adrenoceptors as well wildtype HEK293 cells were seeded into poly-D-lysine coated white flat bottom 96 well plates at 30,000 cells/well and incubated for 24h at 37°C/5% CO_2. On the day of the assay, cells were washed and incubated with HBSS supplemented with 0.1% BSA 60 minutes at 37°C. Cells were then incubated with increasing concentrations of purified LgBiT for 30 minutes at 37°C. Following LgBiT incubation, furimazine (10 μM) was added, plates incubated for 5 minutes and total light emissions were measured using a PHERAStar FS plate reader. For assays using membrane preparations 10 μg membrane protein from genome-edited HiBiT/CXCR4 cells diluted in HBSS supplemented with 0.1% BSA was loaded into each well containing 0.25 mg/ml saponin. For assays using both purified HiBiT and LgBiT, 1 nM purified HiBiT-control protein (HiBiT-Halotag) diluted in HBSS supplemented with 0.1% BSA was loaded into each well. To determine the effect of AMD3100, samples were then incubated in the absence or presence of AMD3100 (10 μM) for 30 minutes at 37°C. In parallel non-specific luminescence/binding was determined by adding purified LgBiT to wells containing wildtype cells or membranes, or for HiBiT-control protein HBSS containing 0.1% BSA. Following incubation, furimazine (10 μM) was added, plates incubated for 5 minutes and total light emissions measured using a PHERAStar FS plate reader.

QUANTIFICATION AND STATISTICAL ANALYSIS

Data Presentation and Statistical Analysis

Due to differences in NLuc expression between cell lines, optimised plate reader filtered light emission gains were used to ensure sufficient sensitivity and/or measurements acquired did not saturate the detector. Therefore, raw BRET ratios and luminescence values cannot be compared as a measure of BRET efficacy or expression between cells lines or assay conditions. However, gains used to acquire total luminescence for NanoBiT β-arrestin2 recruitment were consistent between assays. In general, BRET ratios were calculated by dividing the acceptor emission by the donor emission. Calculation of baseline-corrected BRET ratios or luminescence values are described in the methods for each assay configuration.

Prism 7 software was used to analyse ligand-binding curves. For NanoBRET receptor-ligand saturation binding assays total and non-specific saturation binding curves were simultaneously fitted using the following equation:

$$BRET\ Ratio = \frac{B_{max} * [B]}{[B] + K_d} + ((M * [B]) + C)$$

where B_{max} is the maximal response, [B] is the concentration of fluorescent ligand in nM, K_d is the equilibrium dissociation constant in nM, M is the slope of the non-specific binding component and C is the intercept with the Y-axis. For HiBiT-LgBiT, complementation affinity was determined as per the NanoBRET saturation binding but with luminescence generated by LgBiT, [B] incubated on wildtype cells or membranes was used as non-specific binding.

Agonist concentration-response data were fitted using the following equation:

$$Response = \frac{E_{max} * [A]}{EC_{50} + [A]}$$

Where E_{max} is the maximum response, EC_{50} is the concentration of agonist required to produce 50% of the maximal response and [A] is the agonist concentration. pEC_{50} calculated at approximately 5 minutes post ligand addition.

Inhibition concentration-response data were fitted using the following equation:

$$Inhibition = \frac{E_{max} * [A]}{IC_{50} + [A]}$$

where [A] is the concentration of competing ligand, E_{max} is the maximum specific binding or response mediated by a probe and IC_{50} is the molar concentration of this competing ligand required to inhibit 50% of the specific response or binding. pK_d, pIC_{50} and pEC_{50} values were calculated as –log K_d, –log IC_{50} and –log EC_{50} respectively.

Quantification of NLuc or HiBiT tagged protein expression was interpolated by Prism from linear regression of a log-log standard curve fitted with the following equation:

$$Y = A + B[X]$$

where [X] is the concentration of NanoLuc or HiBiT, Y is the luminescence output, A is the y-intercept and B is the slope of the line.

Statistical analysis was performed using Prism 7 software (GraphPad, San Diego, USA) using one or two-way ANOVA with an appropriate multiple comparisons tests where required. Specific statistical tests used are indicated in the figure legends and were performed on the mean data of individual experiments (n) also indicated in the figure legends. A p-value <0.05 was considered statistically significant.

DATA AND CODE AVAILABILITY

This study did not generate/analyze any computational datasets/code.

Cell Chemical Biology, Volume *27*

Supplemental Information

CRISPR-Mediated Protein Tagging with Nanoluciferase to Investigate Native Chemokine Receptor Function and Conformational Changes

Carl W. White, Birgit Caspar, Hannah K. Vanyai, Kevin D.G. Pfleger, and Stephen J. Hill

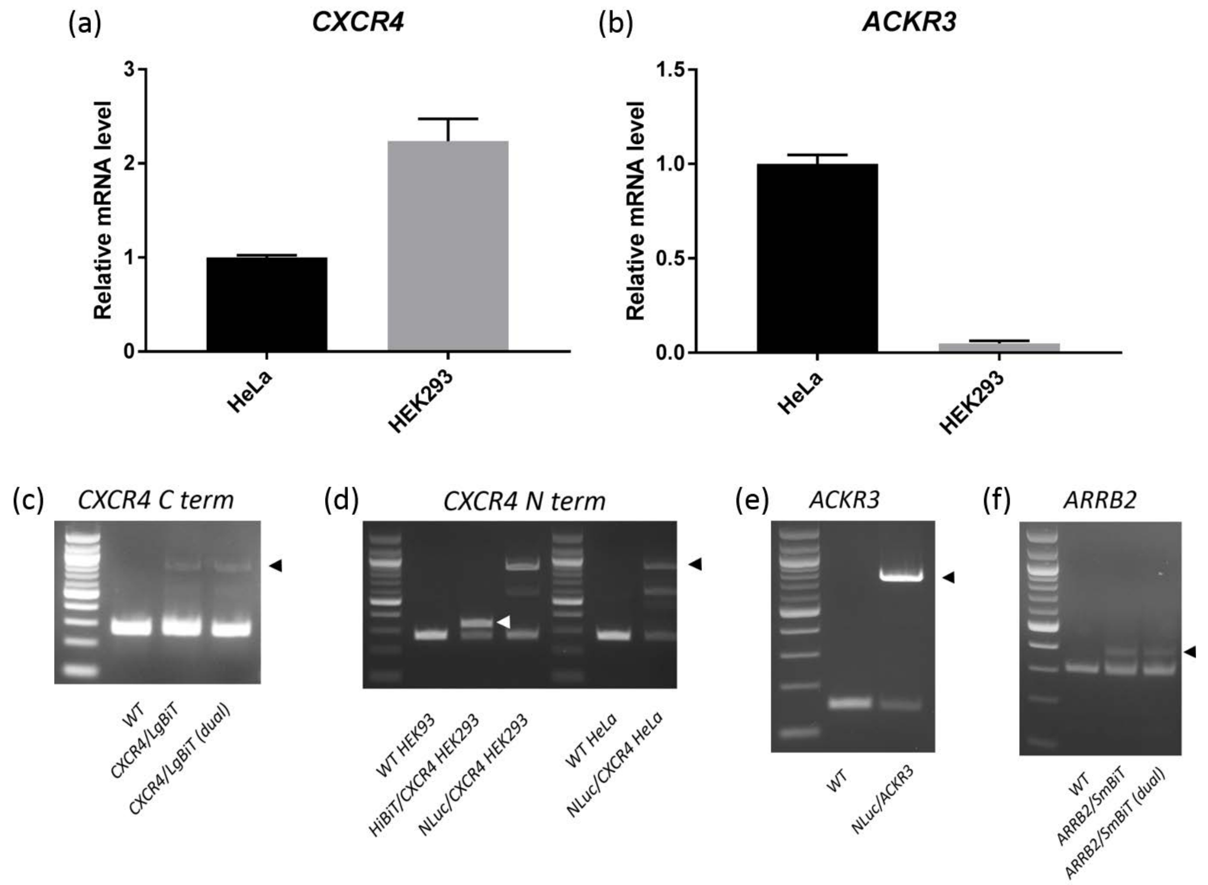

Supplementary Figure S1. (Related to Figure 1). Analysis of wildtype or genome edited cells. **(a)** CXCR4 mRNA expression in HeLa (black bar) or HEK293 (grey bar) cells and **(b)** ACKR3 mRNA expression in HeLa (black bar) or HEK293 (grey bar) cells. **(c-f)** Genome-editing resulted in clonal cell lines heterozygous for the insert. PCR amplification using target specific primers of DNA from: **(c)** wildtype (WT) HEK293 cells, CXCR4/LgBiT clones or cells edited to express both CXCR4/LgBiT and ARRB2/SmBiT (dual), (d) wildtype (WT) HEK293 cells, HiBiT/CXCR4 or Nluc/CXCR4 clones as well as WT HeLa cells and NLuc/CXCR4 HeLa clones, **(e)** wildtype (WT) HeLa cells and NLuc/ACKR3 clones and **(f)** wildtype (WT) HEK293 cells, ARRB2/SmBiT clones or clones edited to express both ARRB2/SmBiT and CXCR4/LgBiT (dual). Wildtype PCR product at 200-300bp. In arrows indicate PCR product of inserted tag. Bars represent mean ± s.e.m. of three experiments.

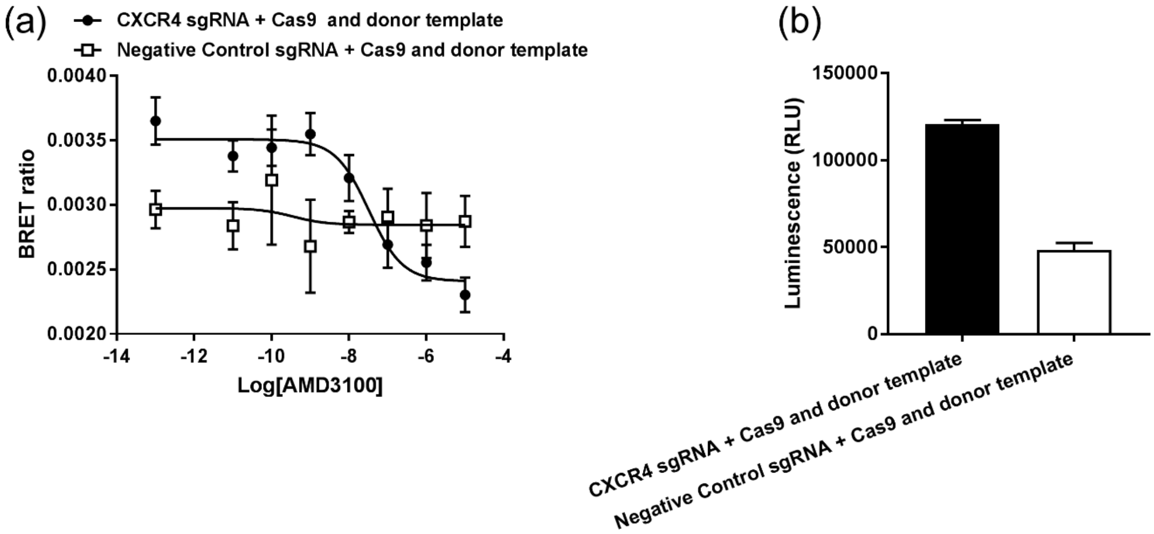

Supplementary Figure S2. (Related to Figure 2). NanoBRET competition ligand binding in a non-clonal pool of HEK293 cells some of which are expressing genome-edited Nluc/CXCR4 **(a)** Displacement of 12.5 nM CXCL12-AF47 binding by AMD3100 (10 pM – 10 µM) in non-clonal pool of cells transfected with plasmids encoding sgRNAs targeted to the CXCR4 N-terminus, Cas9 and NLuc/CXCR4 donor repair template (closed circles) or in non-clonal pool of cells transfected with plasmids encoding non-targeted sgRNAs, Cas9 and NLuc/CXCR4 donor repair template (open circles). **(b)** Luminescence generated from non-clonal pools of cells transfected either with plasmids encoding sgRNAs targeted to the CXCR4 N-terminus (closed bar) or untargeted sgRNA (open bar) in addition to Cas9 and NLuc/CXCR4 donor repair template. Bars or points represent mean ± s.e.m. of five individual experiments performed in triplicate.

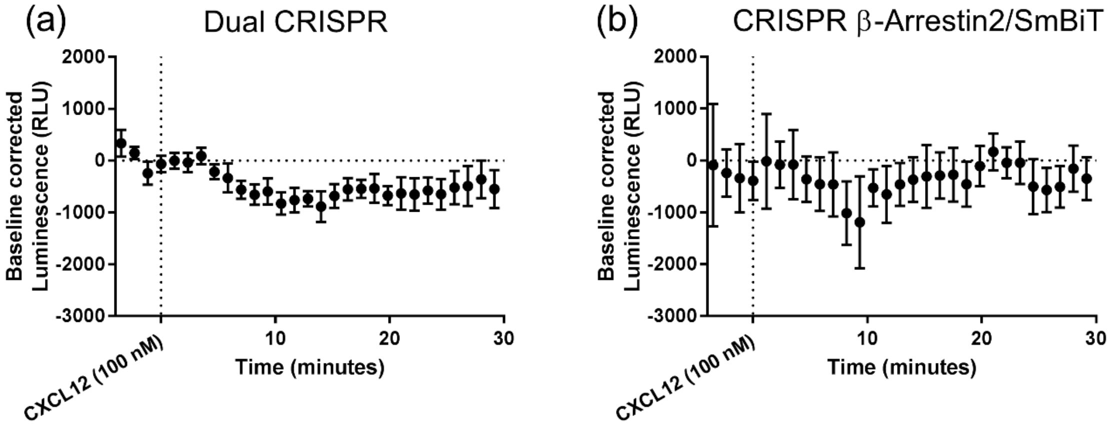

Supplementary Figure S3. (Related to Figure 4). Inhibition of CXCL12-mediated recruitment of genome-edited β-arrestin2/SmBiT recruitment to CXCR4/LgBiT by over-expression of β-arrestin2. HEK293 cells expressing **(a)** both genome-edited CXCR4/LgBiT and β-arrestin2/SmBiT (Dual CRISPR) or genome-edited β-arrestin2/SmBiT transiently transfected with CXCR4/LgBiT (CRISPR β-arrestin2/SmBiT) were transfected with 500 ng per well of a 6 well plate of β-arrestin2/Halotag (unlabelled). At time zero CXCL12 (100 nM) or HBSS was applied to the cells and luminescence changes in luminescence over the baseline was measured. Points represent mean ± s.e.m. of four **(a)** or three **(b)** individual experiments performed with replicates of 4-6. Baseline-corrected luminescence calculated as described in *Methods*.

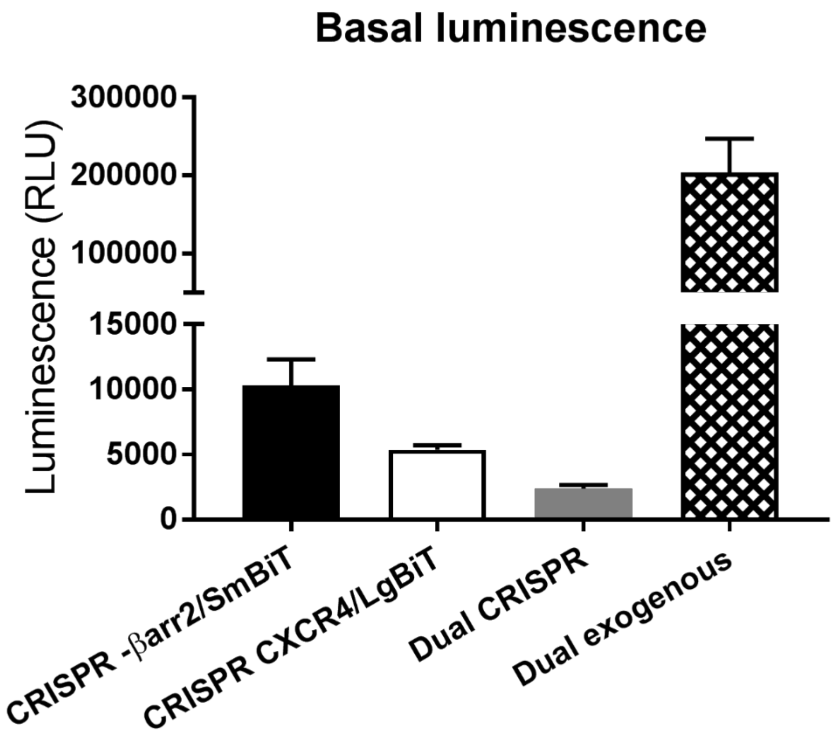

Supplementary Figure S4 (Related to Figure 4). Assessment of the effect of assay configuration on observations. Basal luminescence observed following application of furimazine (10 µM) in HEK293 cells expressing gene-edited β-arrestin2/SmBiT transiently transfected with CXCR4/LgBiT (CRISPR β-arr2/SmBiT, closed bar), genome-edited CXCR4/LgBiT transiently transfected with β-arrestin2/SmBiT (CRISPR CXCR4/LgBiT, open bar), both genome-edited CXCR4/LgBiT and gene-edited β-arrestin2/SmBiT (Dual CRISPR, grey bar), or HEK293 cells expressing transiently transfected CXCR4/LgBiT and β-arrestin2/SmBiT (Dual exogenous, hatched bar). Points or bars represent mean ± s.e.m. of six (CRISPR CXCR4/LgBiT and Dual exogenous), seven (CRISPR β-arrestin2/SmBiT) or eight (Dual CRISPR) individual experiments performed in triplicate.

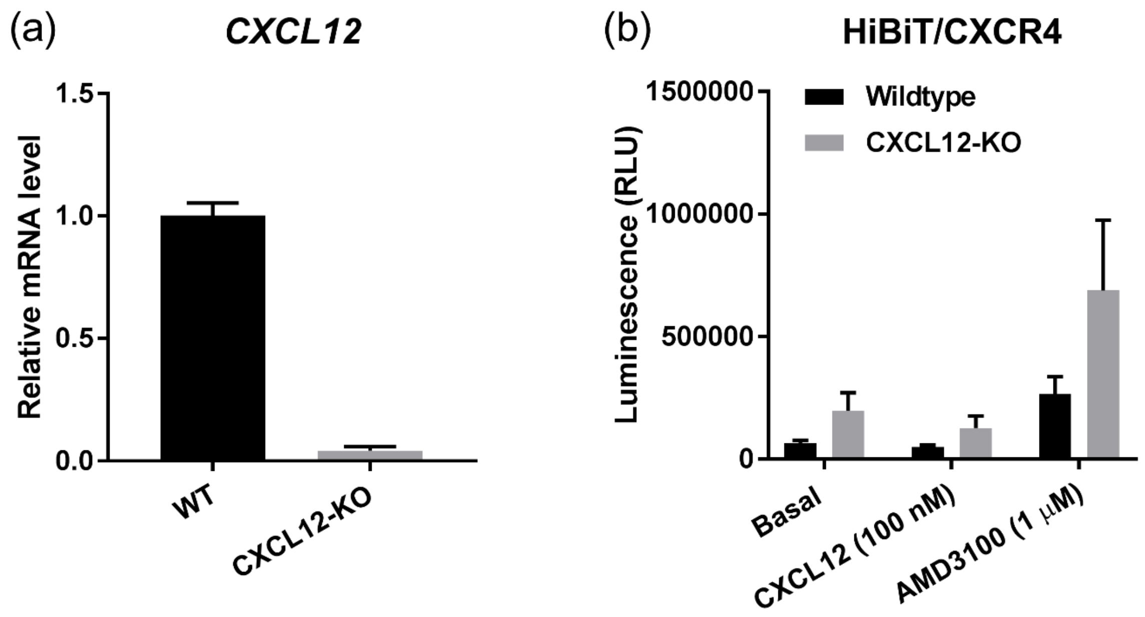

Supplementary Figure S5. (Related to Figure 5). (a) Analysis of *CXCL12* mRNA expression in wildtype (black bars) or genome-edited CXCL12 knockout HEK293 cells (grey bars). Bars represent mean ± S.D. of mRNA analysis from a single clone performed in triplicate. **(b) Determination of the effect of AMD3100 in the absence of endogenous CXCL12.** Change in luminescence in live wildtype HEK293 cells (black bar) or genome-edited CXCL12-KO HEK293 cells (grey bar) transiently transfected with HiBiT/CXCR4 in the absence (basal) or presence of CXCL12 (100 nM) or AMD3100 (1 µM). Luminescence generated by the addition of purified LgBiT (10 nM) and furimazine. Bars represent mean ± s.e.m. of four experiments performed in triplicate.

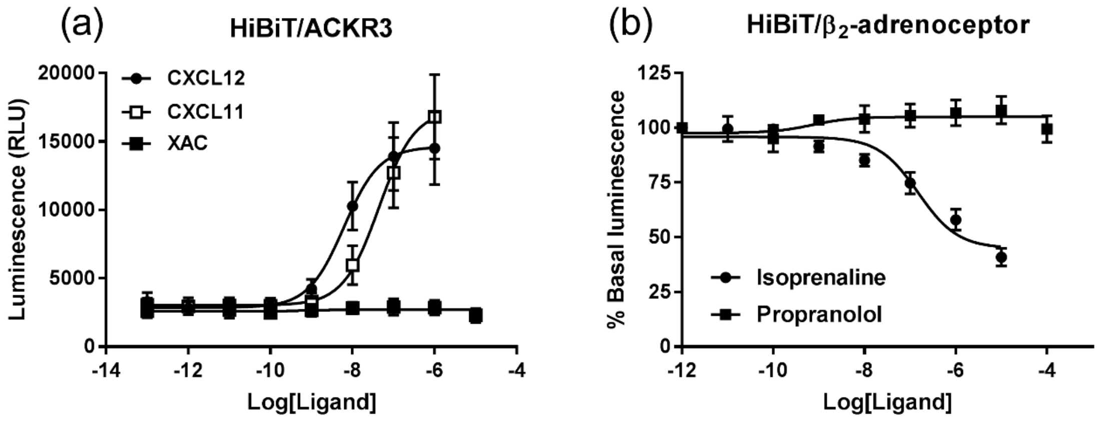

Supplementary Figure S6. (Related to Figure 5). Effect of ligand addition to HiBiT-tagged receptors. **(a)** HEK293 cells exogenously expressing HiBiT/ACKR3 were incubated in the absence or presence of increasing concentrations of CXCL12 (closed circles), CXCL11 (open squares) or XAC (closed squares). **(b)** HEK293 cells expressing genome-edited HiBiT/β_2-adrenoceptors were incubated in the absence or presence of increasing concentrations of isoprenaline (closed circles) or propranolol (closed squares). Ligands were incubated for 1 hour at 37 °C before luminescence was generated by the addition of furimazine (10 µM) and purified LgBiT (10 nM). Points represent mean ± s.e.m. luminescence or % baseline luminescence of five experiments performed in triplicate.

Supplementary Table 1: Primers used for in this study (Related to STAR Methods)

**Primer[1]**	**Sequence**	**Target/Purpose**
ACKR3N sgRNA1 fwd	CACCGATTGCCCGCCTCAGAACGA	N-terminally tagging ACKR3/ sgRNA construction
ACKR3N sgRNA1 rvs	AAACTCGTTCTGAGGCGGGCAATC	
ACKR3N sgRNA2 fwd	CACCGATGCAGATCCATCGTTCTG	
ACKR3N sgRNA2 rvs	AAACCAGAACGATGGATCTGCATC	
CXCR4N sgRNA1 fwd	CACCGATCCCCTCCATGGTAACCGC	N-terminally tagging CXCR4/ sgRNA construction
CXCR4N sgRNA1 rvs	AAACGCGGTTACCATGGAGGGGATC	
CXCR4N sgRNA2 fwd	CACCGTGGAGAACCAGCGGTTACCA	
CXCR4N sgRNA2 rvs	AAACTGGTAACCGCTGGTTCTCCAC	
CXCL12_KO_Sg1_Fwd	CACCGGCATGGGCATCTGTAGCTC	CXCL12 knockout/ sgRNA construction
CXCL12_KO_Sg1_Rvs	AAACGAGCTACAGATGCCCATGCC	
CXCL12_KO_Sg2_Fwd	CACCGCATCTGTAGCTCAGGCTGAC	
CXCL12_KO_Sg2_Rvs	AAACGTCAGCCTGAGCTACAGATGC	
ADRB2_sgRNA1_Fwd	CACCGCCTGCCAGACTGCGCGCCAT	N-terminally tagging ADRB2/ sgRNA construction
ADRB2_sgRNA1_Rvs	AAACATGGCGCGCAGTCTGGCAGG	
ADRB2_sgRNA2_Fwd	CACCGTTGCCCCATGGCGCGCAGTC	
ADRB2_sgRNA2_Rvs	AACGACTGCGCGCCATGGGGCAA	
ARRB2_Screen_Fwd	CCTCACCTCACAACCCTCTT	PCR genotype/ screen C-terminally tagged *ARRB2*
ARRB2_Screen_Rvs	CAAGAGAGGGTCCAGTGTGT	
CXCR4_Screen_C_Fwd	TCTCCAAAGGAAAGCGAGGT	PCR genotype/ screen C-terminally tagged *CXCR4*
CXCR4_Screen_C_Rvs	TCCTGCCTAGACACACATCA	
ACKR3HDRTSeq	TAGGTCATTTGATTGCCCGC	PCR genotype/ screen C-terminally tagged *ACKR3*
ACKR3_N_Screen_Rvs	CGCTTTTGTTGGGCATGTTG	
5`CXCR4NInt_Seq	TTTATAAAAGTCCGGCCGCG	PCR genotype/ screen N-terminally tagged *CXCR4*
3`CXCR4NInt_Seq	ACTCCTTTCGGTGACCCTTT	
Hu_CXCR4_Fwd	AACGTCAGTGAGGCAGATGA	*CXCR4* RTqPCR primer
Hu_CXCR4_Rvs	GCCAACCATGATGTGCTGAA	*CXCR4* RTqPCR primer
Hu_ACKR3_Fwd	CTCAGCACTAAGGGAGCCAG	*ACKR3* RTqPCR primer
Hu_ACKR3_Rvs	AGATCCATCGTTCTGAGGCG	*ACKR3* RTqPCR primer
huARRB2_RTqPCR_Fwd	TCCATGCTCCGTCACACTG	*ARRB2* RTqPCR primer
huARRB2_RTqPCR_Rev	ACAGAAGGCTCGAATCTCAAAG	*ARRB2* RTqPCR primer

Supplementary Table 2: Repair template sequences used in this study (Related to STAR Methods)

Target	Sequence
Tagging *CXCR4* on the N-terminus with NLuc	CAGAATTATGCCAAATCCTACCTTCTTCTGAAAGTATCTCCTAATTATCTGCACCTGACC<br>CTAGTGATGCTGTGAATGTGCAAGTATAGCTACATCCTCCGAAGGAAGGATCTTTACTC<br>CTTTTACCTCCTGAATGGGCTGCGTCTGCTGAAAGCGCGGGGAATGGCGTTGGAAGCTT<br>GGCCCTACTTCCAGCATTGCCGCCTACTGGTTGGGTTACTCCAGCAAGTCACTCCCCTTC<br>CCTGGGCCTCAGTGTCTCTACTGTAGCATTCCCAGGTCTGGAATTCCATCCACTTTAGCA<br>AGGATGGACGCGCCACAGAGAGACGCGTTCCTAGCCCGCGCTTCCCACCTGTCTTCAGG<br>CGCATCCCGCTTCCCTCAAACTTAGGAAATGCCTCTGGGAGGTCCTGTCCGGCTCCGGA<br>CTCACTACCGACCACCCGCAAACAGCAGGGTCCCCTGGGCTTCCCAAGCCGCGCACCTC<br>TCCGCCCCGCCCCTGCGCCCTCCTTCCTCGCGTCTGCCCCTCTCCCCCACCCCGCCTTCTC<br>CCTCCCCGCCCCAGCGGCGCATGCGCCGCGCTCGGAGCGTGTTTTTATAAAAGTCCGGC<br>CGCGGCCAGAAACTTCAGTTTGTTGGCTGCGGCAGCAGGTAGCAAAGTGACGCCGAGG<br>GCCTGAGTGCTCCAGTAGCCACCGCATCTGGAGAACCAGCGGTTACCATGAGGTTGTGT<br>ATTCCGCAAGTGTTGCTCGCTTTGTTCCTCTCTATGTTGACAGGTCCTGGGGAAGGCTCT<br>CGCAAGCTCCTGGTCTTCACACTCGAAGATTTCGTTGGGGACTGGCGACAGACAGCCGG<br>CTACAACCTGGACCAAGTCCTTGAACAGGGAGGTGTGTCCAGTTTGTTTCAGAATCTCG<br>GGGTGTCCGTAACTCCGATCCAAAGGATTGTCCTGAGCGGTGAAAATGGGCTGAAGATC<br>GACATCCATGTCATCATCCCGTATGAAGGTCTGAGCGGCGACCAAATGGGCCAGATCGA<br>AAAAATTTTTAAGGTGGTGTACCCTGTGGATGATCATCACTTTAAGGTGATCCTGCACT<br>ATGGCACACTGGTAATCGACGGGGTTACGCCGAACATGATCGACTATTTCGGACGGCCG<br>TATGAAGGCATCGCCGTGTTCGACGGCAAAAAGATCACTGTAACAGGGACCCTGTGGA<br>ACGGCAACAAAATTATCGACGAGCGCCTGATCAACCCCGACGGCTCCCTGCTGTTCCGA<br>GTAACCATCAACGGAGTGACCGGCTGGCGGCTGTGCGAACGCATTCTGGCGGGATCCCT<br>GGAGGGGATCAGTGTAAGTCCAGTTTCAACCTGCTTTGTCATAAATGTACAAACGTTTG<br>AACTTAGAGCGCAGCCCCTCTCCGAGCGGGCAGAAGCGGCCAGGACATTGGAGGTACC<br>CGTACTCCAAAAAAGGGTCACCGAAAGGAGTTTTCTTGACCATGCCTATATAGTGCGGG<br>TGGGTGGGGGGGGAGCAGGATTGGAATCTTTTTCTCTGTGAGTCGAGGAGAAACGACT<br>GGAAAGAGCGTTCCAGTGGCTGCATGTGTCTCCCCCTTGAGTCCCGCCGCGCGCGGCGG<br>CTTGCACGCTGTTTGCAAACGTAAGAACATTCTGTGCACAAGTGCAGAGAAGGCGTGCG<br>CGCTGCCTCGGGACTCAGACCACCGGTCTCTTCCTTGGGGAAGCGGGGATGTCTTGGAG<br>CGAGTTACATTGTCTGAATTTAGAGGCGGAGGGCGGCGTGCCTGGGCTGAGTTCCCAGG<br>AGGAGATTGCGCCCGCTTTAACTTCGGGGTTAAGCGCCTGGTGACTGTTCTTGACACTG<br>GGTGCGTGTTTGTTAAACTCTGTGCGGCCGACGGAGCTGTGCCAGTCTCCCAGCACAGT<br>AGGCAGAGGGCGGGAGAGGCGGGTGGACCCACCGCGCCGATCCTCTGAGGGGATCGAG<br>TGGTGGCAGCAGCTAGGAGTTGATCCGCCCGCGCGCTTTGGGTTTGAGGGGG
Tagging *CXCR4* on the N-terminus with HiBiT	CAGAATTATGCCAAATCCTACCTTCTTCTGAAAGTATCTCCTAATTATCTGCACCTGACC<br>CTAGTGATGCTGTGAATGTGCAAGTATAGCTACATCCTCCGAAGGAAGGATCTTTACTC<br>CTTTTACCTCCTGAATGGGCTGCGTCTGCTGAAAGCGCGGGGAATGGCGTTGGAAGCTT<br>GGCCCTACTTCCAGCATTGCCGCCTACTGGTTGGGTTACTCCAGCAAGTCACTCCCCTTC<br>CCTGGGCCTCAGTGTCTCTACTGTAGCATTCCCAGGTCTGGAATTCCATCCACTTTAGCA<br>AGGATGGACGCGCCACAGAGAGACGCGTTCCTAGCCCGCGCTTCCCACCTGTCTTCAGG<br>CGCATCCCGCTTCCCTCAAACTTAGGAAATGCCTCTGGGAGGTCCTGTCCGGCTCCGGA<br>CTCACTACCGACCACCCGCAAACAGCAGGGTCCCCTGGGCTTCCCAAGCCGCGCACCTC<br>TCCGCCCCGCCCCTGCGCCCTCCTTCCTCGCGTCTGCCCCTCTCCCCCACCCCGCCTTCTC<br>CCTCCCCGCCCCAGCGGCGCATGCGCCGCGCTCGGAGCGTGTTTTTATAAAAGTCCGGC<br>CGCGGCCAGAAACTTCAGTTTGTTGGCTGCGGCAGCAGGTAGCAAAGTGACGCCGAGG<br>GCCTGAGTGCTCCAGTAGCCACCGCATCTGGAGAACCAGCGGgTACCATGGTGAGCGGC<br>TGGCGGCTGTTCAAGAAGATTAGCGGGAGTTCTGGCGGCTCGAGCGGTGGATCCCTGGA<br>GGGGATCAGTGTAAGTCCAGTTTCAACCTGCTTTGTCATAAATGTACAAACGTTTGAAC<br>TTAGAGCGCAGCCCCTCTCCGAGCGGGCAGAAGCGGCCAGGACATTGGAGGTgCCCGTA<br>CTCCAAAAAAGGGTCACCGAAAGGAGTTTTCTTGACCATGCCTATATAGTGCGGGTGGG<br>TGGGGGGGGAGCAGGATTGGAATCTTTTTCTCTGTGAGTCGAGGAGAAACGACTGGAA<br>AGAGCGTTCCAGTGGCTGCATGTGTCTCCCCCTTGAGTCCCGCCGCGCGCGGCGGCTTG<br>CACGCTGTTTGCAAACGTAAGAACATTCTGTGCACAAGTGCAGAGAAGGCGTGCGCGCT<br>GCCTCGGGACTCAGACCACCGGTCTCTTCCTTGGGGAAGCGGGGATGTCTTGGAGCGAG<br>TTACATTGTCTGAATTTAGAGGCGGAGGGCGGCGTGCCTGGGCTGAGTTCCCAGGAGGA<br>GATTGCGCCCGCTTTAACTTCGGGGTTAAGCGCCTGGTGACTGTTCTTGACACTGGGTGC<br>GTGTTTGTTAAACTCTGTGCGGCCGACGGAGCTGTGCCAGTCTCCCAGCACAGTAGGCA<br>GAGGGCGGGAGAGGCGGGTGGACCCACCGCGCCGATCCTCTGAGGGGATCGAGTGGTG<br>GCAGCAGCTAGGAGTTGATCCGCCCGCGCGCTTTGGGTTTGAGGGGG

Tagging *ACKR3* on the N-terminus NLuc	AAAAACAGGTGCTGACCCAAAGCTTGAGACAGCTGGTTGGTTGCCCTTCGTTGAAAACC<br>ACACGGCCAACCCAGAGTCTTCAGGTTTGGGGCGCAGTTAAGCTGTGGGGCACCCTGCG<br>TAGGCTCACTGTGTTTCCAGAGGCTTTATGACTGCACACTGGGGCCGCCTGAGACCCAG<br>GTTTATATGTGAATTTAGCAGACAAGTAAAAAGTGCTGAGTCACATTATAAATGCAAAT<br>TTGGAGGAGGATTTGAAATGCTTTCATTAAGACACCAAAGCAGGACACTTGTAATACAG<br>CAGTGCCAGGAGAGGACAACCCTGCCATTCTTTGGGGACCTAGATAAAATGGCTCCACT<br>GTCCCCTGCTGAGAGTGAGCAGGCCATCAGCATCGCGGTGGCCGGGGCTCAGCTGCCTG<br>ACGTTTCTTTGGCCCCTGTACTGTGTTGATTTCACACACTGGGCACAGCCATGCTGGCGG<br>TGTCTTGAGAACACAGCCATGAAAAGATGGAGAAAGCAAACACAGTTTCTGTCTTGGA<br>GCACCACATGGATTTAGATTCAGAGTATGATTTATTTTGTTTTGGAGAGTTTTATTATGA<br>ATTTTTTTTCTCCCTTCTTTGCAAAGACATTACAGAGCTAAACCCAAACAGCTGAGCCTA<br>TCAGGGCCTTGGAAAAGCATTTTTGCCTGAAACTTGAGTTTTTCCTAATGAAGTGACTTT<br>CCTCTTCCATCTTTTTTGTTTGCTTGGTTTTCTCATAGGTCATTTGATTGCCCGCCTCAGA<br>ACGGGTACCATGCGGCTCTGCATCCCGCAGGTGCTGTTGGCCTTGTTCCTTTCCATGCTG<br>ACAGGGCCGGGAGAAGGCAGCCGGAAGCTTCTGGTCTTCACACTCGAAGATTTCGTTGG<br>GGACTGGCGACAGACAGCCGGCTACAACCTGGACCAAGTCCTTGAACAGGGAGGTGTG<br>TCCAGTTTGTTTCAGAATCTCGGGGTGTCCGTAACTCCGATCCAAAGGATTGTCCTGAGC<br>GGTGAAAATGGGCTGAAGATCGACATCCATGTCATCATCCCGTATGAAGGTCTGAGCGG<br>CGACCAAATGGGCCAGATCGAAAAAATTTTTAAGGTGGTGTACCCTGTGGATGATCATC<br>ACTTTAAGGTGATCCTGCACTATGGCACACTGGTAATCGACGGGGTTACGCCGAACATG<br>ATCGACTATTTCGGACGGCCGTATGAAGGCATCGCCGTGTTCGACGGCAAAAAGATCAC<br>TGTAACAGGGACCCTGTGGAACGGCAACAAAATTATCGACGAGCGCCTGATCAACCCC<br>GACGGCTCCCTGCTGTTCCGAGTAACCATCAACGGAGTGACCGGCTGGCGGCTGTGCGA<br>ACGCATTCTGGCGGGATCCGATCTGCATCTCTTCGACTACTCAGAGCCAGGGAACTTCT<br>CGGACATCAGCTGGCCATGCAACAGCAGCGACTGCATCGTGGTGGACACGGTGATGTG<br>TCCCAACATGCCCAACAAAAGCGTCCTGCTCTACACGCTCTCCTTCATTTACATTTTCAT<br>CTTCGTCATCGGCATGATTGCCAACTCCGTGGTGGTCTGGGTGAATATCCAGGCCAAGA<br>CCACAGGCTATGACACGCACTGCTACATCTTGAACCTGGCCATTGCCGACCTGTGGGTT<br>GTCCTCACCATCCCAGTCTGGGTGGTCAGTCTCGTGCAGCACAACCAGTGGCCCATGGG<br>CGAGCTCACGTGCAAAGTCACACACCTCATCTTCTCCATCAACCTCTTCGGCAGCATTTT<br>CTTCCTCACGTGCATGAGCGTGGACCGCTACCTCTCCATCACCTACTTCACCAACACCCC<br>CAGCAGCAGGAAGAAGATGGTACGCCGTGTCGTCTGCATCCTGGTGTGGCTGCTGGCCT<br>TCTGCGTGTCTCTGCCTGACACCTACTACCTGAAGACCGTCACGTCTGCGTCCAACAATG<br>AGACCTACTGCCGGTCCTTCTACCCCGAGCACAGCATCAAGGAGTGGCTGATCGGCATG<br>GAGCTGGTCTCCGTTGTCTTGGGCTTTGCCGTTCCCTTCTCCATTATCGCTGTCTTCTACT<br>TCCTGCTGGCCAGAGCCATCTCGGCGTCCAGTGACCAGGAGAAGCACAGCAGCCGGAA<br>GATCATCTTCTCCTACGTGGTGG
Tagging *ADRB2* on the N-terminus with HiBiT (Synthesised as a ssODN)	GGCCCGCAGAGCCCCGCCGTGGGTCCGCCCGCTGAGGCGCCCCCAGCCAGTGCGCTCAC<br>CTGCCAGACTGCGCGCCATGGTGAGCGGCTGGCGGCTGTTCAAGAAGATTAGCGGGAG<br>TTCTGGCGGGCAACCCGGGAACGGCAGCGCCTTCTTGCTGGCACCCAATGGAAGCCATG<br>CGCCGGACCACGACGTCACGCAGG
Tagging *CXCR4* on the C-terminus with LgBiT (Repair template described in White et al, 2017)	TCTTTGTCATCACGCTTCCCTTCTGGGCAGTTGATGCCGTGGCAAACTGGTACTTTGGGA<br>ACTTCCTATGCAAGGCAGTCCATGTCATCTACACAGTCAACCTCTACAGCAGTGTCCTC<br>ATCCTGGCCTTCATCAGTCTGGACCGCTACCTGGCCATCGTCCACGCCACCAACAGTCA<br>GAGGCCAAGGAAGCTGTTGGCTGAAAAGGTGGTCTATGTTGGCGTCTGGATCCCTGCCC<br>TCCTGCTGACTATTCCCGACTTCATCTTTGCCAACGTCAGTGAGGCAGATGACAGATAT<br>ATCTGTGACCGCTTCTACCCCAATGACTTGTGGGTGGTTGTGTTCCAGTTTCAGCACATC<br>ATGGTTGGCCTTATCCTGCCTGGTATTGTCATCCTGTCCTGCTATTGCATTATCATCTCCA<br>AGCTGTCACACTCCAAGGGCCACCAGAAGCGCAAGGCCCTCAAGACCACAGTCATCCT<br>CATCCTGGCTTTCTTCGCCTGTTGGCTGCCTTACTACATTGGGATCAGCATCGACTCCTT<br>CATCCTCCTGGAAATCATCAAGCAAGGGTGTGAGTTTGAGAACACTGTGCACAAGTGGA<br>TTTCCATCACCGAGGCCCTAGCTTTCTTCCACTGTTGTCTGAACCCCATCCTCTATGCTTT<br>CCTTGGAGCCAAATTTAAAACCTCTGCCCAGCACGCACTCACCTCTGTGAGCAGAGGGT<br>CCAGCCTCAAGATCCTCTCCAAAGGAAAGCGAGGTGGACATTCATCTGTTTCGACTGAG<br>TCTGAGTCTTCAAGTTTTCACTCCAGCGCGGGACTCGAGAATTCTGGCTCCAGCGGTGG<br>TGGCGGGAGCGGAGGTGGAGGGTCGTCAGGTGTCTTCACACTCGAAGATTTCGTTGGGG<br>ACTGGGAACAGACAGCCGCCTACAACCTGGACCAAGTCCTTGAACAGGGAGGTGTGTC<br>CAGTTTGCTGCAGAATCTCGCCGTGTCCGTAACTCCGATCCAAAGGATTGTCCGGAGCG<br>GTGAAAATGCCCTGAAGATCGACATCCATGTCATCATCCCGTATGAAGGTCTGAGCGCC<br>GACCAAATGGCCCAGATCGAAGAGGTGTTTAAGGTGGTGTACCCTGTGGATGATCATCA<br>CTTTAAGGTGATCCTGCCCTATGGCACACTGGTAATCGACGGGGTTACGCCGAACATGC<br>TGAACTATTTCGGACGGCCGTATGAAGGCATCGCCGTGTTCGACGGCAAAAAGATCACT<br>GTAACAGGGACCCTGTGGAACGGCAACAAAATTATCGACGAGCGCCTGATCACCCCCG<br>ACGGCTGCATGCTGTTCCGAGTAACCATCAACAGCTAATCTAGAGGGCCCCACAGATGT<br>AAAAGACTTTTTTTTATACGATAAATAACTTTTTTTTAAGTTACACATTTTTCAGATATA

	AAAGACTGACCAATATTGTACAGTTTTTATTGCTTGTTGGATTTTTGTCTTGTGTTTCTTT<br>AGTTTTTGTGAAGTTTAATTGACTTATTTATATAAATTTTTTTTGTTTCATATTGATGTGT<br>GTCTAGGCAGGACCTGTGGCCAAGTTCTTAGTTGCTGTATGTCTCGTGGTAGGACTGTA<br>GAAAAGGGAACTGAACATTCCAGAGCGTGTAGTGAATCACGTAAAGCTAGAAATGATC<br>CCCAGCTGTTTATGCATAGATAATCTCTCCATTCCCGTGGAACGTTTTTCCTGTTCTTAA<br>GACGTGATTTTGCTGTAGAAGATGGCACTTATAACCAAAGCCCAAAGTGGTATAGAAAT<br>GCTGGTTTTTCAGTTTTCAGGAGTGGGTTGATTTCAGCACCTACAGTGTACAGTCTTGTA<br>TTAAGTTGTTAATAAAAGTACATGTTAAACTTACTTAGTGTTATGTTCTGATTTCTGTTG<br>ACATTCTTTTGGCTAGTAGAAGACAAAAGTAATACATTTATGGTATGCAAAGCACTATC<br>CTAGGTATTTCATTGTAATATTTTACTTACCCCTTATCACAACTCTGATAGATTCTGCTTC<br>TGTTACTAATTACATTTTATAGAAGAGGAAACGGAGGCACAGAAAGCCTAAGTAACTTG<br>GTTAAAGGCATGTAGTAAGTATCAAATCCTGTATTTTAAACCAGGTAACATGACTTAAC<br>GAATCTGAAGCCTTC
Tagging *ARRB2* on the C-terminus with SmBiT	GAGGCGGGTGAGTGTCATGGGGGAGCCTGGGTGGGGGTCACACTGGCTCTCTCTAGTCC<br>CATGTCGTCGTCCTCTTCACGATGCCTCTCCCCTTCCCCAGGGATGTCTCTGTGGAGCTG<br>CCTTTTGTTCTTATGCACCCCAAGCCCCACGACCACATCCCCCTCCCCAGACCCCAGTCA<br>GGTGAGCACACTACCCACCCCAAGCCCTCAGAGGGAGGGCCTGAAGCAGGGCCAGTGG<br>AGGAAAACTGGCCCTTCCAGCACCCACCCCCACACCCCCTCTTCCCGTCCCCCCAGCCG<br>CTCCGGAGACAGATGTCCCTGTGGACACCAACCTCATTGAATTTGATACCAAGTAAGAA<br>ACTCATTCCCCTACTTGACCCTCTTGGGACAAAGATTCCTATAACATTCAAATCTGCCCT<br>CATACCTCTTCCTTGCTTTTGGTGGGGAGAAGCGGATTGTAGCATCAAATCAAGATGCC<br>TTAGCCTTGTGAGGCTGCCTCTTGCTGCCTTTTCTTTGTCCCTTCCTGTAAATACCTCTGG<br>TCCCACTGCTGTTCGAACGCCTCTGTCCCAGAGGCCTAGCTTCGGGGAGGGCAGGGAGT<br>GGGAGGCTGGGACAAGAGTCAGAAGCCCTCACCTCACAACCCTCTTTCCCACCACCAAG<br>CTATGCCACAGATGATGACATTGTGTTTGAGGACTTTGCCCGGCTTCGGCTGAAGGGGA<br>TGAAGGATGACGACTATGATGATCAACTCTGCCTCGAGGGTGGTGGCGGGAGCGGAGG<br>TGGAGGGTCGTCAGGTGTGACCGGCTACCGGCTGTTCGAGGAGATTCTGTAATCTAGAG<br>AAGCGGGGTGGGAAGAAGGGAGGGGATGGGGTTGGGAGAGGTGAGGGCAGGATTAAG<br>ATCCCCACTGTCAATGGGGGATTGTCCCAGCCCCTCTTCCCTTCCCCTCACCTGGAAGCT<br>TCTTCAACCAATCCCTTCACACTCTCTCCCCCATCCCCCCAAGATACACACTGGACCCTC<br>TCTTGCTGAATGTGGGCATTAATTTTTTGACTGCAGCTCTGCTTCTCCAGCCCCGCCGTG<br>GGTGGCAAGCTGTGTTCATACCTAAATTTTCTGGAAGGGGACAGTGAAAAGAGGAGTG<br>ACAGGAGGGAAAGGGGGAGACAAAACTCCTACTCTCAACCTCACACCAACACCTCCCA<br>TTATCACTCTCTCTGCCCCCATTCCTTCAAGAGGAGACCCTTTGGGGACAAGGCCGTTTC<br>TTTGTTTCTGAGCATAAAGAAGAAAATAAATCTTTTACTAAGCATGAGTGTGTGTTTTCT<br>CTGTAGTGTTTAGAGAGTGTATGGTGTGCTTATCCATGATTCTGTTAGCTTGTGGGGGAG<br>GACCCAGTCTCTCTTCTGCACCCAAAAGCCACCTGACACGGAGGCTATCAGCAAATGTT<br>TATTGGATGACTGTGCTGTGGCTGTGTTCCAGGGTAAACATCAAAACTTGACTTAGTCC<br>AAACTCAAGGGTGAGGCTTGATAAGGCCACAAAGTGAACTTAGTCATT
